# Supplementary material for: Cohesin Disrupts Polycomb-Dependent Chromosome Interactions in Embryonic Stem Cells
Source: Cell Rep. 2020 Jan 21;30(3):820–835.e10. doi: 10.1016/j.celrep.2019.12.057 (PMC6988126; doi:10.1016/j.celrep.2019.12.057)
Supplement: Document S1. Figures S1–S9 and Table S1 [file mmc1.pdf]

Cell Reports, Volume *30*

Supplemental Information

Cohesin Disrupts Polycomb-Dependent Chromosome Interactions in Embryonic Stem Cells

James D.P. Rhodes, Angelika Feldmann, Benjamín Hernández-Rodríguez, Noelia Díaz, Jill M. Brown, Nadezda A. Fursova, Neil P. Blackledge, Praveen Prathapan, Paula Dobrinic, Miles K. Huseyin, Aleksander Szczurek, Kai Kruse, Kim A. Nasmyth, Veronica J. Buckle, Juan M. Vaquerizas, and Robert J. Klose

Supplemental Items

Supplementary Figure 1 - Characterisation of SCC1^{DEG} ESCs. Related to Figure 1

Supplementary Figure 2 - Characterisation of RING1B^{DEG} ESCs. Related to Figure 2

Supplementary Figure 3 - Cohesin removal strengthens long-range polycomb chromatin domain interactions. Related to Figure 3

Supplementary Figure 4 – Increased interaction between polycomb chromatin domains is not due to altered cell cycle distribution. Related to Figure 3

Supplementary Figure 5 - Cohesin counteracts polycomb chromatin domain interactions independently of CTCF and insulation. Related to Figure 5

Supplementary Figure 6 - Polycomb chromatin domain interactions are disrupted by cohesin. Related to Figure 6

Supplementary Figure 7 - Polycomb chromatin domain interactions are disrupted by cohesin. Related to Figure 6

Supplementary Figure 8 - Increased polycomb chromatin domain association in the absence of cohesin suppresses gene expression. Related to Figure 7

Supplementary Figure 9 – A model for disruption of polycomb chromatin domain interactions by cohesin and loop extrusion. Related to Figures 1-7

Supplementary Table 1 – Datasets analyzed in this study. Related to Figures 1, 4 and 5

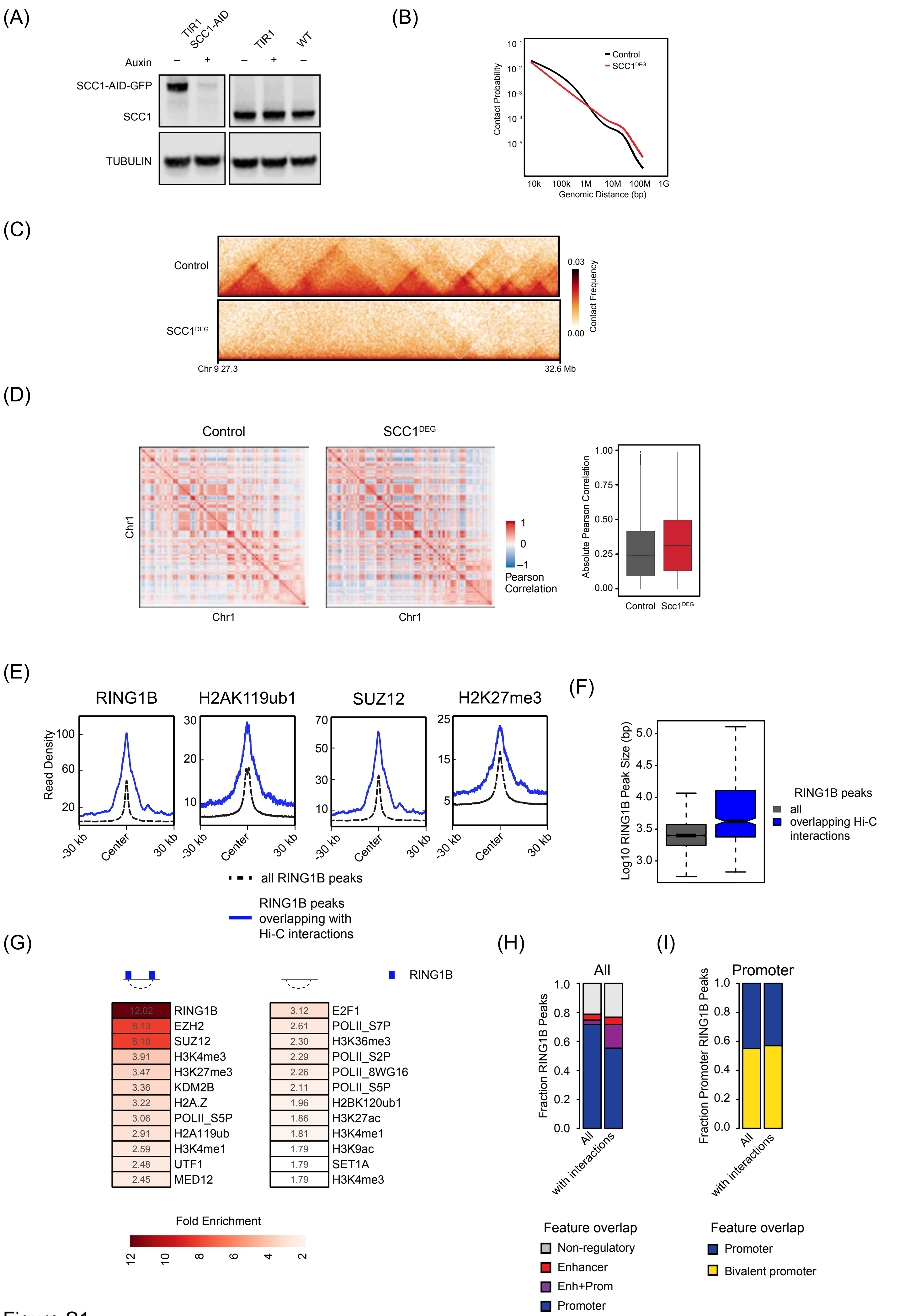
(A)
TIR1 SCC1-AID
TIR1
WT
Auxin
– + – + –
SCC1-AID-GFP
SCC1
TUBULIN
(B)
Contact Probability
Genomic Distance (bp)
10k 100k 1M 10M 100M 1G
Control
SCC1DEG
(C)
Control
SCC1DEG
0.03
0.00
Contact Frequency
Chr 9 27.3
32.6 Mb
(D)
Control
SCC1DEG
Chr1
Pearson Correlation
1
0
–1
Absolute Pearson Correlation
1.00
0.75
0.50
0.25
0.00
Control
Scc1DEG
(E)
RING1B
H2AK119ub1
SUZ12
H2K27me3
Read Density
-30 kb
Center
30 kb
all RING1B peaks
RING1B peaks overlapping with Hi-C interactions
(F)
Log10 RING1B Peak Size (bp)
5.0
4.5
4.0
3.5
3.0
RING1B peaks
all
overlapping Hi-C interactions
(G)
RING1B
12.02 RING1B
8.13 EZH2
8.10 SUZ12
3.91 H3K4me3
3.47 H3K27me3
3.36 KDM2B
3.22 H2A.Z
3.06 POLII_S5P
2.91 H2A119ub
2.59 H3K4me1
2.48 UTF1
2.45 MED12
3.12 E2F1
2.61 POLII_S7P
2.30 H3K36me3
2.29 POLII_S2P
2.26 POLII_8WG16
2.11 POLII_S5P
1.96 H2BK120ub1
1.86 H3K27ac
1.81 H3K4me1
1.79 H3K9ac
1.79 SET1A
1.79 H3K4me3
Fold Enrichment
12 10 8 6 4 2
(H)
All
Fraction RING1B Peaks
All
with interactions
Feature overlap
Non-regulatory
Enhancer
Enh+Prom
Promoter
(I)
Promoter
Fraction Promoter RING1B Peaks
All
with interactions
Feature overlap
Promoter
Bivalent promoter

Figure S1

Supplementary Figure 1 - Characterisation of SCC1^{DEG} ESCs. Related to Figure 1

(A) A representative western blot for SCC1 in the TIR1 and SCC1-mAID-GFP (=SCC1^{DEG}) cell lines ± auxin. A wild type cell line is shown for comparison and tubulin is shown as a loading control.
(B) Genomic distance-dependent contact probability from Hi-C in Control or SCC1^{DEG} cells.
(C) Hi-C in Control and SCC1^{DEG} cells at 10 kb resolution.
(D) Pearson correlation coefficient of chromosome 1 from Control and SCC1^{DEG} at 500 kb resolution (left). Bar plot of the genome-wide absolute Pearson correlation for Control and SCC1^{DEG} (right).
(E) RING1B, H2AK119ub1, SUZ12, and H3K27me3 ChIP-seq signal metaplot at RING1B peaks overlapping with interactions that persist in the absence of cohesin (blue line) or all RING1B peaks (dashed-black line). Datasets used for this figure are indicated in the Supplementary Table S1.
(F) A box plot showing the RING1B peak size at RING1B peaks overlapping interactions that persist in the absence of cohesin (blue right) or all RING1B peaks (grey left).
(G) Enrichment of histone modifications and proteins at paired interaction sites compared to the enrichments at matched random interaction sites for interactions with RING1B peaks at both (left), or at neither (right) of the interacting sites. Fold enrichment is indicated within the heatmaps.
(H) Fraction of genomic features overlapping with all RING1B peaks or only RING1B peaks associated with cohesin-independent interactions.
(I) Fraction of all RING1B peaks or RING1B peaks associated with cohesin-independent interactions that overlap with non-bivalent (promoter) or bivalent promoters.

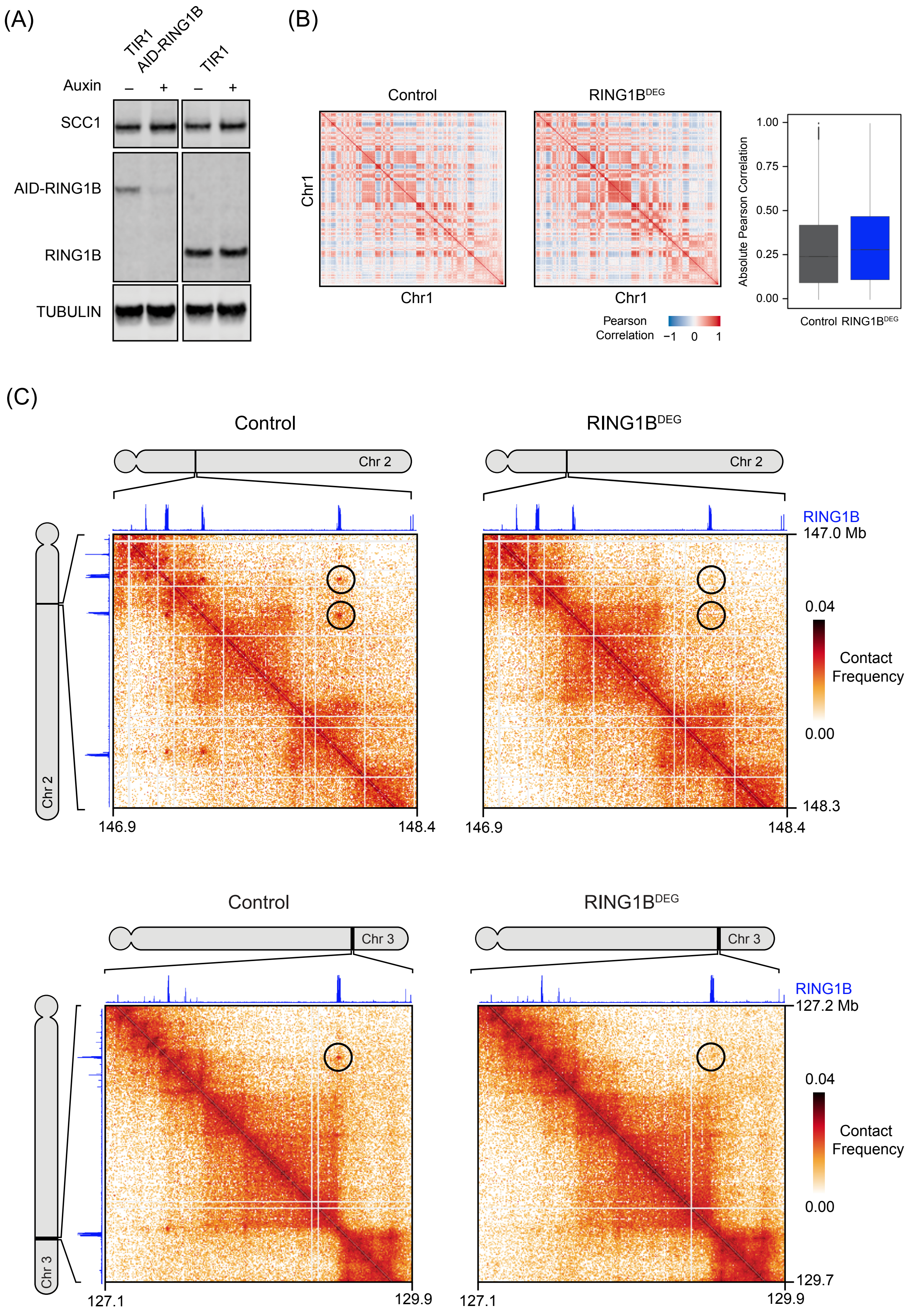

(A)
TIR1
AID-RING1B
TIR1
Auxin
–
+
–
+
SCC1
AID-RING1B
RING1B
TUBULIN
(B)
Control
RING1BDEG
Chr1
Chr1
Chr1
Pearson
Correlation
−1
0
1
Absolute Pearson Correlation
1.00
0.75
0.50
0.25
0.00
Control
RING1BDEG
(C)
Control
RING1BDEG
Chr 2
Chr 2
RING1B
147.0 Mb
0.04
Contact
Frequency
0.00
148.3
146.9
148.4
146.9
148.4
Chr 2
Control
RING1BDEG
Chr 3
Chr 3
RING1B
127.2 Mb
0.04
Contact
Frequency
0.00
129.7
127.1
129.9
127.1
129.9
Chr 3

Figure S2

Supplementary Figure 2 - Characterisation of RING1B^{DEG} ESCs. Related to Figure 2

(A) A representative western blot for SCC1 and RING1B in the Control and AID-RING1B cell lines ± auxin. Tubulin is shown as a loading control.
(B) Pearson correlation coefficient of chromosome 1 from Control and RING1B^{DEG} at 500 kb resolution (left). Bar plot of the genome-wide absolute Pearson correlation for Control and RING1B^{DEG} (right).
(C) Hi-C in Control and RING1B^{DEG} cells at two regions (top at 5 kb resolution and bottom at 10 kb resolution). Black circles indicate at interactions that persist in the absence of cohesin and RING1B ChIP-seq is displayed above and to the left of the matrices.

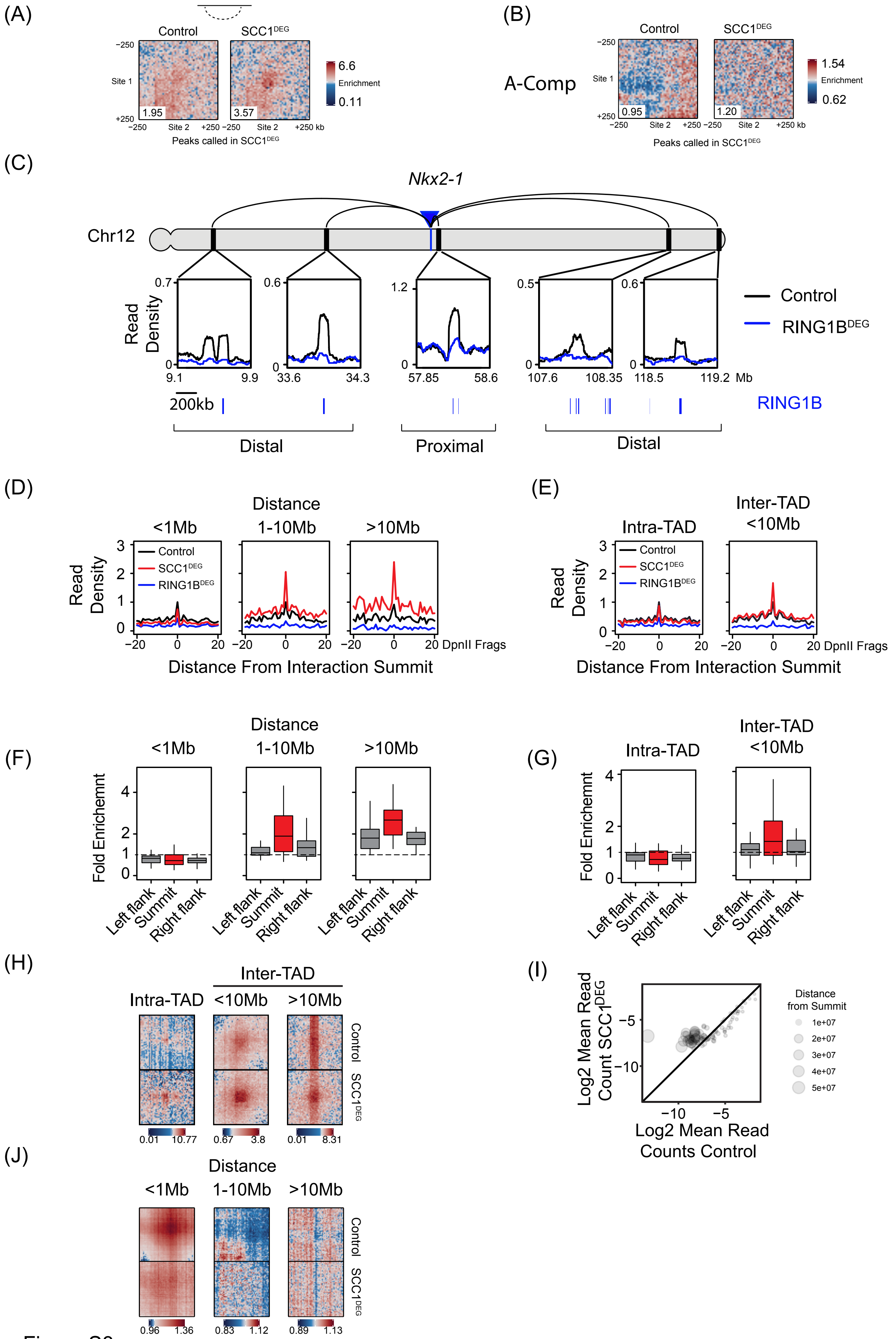

(A)
Control
SCC1DEG
−250
Site 1
+250
1.95
3.57
−250
Site 2
+250 −250
Site 2
+250 kb
6.6
Enrichment
0.11
Peaks called in SCC1DEG
(B)
A-Comp
Control
SCC1DEG
0.95
1.20
1.54
Enrichment
0.62
Peaks called in SCC1DEG
(C)
Nkx2-1
Chr12
Read Density
Control
RING1BDEG
200kb
RING1B
Distal
Proximal
Distal
Mb
(D)
Distance
<1Mb
1-10Mb
>10Mb
Control
SCC1DEG
RING1BDEG
Read Density
20 DpnII Frags
Distance From Interaction Summit
(E)
Intra-TAD
Inter-TAD <10Mb
Distance From Interaction Summit
(F)
Fold Enrichemnt
Left flank
Summit
Right flank
(G)
(H)
Inter-TAD
Intra-TAD
<10Mb
>10Mb
Control
SCC1DEG
0.01 10.77
0.67 3.8
0.01 8.31
(I)
Log2 Mean Read Count SCC1DEG
Log2 Mean Read Counts Control
Distance from Summit
1e+07
2e+07
3e+07
4e+07
5e+07
(J)
Distance
<1Mb
1-10Mb
>10Mb
0.96 1.36
0.83 1.12
0.89 1.13

Figure S3

Supplementary Figure 3 - Cohesin removal strengthens long-range polycomb chromatin domain interactions. Related to Figure 3

(A) Aggregate analysis of Hi-C from Control and SCC1^{DEG} cells at peaks that persist in the absence of cohesin and do not overlap with RING1B at either interacting site (n=49). In each aggregate plot the trimmed mean of the enrichment scores at the centre 50kb of the aggregated matrix is displayed in the bottom left corner as a quantification of enrichment.
(B) Aggregate analysis of Hi-C from Control and SCC1^{DEG} cells using randomly selected 100kb regions within A compartments which are distance and chromosome matched to peaks that persist in the absence of cohesin (n=336). In each aggregate plot the trimmed mean of the enrichment scores at the centre 50kb of the aggregated matrix is displayed in the bottom left corner as a quantification of enrichment.
(C) Capture-C interaction profiles between the *Nkx2-1* promoter and selected proximal and distal RING1B-occupied sites in the Control and RING1B^{DEG} cell lines. RING1B ChIP-seq peaks are shown as blue bars below. The location of the *Nkx2-1* promoter is indicated with a blue arrow/bar and the interactions sites as black bars on the chromosome. Read density corresponds to normalised reads in the capture averaged across 250 DpnII restriction fragments.
(D) Aggregate Capture-C signal in the Control, SCC1^{DEG} and RING1B^{DEG} cells at interaction sites segregated based on distance from the capture site. Only interactions between polycomb target gene promoters and RING1B occupied sites present in SCC1^{DEG} are shown. Read density was normalised to Control signal at the summit and the x-axis illustrates the distance from the interaction site in DpnII fragments.
(E) As in (D) but interactions are segregated based on intra- or inter-TAD location.
(F) Fold enrichment of Capture-C signal in SCC1^{DEG} over the Control cell line for signal in the peak summit (red) and flanks (grey), segregated based on distance as in (D). The left flank corresponds to a promoter-distal region, 80-100 DpnII fragments away from the summit. The right flank corresponds to a promoter-proximal region, 80-100 DpnII fragments away from the summit. Enrichment in the flanks shows average enrichment across 20 DpnII fragments. Boxplots show median and the interquartile range. Dotted line: fold enrichment of 1 (= no enrichment).
(G) As in (F) but segregated based on intra- or inter-TAD location.
(H) Aggregate analysis of Hi-C from Control and SCC1^{DEG} cells at interactions that persist in the absence of cohesin. Interactions are divided into those that occur between loci within a TAD (intra-TAD) and between loci that are in different TADs either separated by less than or more than 10Mb.
(I) Mean normalised read Capture-C counts in the Control and SCC1^{DEG} cell lines for interactions between polycomb target gene promoters and RING1B occupied sites. Circle size indicates the distance between promoter and interactions sites.
(J) Aggregate peak analysis of Hi-C for interactions between repressed but not polycomb occupied TSSs in mESCs. Interactions were segregated based on distance as indicated above the aggregate plots.

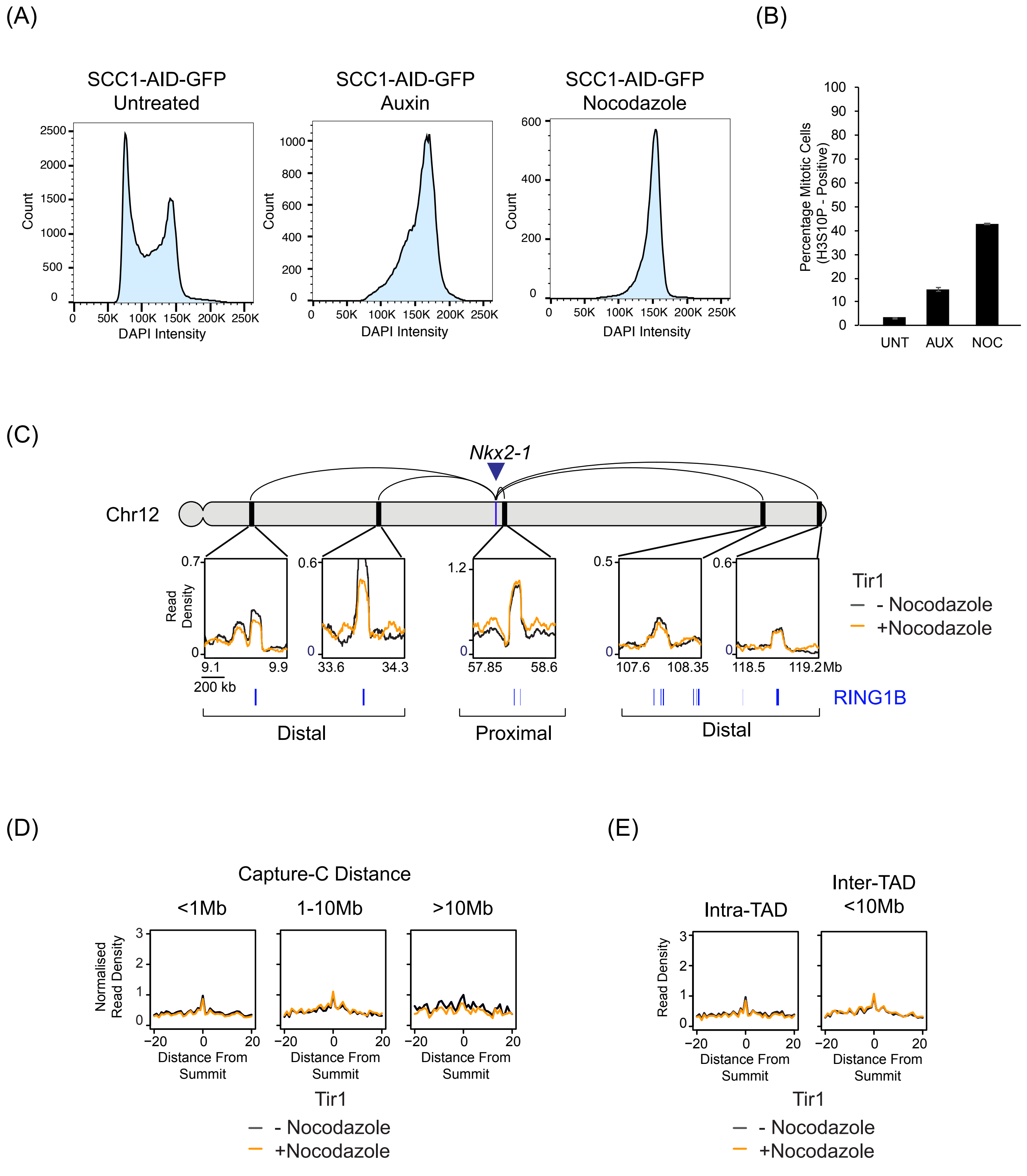
(A)
SCC1-AID-GFP
Untreated
SCC1-AID-GFP
Auxin
SCC1-AID-GFP
Nocodazole
Count
DAPI Intensity
0
50K
100K
150K
200K
250K
(B)
Percentage Mitotic Cells
(H3S10P - Positive)
UNT
AUX
NOC
(C)
Nkx2-1
Chr12
Read
Density
9.1
9.9
33.6
34.3
57.85
58.6
107.6
108.35
118.5
119.2Mb
200 kb
RING1B
Distal
Proximal
Distal
Tir1
- Nocodazole
+Nocodazole
(D)
Capture-C Distance
<1Mb
1-10Mb
>10Mb
Normalised
Read Density
Distance From
Summit
Tir1
- Nocodazole
+Nocodazole
(E)
Intra-TAD
Inter-TAD
<10Mb
Read Density
Distance From
Summit
Tir1
- Nocodazole
+Nocodazole

Figure S4

Supplementary Figure 4 – Increased interaction between polycomb chromatin domains is not due to altered cell cycle distribution. Related to Figure 3

(A) Cell cycle distribution of SCC1-AID-GFP cells either untreated (left), or treated with Auxin (middle) or Nocodazole (right) for six hours.
(B) Percentage mitotic cells as accessed by histone H3 serine 10 phosphorylation (H3S10P) positive cells in FACS for conditions indicated in (A)(n=3).
(C) Capture-C interaction profiles between the *Nkx2-1* promoter and selected proximal and distal RING1B-occupied sites in untreated (black) or Nocodazole treated (orange) Tir1 ESC line. RING1B ChIP-seq peaks are shown as blue bars below. The location of the *Nkx2-1* promoter is indicated with a blue arrow/bar and the interactions sites as black bars on the chromosome. Read density corresponds to normalised reads in the capture averaged across 250 DpnII restriction fragments.
(D) Aggregate Capture-C signal in untreated (black) or Nocodazole treated (orange) Tir1 ESC line at interaction sites segregated based on distance from the capture site. Only interactions between polycomb target gene promoters and RING1B occupied sites present in $SCC1^{DEG}$ are shown. Read density was normalised to Control signal at the summit and the x-axis illustrates the distance from the interaction site in DpnII fragments.
(E) As in (D) but segregated based on intra- or inter-TAD location.

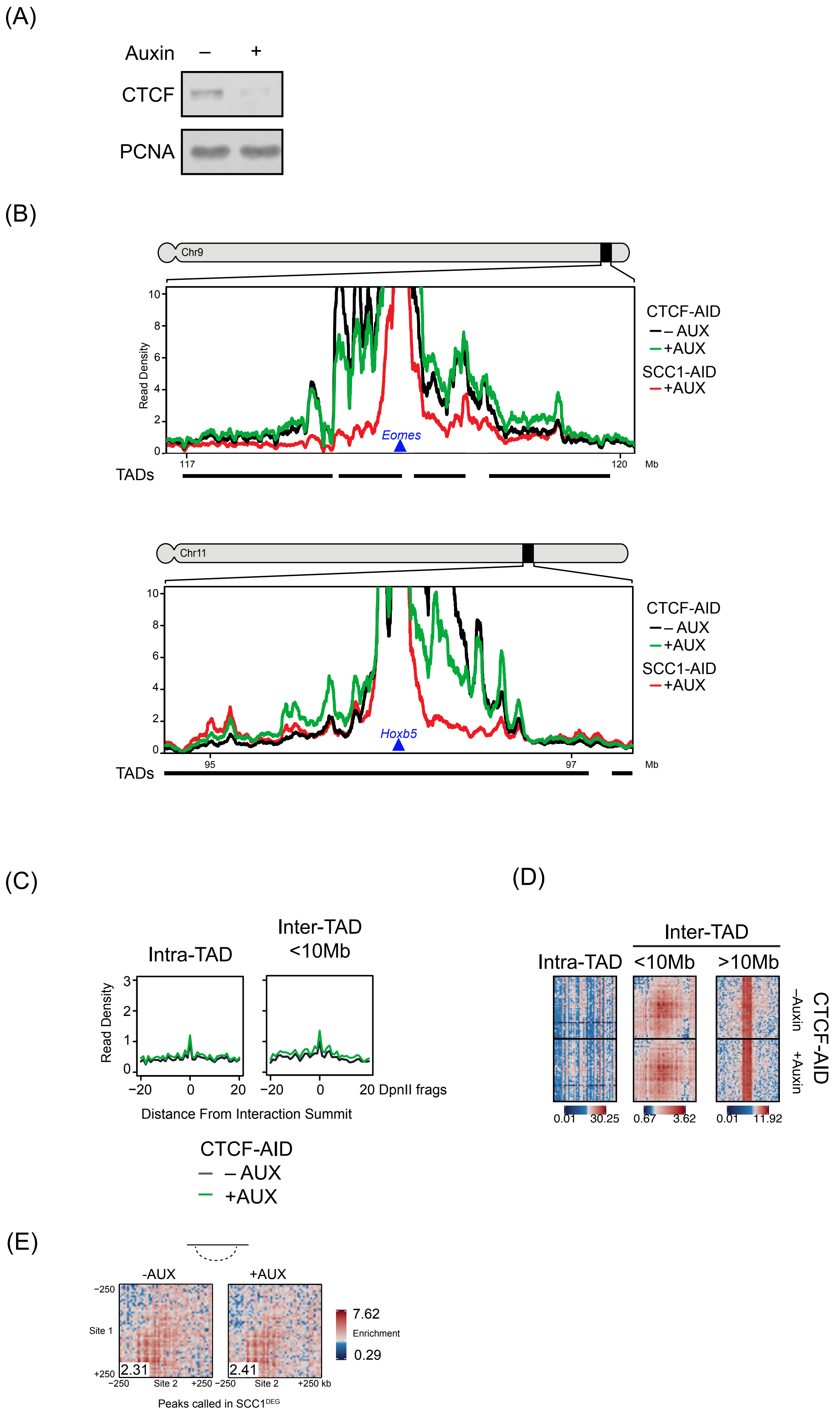
(A)
Auxin – +
CTCF
PCNA
(B)
Chr9
Read Density
CTCF-AID
– AUX
+AUX
SCC1-AID
+AUX
Eomes
117
120
Mb
TADs
Chr11
CTCF-AID
– AUX
+AUX
SCC1-AID
+AUX
Hoxb5
95
97
Mb
TADs
(C)
Intra-TAD
Inter-TAD <10Mb
Read Density
−20 0 20 −20 0 20 DpnII frags
Distance From Interaction Summit
CTCF-AID
– AUX
+AUX
(D)
Inter-TAD
Intra-TAD
<10Mb
>10Mb
−Auxin
+Auxin
CTCF-AID
0.01 30.25
0.67 3.62
0.01 11.92
(E)
-AUX
+AUX
−250
Site 1
+250
2.31
2.41
−250 Site 2 +250 −250 Site 2 +250 kb
7.62
Enrichment
0.29
Peaks called in SCC1DEG

Figure S5

Supplementary Figure 5 - Cohesin counteracts polycomb chromatin domain interactions independently of CTCF and insulation. Related to Figure 5

(A) A representative western blot for CTCF in the CTCF-AID cell lines ± auxin. PCNA is shown as a loading control.
(B) Capture-C signal around *Eomes* and *Hoxb5* gene promoters. Shown are normalized read densities for CTCF-AID ± AUX (green and black, respectively) and as comparison for $SCC1^{DEG}$ (red). Read density corresponds to normalised reads in the capture averaged across 80 DpnII restriction fragments. View point is indicated as a blue triangle. TAD boundaries are shown below.
(C) Aggregate Capture-C signal in the CTCF-AID-GFP cells ± auxin at interactions that persist in the absence of cohesin segregated based on TAD identity. Only interactions between polycomb target gene promoters and RING1B occupied sites present in $SCC1^{DEG}$ are shown. Read density was normalised to signal at the summit in CTCF-AID-GFP cells without auxin and the x-axis illustrates the distance from the interaction site in DpnII fragments.
(D) Aggregate analysis of Hi-C from CTCF-AID cells ± auxin at peaks that persist in the absence of cohesin (n=336). Interactions are divided into those that occur between loci within a TAD (intra-TAD), between loci that are in different TAD and are separated by less than 10 Mb (inter TAD >10 Mb) and between loci that are in different TADs and are separated by less than 10 Mb (inter TAD >10 Mb).
(E) Aggregate analysis of Hi-C from CTCF-AID ± auxin cells (Nora et al., 2017) at peaks that persist in the absence of cohesin and do not overlap with RING1B at either interacting site (n=49).

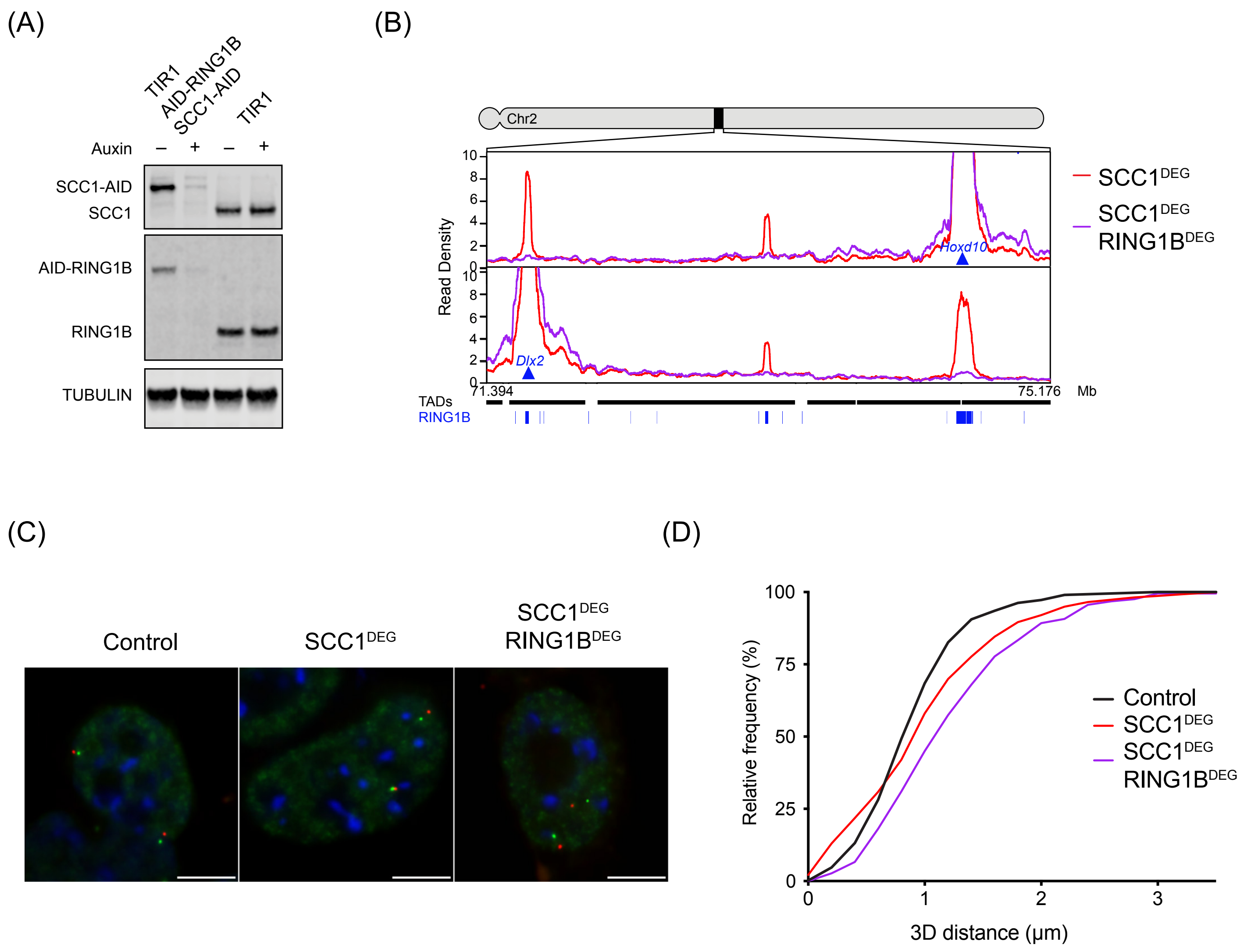

(A)
TIR1
AID-RING1B
SCC1-AID
TIR1
Auxin
–
+
–
+
SCC1-AID
SCC1
AID-RING1B
RING1B
TUBULIN
(B)
Chr2
Read Density
SCC1DEG
SCC1DEG
RING1BDEG
Hoxd10
Dlx2
71.394
75.176
Mb
TADs
RING1B
(C)
Control
SCC1DEG
SCC1DEG
RING1BDEG
(D)
Relative frequency (%)
3D distance (μm)
Control
SCC1DEG
SCC1DEG
RING1BDEG

Figure S6

Supplementary Figure 6 - Polycomb chromatin domain interactions are disrupted by cohesin. Related to Figure 6.

(A) A representative western blot for SCC1 and RING1B in the TIR1 and AID-RING1B SCC1-mAID-GFP cell lines ± auxin. Tubulin is shown as a loading control.
(B) Capture-C interaction profiles from *Hoxd10* (top) and *Dlx2* (bottom) viewpoints in Control and SCC1^{DEG} RING1B^{DEG} cell lines. RING1B ChIP-Seq peaks are displayed as blue bars and TAD intervals are as black bars.
(C) Representative *Hoxd10* (red) *Dlx2* (green) RASER-FISH images from the indicated cell lines. Scale bar = 5 µm.
(D) Cumulative frequency distribution of 3D distance measures between *Hoxd10* and *Dlx2* in the indicated cells lines. Measurements as in Figure 5C.

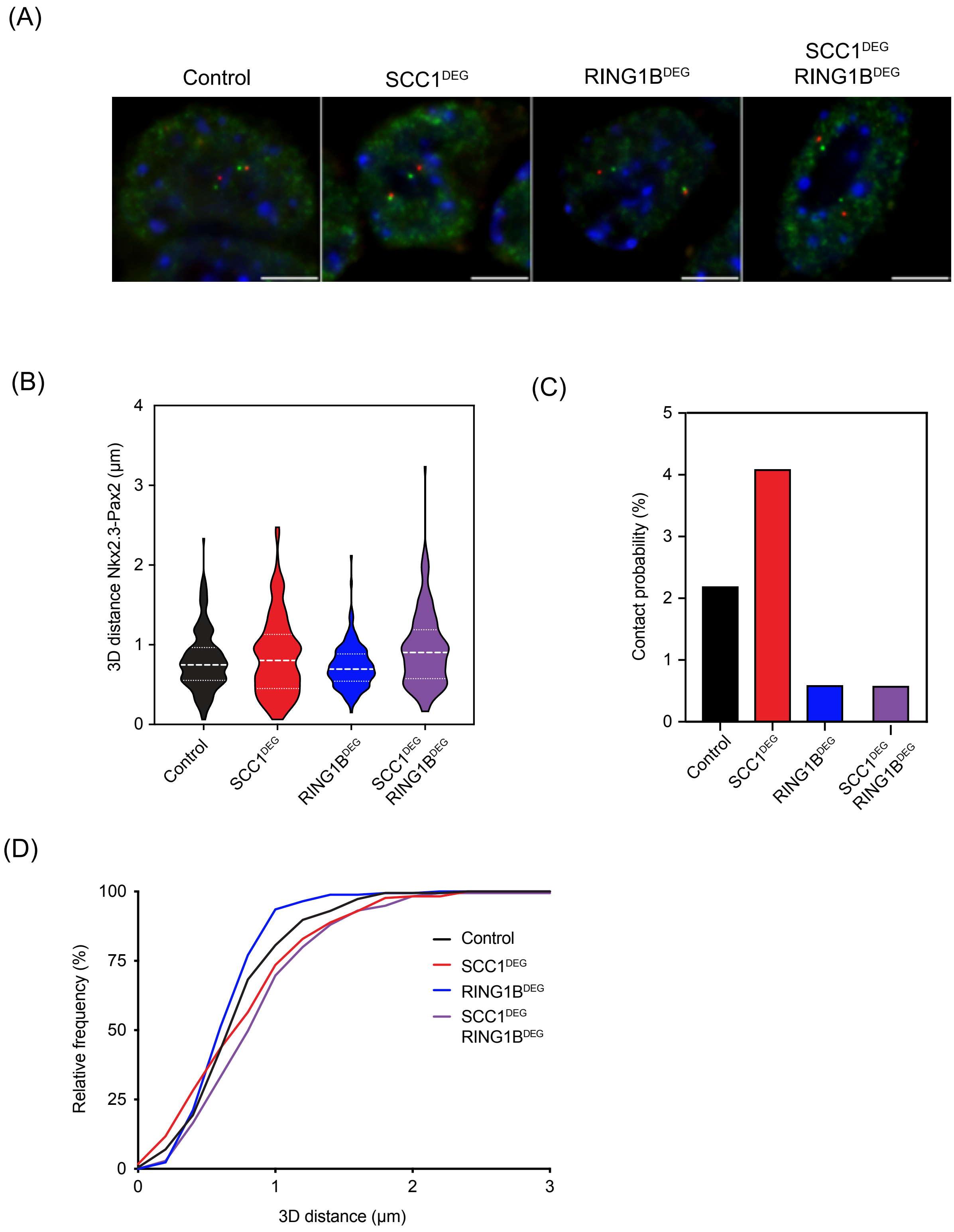
(A)
Control
SCC1DEG
RING1BDEG
SCC1DEG
RING1BDEG
(B)
3D distance Nkx2.3-Pax2 (µm)
0
1
2
3
4
Control
SCC1DEG
RING1BDEG
SCC1DEG
RING1BDEG
(C)
Contact probability (%)
0
1
2
3
4
5
Control
SCC1DEG
RING1BDEG
SCC1DEG
RING1BDEG
(D)
Relative frequency (%)
0
25
50
75
100
0
1
2
3
3D distance (µm)
Control
SCC1DEG
RING1BDEG
SCC1DEG
RING1BDEG

Figure S7

Supplementary Figure 7 - Polycomb chromatin domain interactions are disrupted by cohesin. Related to Figure 6

(A) Representative RASER-FISH images illustrating the *Nkx2-3* (green) and *Pax2* (red) loci. Scale bar is 5 µm.
(B) Violin plots showing 3D distance measurements between *Nkx2-3* and *Pax2* in the indicated cells lines. The dashed lines show the median and interquartile range of between n=170-186 alleles for each cell line.
(C) Absolute contact probabilities showing the percent of signals judged as colocalised from observations in (B) (see Methods).
(D) Cumulative frequency distribution of 3D distance measures between *Nkx2-3* and *Pax2* in the indicated cells lines. Measurements as in (B).

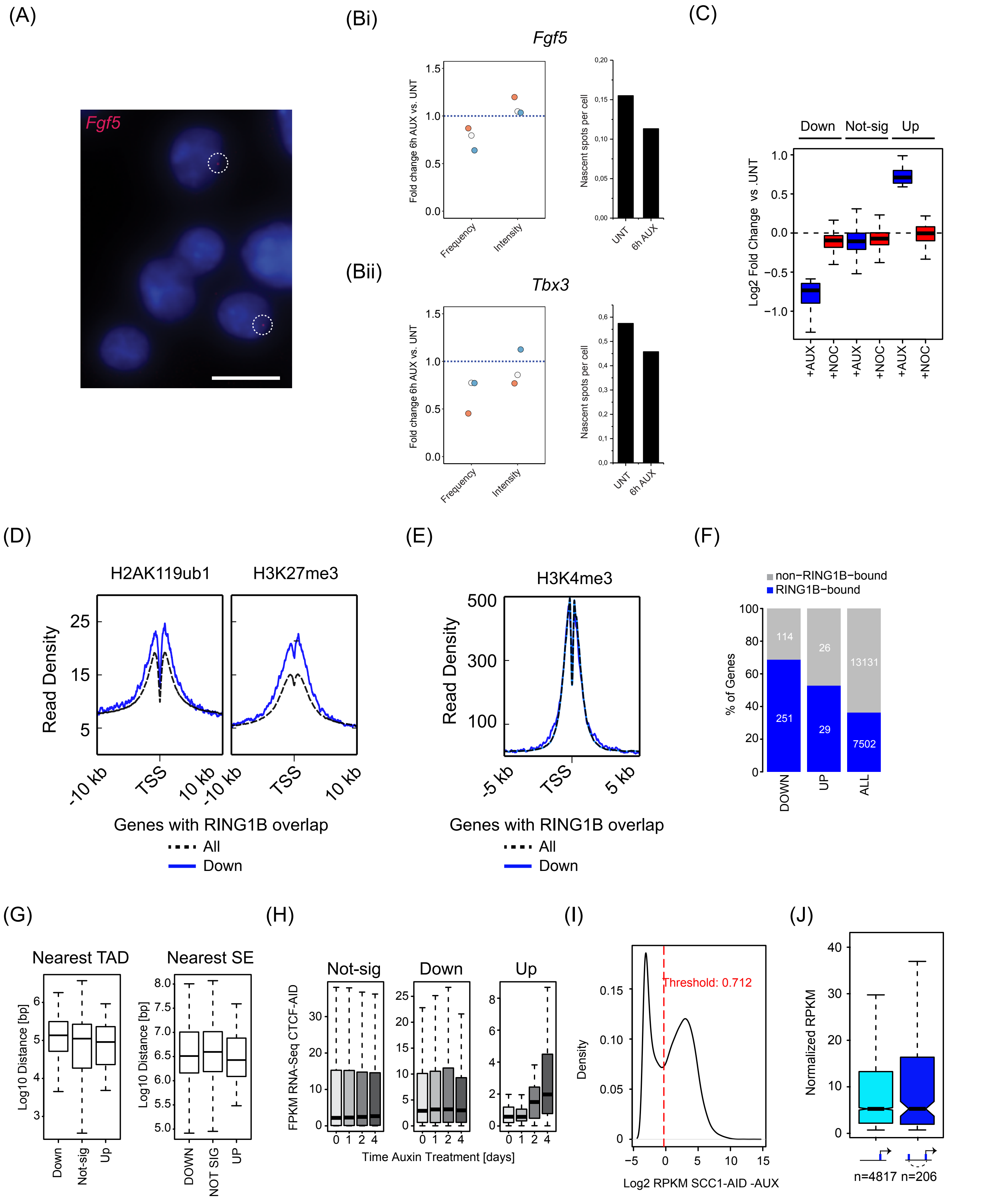
(A)
Fgf5
(Bi)
Fgf5
Fold change 6h AUX vs. UNT
Frequency
Intensity
Nascent spots per cell
UNT
6h AUX
(Bii)
Tbx3
(C)
Down
Not-sig
Up
Log2 Fold Change vs .UNT
+AUX
+NOC
(D)
H2AK119ub1
H3K27me3
Read Density
-10 kb
TSS
10 kb
Genes with RING1B overlap
All
Down
(E)
H3K4me3
-5 kb
5 kb
(F)
non−RING1B−bound
RING1B−bound
% of Genes
114
251
26
29
13131
7502
DOWN
UP
ALL
(G)
Nearest TAD
Nearest SE
Log10 Distance [bp]
Down
Not-sig
Up
DOWN
NOT SIG
UP
(H)
Not-sig
Down
Up
FPKM RNA-Seq CTCF-AID
Time Auxin Treatment [days]
(I)
Threshold: 0.712
Density
Log2 RPKM SCC1-AID -AUX
(J)
Normalized RPKM
n=4817
n=206

Figure S8

Supplementary Figure 8 - Increased polycomb chromatin domain association in the absence of cohesin suppresses gene expression. Related to Figure 7

A) Fluorescence microscopy image of ESCs labelled with DAPI (blue) and nascent RNA-FISH for *Fgf5* gene (red). Scale bar corresponds to 10 µm. Dashed circles indicate active *Fgf5* alleles positive for RNA-FISH signal.
(B) Frequency and intensity of signals depicted in (A) highlighting transcriptionally active alleles have been measured for *Fgf5* and *Tbx3* and are presented in (Bi) and (Bii), respectively. (Left) A comparison of the frequency and intensity of RNA-FISH spots following depletion of cohesin. The individually coloured dots correspond to triplicate experiments. (Right) Bar-plots indicate the absolute frequency of active alleles in the cell population prior to and after cohesin removal for biological triplicates. Approximately 1000 cells have been measured in each biological replicate and condition.
(C) Box plots illustrating log2 fold gene expression changes from cRNA-seq analysis of genes that are down, not significantly, or up-regulated following depletion of SCC1. The expression changes in the $SCC1^{DEG}$ cells are indicated as +AUX (blue) and expression changes in cells treated with nocodazole are indicated as +NOC (red).
(D) H2AK119ub1 and H3K27me3 ChIP-seq metaplots at the promoters of all genes that overlap with RING1B peaks (dashed-black line) or RING1B peaks that are associated with a reduction (blue line) in gene expression.
(E) As (D) but for H3K4me3.
(F) RING1B binding (+/- 1kb from the TSS, blue bars) at gene promoters that show reductions in gene expression following cohesin removal (left) compared to genes that show an increase in gene expression (middle) and all genes (right).
(G) Log10 distance in base pairs to the nearest TAD border (left) or nearest super-enhancer in a different TAD (right) for genes that are downregulated, not significantly affected, or upregulated upon cohesin depletion.
(H) Expression of upregulated (right), downregulated (middle) or not significantly affected (left) in CTCF-AID cell line (Nora et al., 2017). Duration of auxin treatment in days is indicated below the boxplots.
(I) Density distribution of Log2 transformed RPKM of transcripts in untreated Scc1-mAID cells. Note the bimodal distribution. Red line marks the threshold (RPKM=0.712) to distinguish expressed from unexpressed genes.
(J) Boxplots show transcriptional levels in untreated SCC1-mAID-GFP. Transcription is shown for expressed genes with RING1B-ocupied promoters that either have no detectable Hi-C interactions in SCC1-AID cells treated with auxin (light blue), or interact with other sites that are RING1B occupied (dark blue). Numbers of genes in each group are indicated below the x-axis.

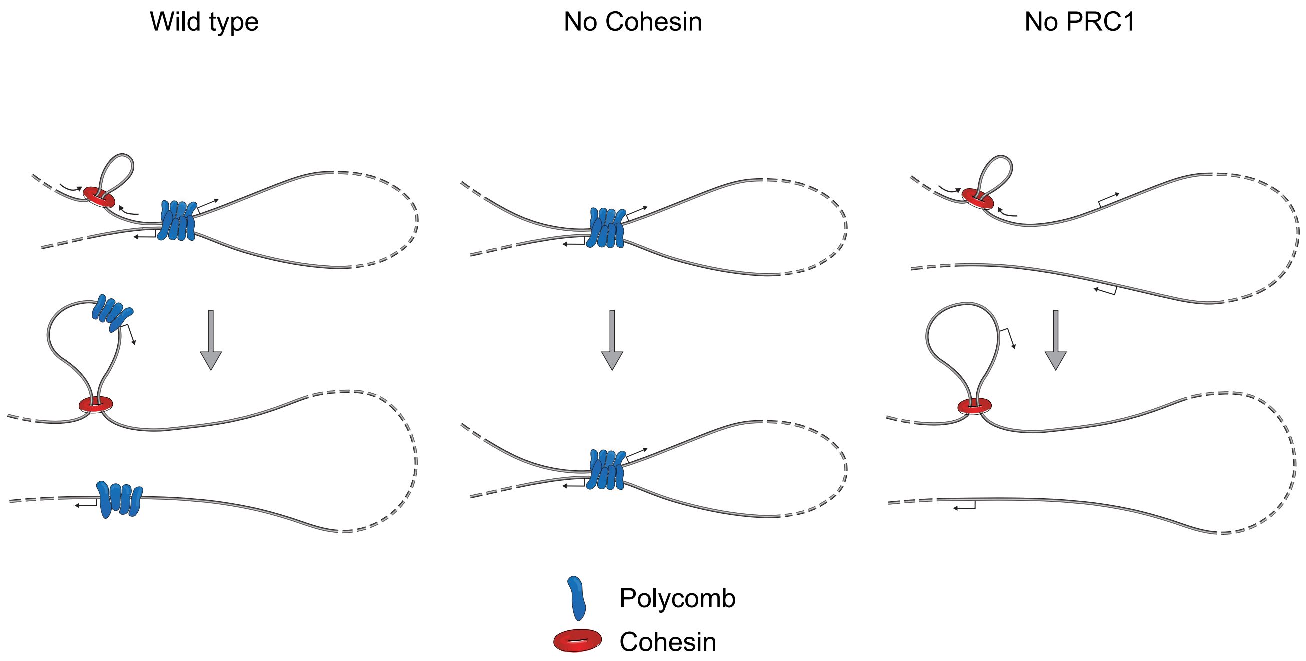

Figure S9

Supplementary Figure 9 – A model for disruption of polycomb chromatin domain interactions by cohesin and loop extrusion. Related to Figures 1-7

Polycomb chromatin domains (blue) can interact even when separated by large distances on the chromosome. Cohesin (red circle) can load onto DNA and has been proposed to extrude chromatin. As loop extrusion proceeds it will encounter one of the two interacting polycomb chromatin domains. We propose that the manner in which chromatin is extruded through cohesin could lead to the individualisation of these two previously interacting polycomb chromatin domains and explain the observed effect that cohesin removal has on polycomb chromatin domain interaction in our chromosome conformation capture and single-cell imaging experiments. A key prediction of this model would be that cohesin loading and extrusion near either interacting polycomb chromatin domain would lead to the observed effect.

Supplementary Table 1. Datasets analysed in this study. Related to Figures 1, 4 and 5

Name	Reference	GEO
CDK8 ChIP-Seq	Dimitrova_and_Klose_Elife_2018	GSE98756
RING1B ChIP-Seq	Blackledge_and_Klose_Cell_2014	GSE55698
SUZ12 ChIP-Seq	Blackledge_and_Klose_Cell_2014	GSE55698
H3K27me3 ChIP-Seq	Blackledge_and_Klose_Cell_2014	GSE55698
EZH2 ChIP-Seq	Blackledge_and_Klose_Cell_2014	GSE55698
FBXL19-FS2 ChIP-Seq	Dimitrova_and_Klose_Elife_2018	GSE98756
H3K4me1 ChIP-Seq	Whyte_and_Young_Nature_2012	GSE27844
CTCF ChIP-Seq	mouse ENCODE (LICR)	
ESRRB ChIP-Seq	Chen_and_Ng_Cell_2008	GSE11431
KLF4 ChIP-Seq	Chen_and_Ng_Cell_2008	GSE11431
NANOG ChIP-Seq	Whyte_and_Young_Cell_2013	GSE44288
OCT4 ChIP-Seq	Whyte_and_Young_Cell_2013	GSE44288
SOX2 ChIP-Seq	Whyte_and_Young_Cell_2013	GSE44288
YY1 ChIP-Seq	Sigova_and_Young_Science_2015	GSE68195
p300 ChIP-Seq	mouse ENCODE	
CDK8 ChIP-Seq	Whyte_and_Young_Cell_2013	GSE44288
CDK9 ChIP-Seq	Whyte_and_Young_Cell_2013	GSE44288
MED1 ChIP-Seq	Whyte_and_Young_Cell_2013	GSE44288
MED12 ChIP-Seq	Whyte_and_Young_Cell_2013	GSE44288
MED1 ChIP-Seq	Sun_and_Carey_MolCell_2018	GSE115340
MED12 ChIP-Seq	Sun_and_Carey_MolCell_2018	GSE115340
H3K27ac ChIP-Seq	Whyte_and_Young_Nature_2012	GSE27844
H3K36me3 ChIP-Seq	Brookes_and_Pombo_CellStemCell_2012	GSE34520
H3K9ac ChIP-Seq	mouse ENCODE	
H3K4me3 ChIP-Seq	Brown_and_Klose_CellReports_2017	GSE93538
SET1A.T7 ChIP-Seq	Brown_and_Klose_CellReports_2017	GSE93538
MLL2.N.GFP ChIP-Seq	Denissov_and_Stewart_Development_2014	GSE52071
CFP1 ChIP-Seq	Brown_and_Klose_CellReports_2017	GSE93538
POLII_S7P ChIP-Seq	Brookes_and_Pombo_CellStemCell_2012	GSE34520
POLII_S5P ChIP-Seq	Brookes_and_Pombo_CellStemCell_2012	GSE34520
POLII_S2P ChiP-Seq	Brookes_and_Pombo_CellStemCell_2012	GSE34520
POLII_8WG16 ChIP-Seq	Brookes_and_Pombo_CellStemCell_2012	GSE34520
TCF3 ChIP-Seq	Marson_and_Young_Cell_2008	GSE11724
NeuroD1 ChIP-Seq	Pataskar_and_Tiwari_EmboJ_2016	GSE65072
TBX3 ChIP-Seq	Kartikasari_and_Bhushan_EmboJ_2013	GSE44764
ZFP143 ChIP-Seq	Ngondo-Mbongo_and_Carbon_NAR_2013	GSE39263
GABPA ChIP-Seq	Savic_and_Myers_GenomeResearch_2015	GSE72082
FOXD1.FLAG ChIP-Seq	Respuela_and_RadaIglesias_CellStemCell_2016	GSE70547
FOXD1.HA ChIP-Seq	Respuela_and_RadaIglesias_CellStemCell_2016	GSE70547
POU3F1 ChIP-Seq	Song_and_Jing_GenomData_2015	GSE69865
Nur77 ChIP-Seq	Terranova_and_Stachowiak_PlosOne_2015	GSE65698
TEX10.FLAG ChIP-Seq	Ding_and_Wang_CellStemCell_2015	GSE66736
FGFR ChIP-Seq	Terranova_and_Stachowiak_PlosOne_2015	GSE65698
RXRa ChIP-Seq	Terranova_and_Stachowiak_PlosOne_2015	GSE65698

PREP1 ChIP-Seq	Laurent_and_Penkov_PlosOne_2015	GSE63282
UTF1.Biotin ChIP-Seq	Galonska_and_Meissner_StemCellReports_2014	GSE53768
UTF1 ChIP-Seq	Galonska_and_Meissner_StemCellReports_2014	GSE53768
RONIN ChIP-Seq	Hnisz_and_Young_Cell_2013	GSE51522
NR5A2.HA ChIP-Seq	Heng_and_Ng_CellStemCell_2010	GSE19019
ZFX ChIP-Seq	Chen_and_Ng_Cell_2008	GSE11431
STAT3 ChIP-Seq	Chen_and_Ng_Cell_2008	GSE11431
TCFCP2I1 ChIP-Seq	Chen_and_Ng_Cell_2008	GSE11431
SMAD1 ChIP-Seq	Chen_and_Ng_Cell_2008	GSE11431
nMYC ChIP-Seq	Chen_and_Ng_Cell_2008	GSE11431
E2F1 ChIP-Seq	Chen_and_Ng_Cell_2008	GSE11431
cMYC ChIP-Seq	Chen_and_Ng_Cell_2008	GSE11431
REST ChIP-Seq	Whyte_and_Young_Cell_2013	GSE44288
MAX ChIP-Seq	Krepelova_and_Oliviero_PlosOne_2014	GSE48175
H3K9me3 ChIP-Seq	BulutKarslioglu_and_Jenuwein_MolCell_2014	GSE57092
H2AUb1 ChIP-Seq	Brookes_and_Pombo_CellStemCell_2012	GSE34520
H2A.Z ChIP-Seq	Surface_and_Boyer_CellRep_2016	GSE53208
BRG1.TAP ChIP-Seq	Dieuleveult_and_Gerard_Nature_2016	GSE64825
CHD1.TAP ChIP-Seq	Dieuleveult_and_Gerard_Nature_2016	GSE64825
CHD2.TAP ChIP-Seq	Dieuleveult_and_Gerard_Nature_2016	GSE64825
CHD4.TAP ChIP-Seq	Dieuleveult_and_Gerard_Nature_2016	GSE64825
CHD6.TAP ChIP-Seq	Dieuleveult_and_Gerard_Nature_2016	GSE64825
CHD8.TAP ChIP-Seq	Dieuleveult_and_Gerard_Nature_2016	GSE64825
CHD9.TAP ChIP-Seq	Dieuleveult_and_Gerard_Nature_2016	GSE64825
EP400.TAP ChIP-Seq	Dieuleveult_and_Gerard_Nature_2016	GSE64825
FAIRE-Seq	Dieuleveult_and_Gerard_Nature_2016	GSE64825
INO80 ChIP-Seq	Wang_and_Hu_CellStemCell_2014	GSE49137
MNAse-Seq	West_and_Kingston_NatComm_2014	GSE59064
KDM2B ChIP-Seq	Blackledge_and_Klose_Cell_2014	GSE55698
HP1.GFP ChIP-Seq	BulutKarslioglu_and_Jenuwein_MolCell_2014	GSE57092
uH2A ChIP-Seq	Fursova_and_Klose_MolCell_2019	GSE119620
E14_Scc1-AID_AUX_RING1B	This study	
E14_Tir1_AUX_RING1B	This study	
CTCF-AID +/- AUX Hi-C	Nora_and_Bruneau_2017	GSE98671
H3K27me3 ChIP-Seq, HCT116	Rao_and_Lieberman-Aiden_2017	GSM2809625
EZH2 ChIP-Seq, HCT116	ENCODE Consortium, 2012	GSM3498250
H3K27me3 ChIP-Seq, HAP1	Campagne_and_Margueron_2019	GSE110143
H2AK119ub ChIP-Seq, HAP1	Campagne_and_Margueron_2019	GSE110143
H3K27me3 ChIP-Seq, HeLa	ENCODE Consortium, 2012	GSM733696
EZH2 ChIP-Seq , HeLa	ENCODE Consortium, 2012	GSM1003520
H3K27me3 ChIP-Seq, adult mouse liver	Ren lab, ENCODE	GSM769034
HCT116 Hi-C	Rao_and_Lieberman-Aiden_2017	GSE104333

Mouse hepatocytes Hi-C	Schwarzer_and_Spitz_2017	GSE93431
HeLa Hi-C	Wutz_and_Peters_2017	GSE102884
HAP1 Hi-C	Haarhuis_and_Rowland_2017	GSE95014
