# Supplementary material for: Cohesin Disrupts Polycomb-Dependent Chromosome Interactions in Embryonic Stem Cells
Source: Cell Rep. 2020 Jan 21;30(3):820–835.e10. doi: 10.1016/j.celrep.2019.12.057 (PMC6988126; doi:10.1016/j.celrep.2019.12.057)
Supplement: Document S2. Article plus Supplemental Information [file mmc3.pdf]

Cell Reports

Article

Cohesin Disrupts Polycomb-Dependent Chromosome Interactions in Embryonic Stem Cells

Graphical Abstract

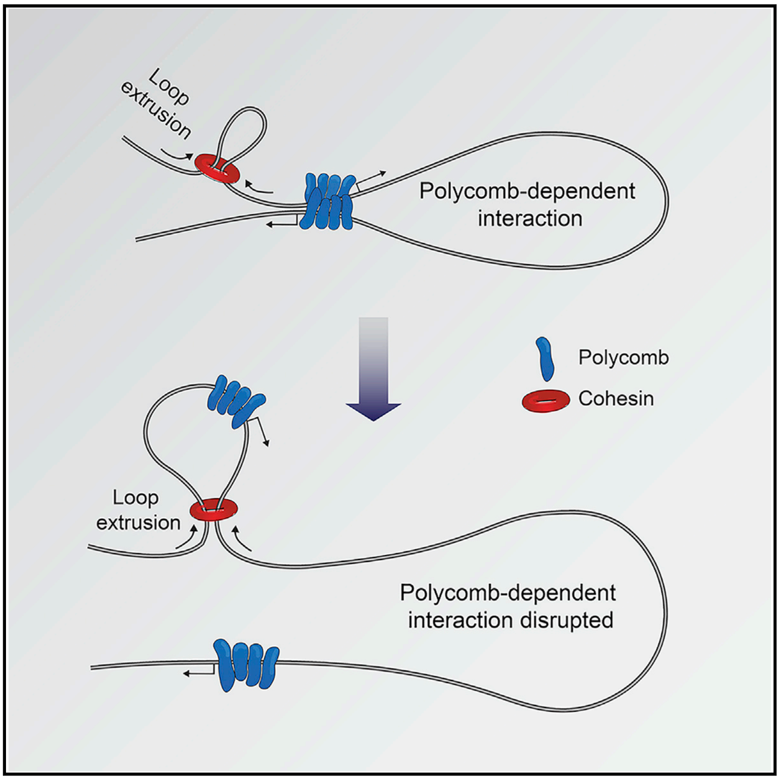

Highlights

- Interaction between polycomb target genes in ESCs occurs independently of cohesin
- Loop extrusion by cohesin disrupts interactions between polycomb target genes
- Cohesin removal enhances repression at polycomb target genes with increased interactions

Authors

James D.P. Rhodes, Angelika Feldmann, Benjamín Hernández-Rodríguez, ..., Veronica J. Buckle, Juan M. Vaquerizas, Robert J. Klose

Correspondence

jmv@mpi-muenster.mpg.de (J.M.V.), rob.klose@bioch.ox.ac.uk (R.J.K.)

In Brief

Using Hi-C, Capture-C, and DNA-FISH, Rhodes et al. discover that interactions between polycomb target genes occur independently of cohesin in embryonic stem cells. This relies on PRC1, and these interactions are disrupted by cohesin-mediated loop extrusion. Upon removal of cohesin, gene repression is enhanced at polycomb-occupied genes with increased interactions.

Rhodes et al., 2020, Cell Reports *30*, 820–835
January 21, 2020 © 2019 The Author(s).
https://doi.org/10.1016/j.celrep.2019.12.057

CellPress

OPEN ACCESS
CellPress

Cell Reports
Article

Cohesin Disrupts Polycomb-Dependent Chromosome Interactions in Embryonic Stem Cells

James D.P. Rhodes,[1,5] Angelika Feldmann,[1,5] Benjamín Hernández-Rodríguez,[2,5] Noelia Díaz,[2,5] Jill M. Brown,[3] Nadezda A. Fursova,[1] Neil P. Blackledge,[1] Praveen Prathapan,[1] Paula Dobrinic,[1] Miles K. Huseyin,[1] Aleksander Szczurek,[1] Kai Kruse,[2] Kim A. Nasmyth,[1] Veronica J. Buckle,[3] Juan M. Vaquerizas,[2,4,*] and Robert J. Klose[1,6,*]
[1]Department of Biochemistry, University of Oxford, South Parks Road, Oxford OX1 3QU, UK
[2]Max Planck Institute for Molecular Biomedicine, Roentgenstrasse 20, 48149 Muenster, Germany
[3]MRC Molecular Haematology Unit, MRC Weatherall Institute of Molecular Medicine, Oxford University, Oxford OX3 9DS, UK
[4]MRC London Institute of Medical Sciences, Institute of Clinical Sciences, Faculty of Medicine, Imperial College London, Du Cane Road, London W12 0NN, UK
[5]These authors contributed equally
[6]Lead Contact
*Correspondence: jmv@mpi-muenster.mpg.de (J.M.V.), rob.klose@bioch.ox.ac.uk (R.J.K.)
https://doi.org/10.1016/j.celrep.2019.12.057

SUMMARY

How chromosome organization is related to genome function remains poorly understood. Cohesin, loop extrusion, and CCCTC-binding factor (CTCF) have been proposed to create topologically associating domains (TADs) to regulate gene expression. Here, we examine chromosome conformation in embryonic stem cells lacking cohesin and find, as in other cell types, that cohesin is required to create TADs and regulate A/B compartmentalization. However, in the absence of cohesin, we identify a series of long-range chromosomal interactions that persist. These correspond to regions of the genome occupied by the polycomb repressive system and are dependent on PRC1. Importantly, we discover that cohesin counteracts these polycomb-dependent interactions, but not interactions between super-enhancers. This disruptive activity is independent of CTCF and insulation and appears to modulate gene repression by the polycomb system. Therefore, we discover that cohesin disrupts polycomb-dependent chromosome interactions to modulate gene expression in embryonic stem cells.

INTRODUCTION

Spatial organization of the genome influences gene transcription and other fundamental DNA-based processes. Recently, genome-wide chromosome conformation capture (Hi-C) has significantly advanced our understanding of chromosomal organization (Rowley and Corces, 2018). This has shown that megabase-sized regions of chromosomes, which have similar transcriptional activity and chromatin modifications, tend to interact preferentially. When these interactions involve active regions of chromosomes, they are referred to as A compartments, and interactions between less active regions are referred to as B compartments (Lieberman-Aiden et al., 2009). At the sub-megabase scale, chromosomes are partitioned into topologically associating domains (TADs), which correspond to contiguous regions of chromatin that interact more frequently than with chromatin outside the domain (Dixon et al., 2012; Nora et al., 2012; Rao et al., 2014). There is increasing evidence that TAD formation occurs through a process called loop extrusion. It has been proposed that cohesin can utilize its ATPase activity to extrude loops of chromatin and that this is limited or terminated by CTCF-occupied insulator DNA elements (Fudenberg et al., 2016; Sanborn et al., 2015). This process is thought to structure and insulate chromosomes, limiting the effects of distal gene regulatory elements to genes within a given TAD. Indeed, alterations in TAD boundaries can lead to perturbed gene expression and human disease (Despang et al., 2019; Kraft et al., 2019; Lupiáñez et al., 2015). Importantly, the function of cohesin in loop extrusion appears to be distinct from its essential and well-characterized role in sister chromatid cohesion (Guacci et al., 1997; Michaelis et al., 1997).

Based on these observations, super-resolution chromosome imaging has been applied to test whether the organizational concepts that emerge from ensemble Hi-C experiments are also evident in single cells (Bintu et al., 2018; Finn et al., 2019; Miron et al., 2019). This has revealed contiguous globular chromosomal structures that are independent of cohesin and loop extrusion and spatially heterogeneous among individual cells. Moreover, single-cell Hi-C experiments indicate that interactions within TADs are infrequent (Flyamer et al., 2017). This suggests that TADs are not static structural entities but result from tendencies to interact, which only become evident when averaged over a population of cells in ensemble Hi-C analysis.

Given that TADs are not fixed structures, fundamental questions remain as to what additional roles cohesin and loop extrusion have in regulating interphase chromosome structure and function. Recent attempts to address these questions have proposed that cohesin regulates interactions between super-enhancers and A/B compartments (Nuebler et al., 2018; Rao et al., 2017; Schwarzer et al., 2017) and helps to actively guide distant enhancers to their target genes in somatic cells (Hadjur et al., 2009). However, to what extent these processes function in different cell types, how they are related to CTCF/TADs, and what role they play in gene regulation remains poorly defined.

 © 2019 The Author(s).
This is an open access article under the CC BY license (http://creativecommons.org/licenses/by/4.0/).

To address these questions, we removed cohesin in mouse embryonic stem cells (ESCs) and examined chromosome interactions by Hi-C. We show that cohesin loss eliminates TADs and enhances A/B compartmentalization as in other cell types (Rao et al., 2017; Schwarzer et al., 2017). However, in the absence of cohesin, we find that a series of long-range high-frequency interactions corresponding to regions of the genome occupied by the polycomb repressive complexes (PRC1 and PRC2) persist. These interactions rely on PRC1 and, interestingly, we discover that interactions between polycomb chromatin domains are strengthened in the absence of cohesin and that this effect is cell-type specific. Using single-cell analysis we demonstrate that cohesin separates polycomb chromatin domains, explaining the effects observed by Hi-C. Removal of CTCF and disruption of TADs does not strengthen these interactions, revealing that cohesin counteracts the association of polycomb chromatin domains through mechanisms that are independent of CTCF and insulation. Moreover, we find that increases in polycomb chromatin domain interactions following cohesin loss appear to have functional consequences on gene expression. Together, these discoveries reveal a role for cohesin in disrupting polycomb-dependent chromosome interactions and gene repression in ESCs.

RESULTS

Cohesin-Independent Chromosomal Interactions Exist in ESCs

We chose to study the loss of cohesin in ESCs, because they are non-transformed, diploid, and have a wealth of existing genomic information characterizing their chromosome structure and chromatin modifications. To do this, we used CRISPR/Cas9-based genome engineering and developed an ESC line in which the cohesin subunit SCC1 (RAD21) could be rapidly removed via an auxin-inducible degron (Natsume et al., 2016; Figures 1A, 1B, and S1A). To examine chromosome interactions in the absence of cohesin, we treated cells with auxin for 6 h to allow the effects of cohesin loss to manifest and compared *in situ* Hi-C (Díaz et al., 2018; Rao et al., 2014) matrices from the SCC1 degron ESCs (SCC1^{DEG}) and control ESCs. Consistent with previous findings (Rao et al., 2017; Schwarzer et al., 2017; Wutz et al., 2017), removal of cohesin caused a complete loss of TADs (Figures S1B and S1C) and modestly enhanced A/B compartmentalization (Figure S1D). However, visual inspection of the Hi-C matrices also revealed numerous interactions that were evident in control cells and persisted in the absence of cohesin (Figure 1C). We then used computational approaches to identify these persistent interactions throughout the genome (Rao et al., 2014) and uncovered 336 sites of high interaction frequency in cohesin-depleted cells. Interestingly, when we examined whether there were any DNA binding factors or chromatin features associated with these interaction sites, there was a strong enrichment of proteins that form polycomb repressive complexes (PRC1 and PRC2) (Figure 1D). This association was further evident when the occupancy of PRC1, PRC2, and their histone modifications were examined at interaction sites (Figures 1E and S1E). The most enriched polycomb protein at these sites was the PRC1 component RING1B. When we examined its occupancy in more detail, we found that 85% (287/336) of interactions had RING1B associated with at least one of the interaction sites and 65% (218/336) had RING1B at both interaction sites. In contrast, interactions that were not associated with RING1B bound sites (49/336 [15%]) were enriched for features associated with actively transcribed genes (Figure S1G) and very few were between super-enhancers (4/336 [1%]). Interestingly, when we examined RING1B-associated interactions, they tended to involve longer than average polycomb chromatin domains that were highly enriched for polycomb proteins, suggesting that the size of the polycomb chromatin domain may contribute to interaction frequency (Figures S1E and S1F). Polycomb chromatin domains can be associated with both promoters and enhancers (Rada-Iglesias et al., 2011). We found that polycomb chromatin domains that persisted in the absence of cohesin were mostly associated with promoters but were not enriched for bivalent chromatin states (Azuara et al., 2006; Bernstein et al., 2006; Figures S1H and S1I). Therefore, removal of cohesin in ESCs leads to strengthening of A/B compartmentalization and loss of TADs, but some strong chromosomal interactions persist, and these tend to correspond to regions of the chromosome occupied by the polycomb system.

Polycomb Mediates Interactions that Persist in the Absence of Cohesin

In ESCs, it is known that polycomb chromatin domains can associate with each other, even over very long distances (Bonev et al., 2017; Denholtz et al., 2013; Joshi et al., 2015; Schoenfelder et al., 2015). To determine whether sites that persisted in the absence of cohesin rely on the polycomb system for their formation, an AID tag was added to RING1B and the closely related paralog, RING1A, was deleted (Figures 2A, 2B, and S2A). We then treated cells for 6 h with auxin to remove RING1B (RING1B^{DEG}) and carried out *in situ* Hi-C. Examination of genomic-distance-dependent contact probabilities and TADs following removal of PRC1 revealed that these features were unaffected (Figures 2C and 2D), with minor increases in A/B compartmentalization (Figure S2B). However, the interactions at sites that persisted in the absence of cohesin were lost from the Hi-C matrices (Figures 2E, 2F, and S2C). Therefore, in ESCs, PRC1 contributes little to A/B compartmentalization and TADs but is responsible for long-range chromosomal interactions that also persist in the absence of cohesin.

Cohesin Removal Strengthens Long-Range Polycomb Chromatin Domain Interactions

In the absence of cohesin, we noticed that the interaction frequency between polycomb chromatin domains often appeared to increase in the Hi-C matrices, suggesting that cohesin may regulate these interactions (Figures 1C and 3A). Indeed, aggregate analysis of the interactions that persist in the absence of cohesin, and that we have shown rely on PRC1 to form, displayed a strong increase in interaction frequency (Figures 3B and S3A). Furthermore, the increase in interaction frequency was more pronounced than the modest increases in compartmentalization that result from cohesin removal (Figure 3B, bottom panel, and Figure S3B). Importantly, this effect did not result from increases in PRC1 occupancy, as RING1B binding was similar, or even

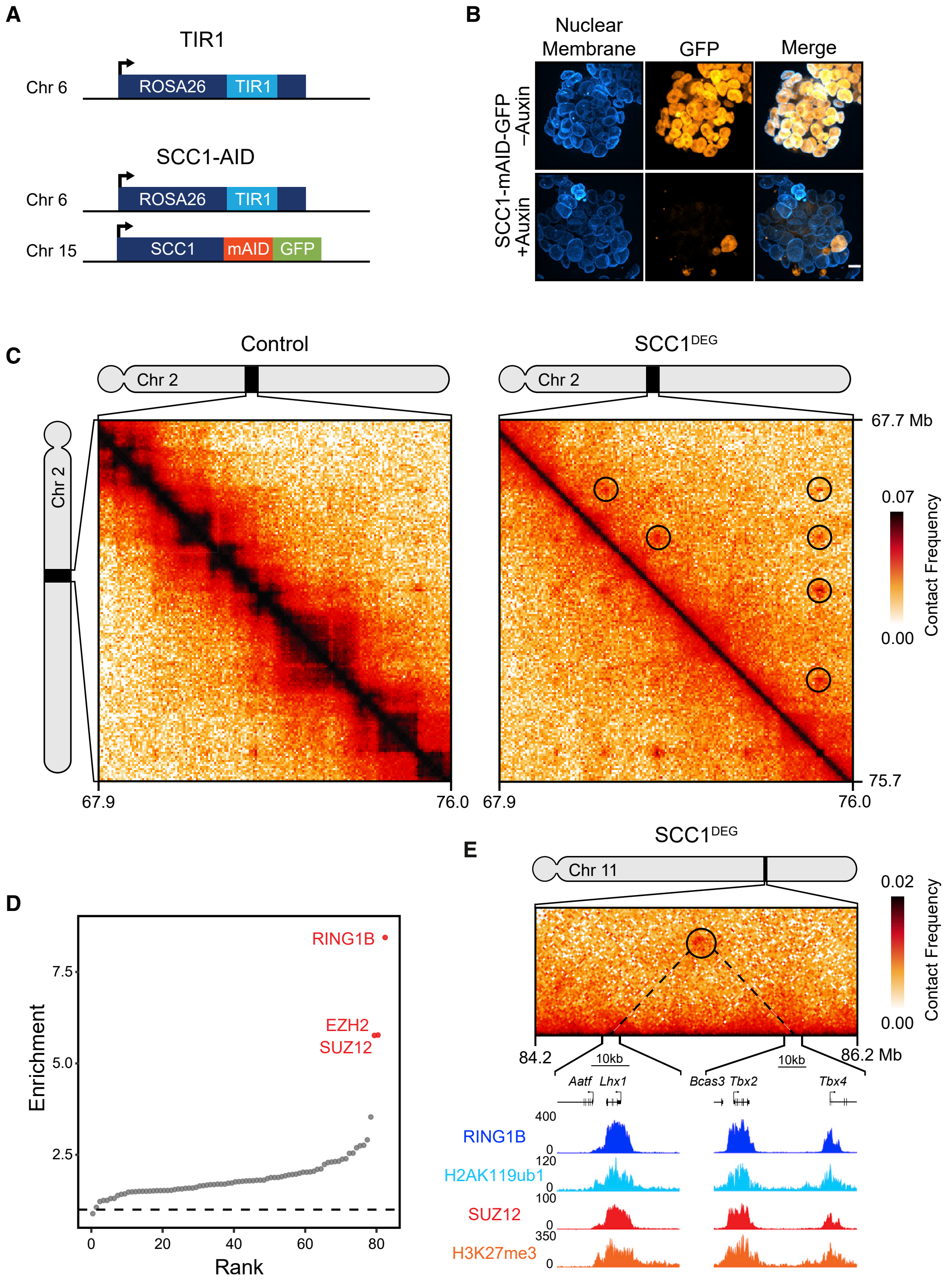

Figure 1. Cohesin-Independent Chromosomal Interactions Correspond to Polycomb Chromatin Domains in ESCs

(A) A schematic illustrating the genotype of the TIR1 and SCC1-mAID-GFP cell lines developed for Hi-C.

(B) Immunofluorescence microscopy images of SCC1-mAID-GFP ESCs ± auxin (6 h). The nuclear membrane was labeled with an antibody against lamin B1. Scale bar, 10 μm (bottom).

(C) Hi-C in control (TIR1 line + auxin) (left) and SCC1^{DEG} (SCC1-mAID-GFP line + auxin) (right) cells after auxin treatment visualized at 40-kb resolution. Peaks identified on the SCC1^{DEG} Hi-C matrix are shown as black circles. The genomic coordinates are illustrated below and to the right of the matrices.

(D) Enrichment of histone modifications and proteins at paired interaction sites compared to the enrichments at matched random interaction sites.

(E) Chromatin immunoprecipitation sequencing (ChIP-seq) snapshot illustrating RING1B, H2AK119ub1, SUZ12, and H3K27me3 under an interaction that persists in the absence of cohesin. The Hi-C matrix is shown above at 20-kb resolution. ChIP-seq datasets used for this figure are indicated in Table S1.

OPEN ACCESS
CellPress

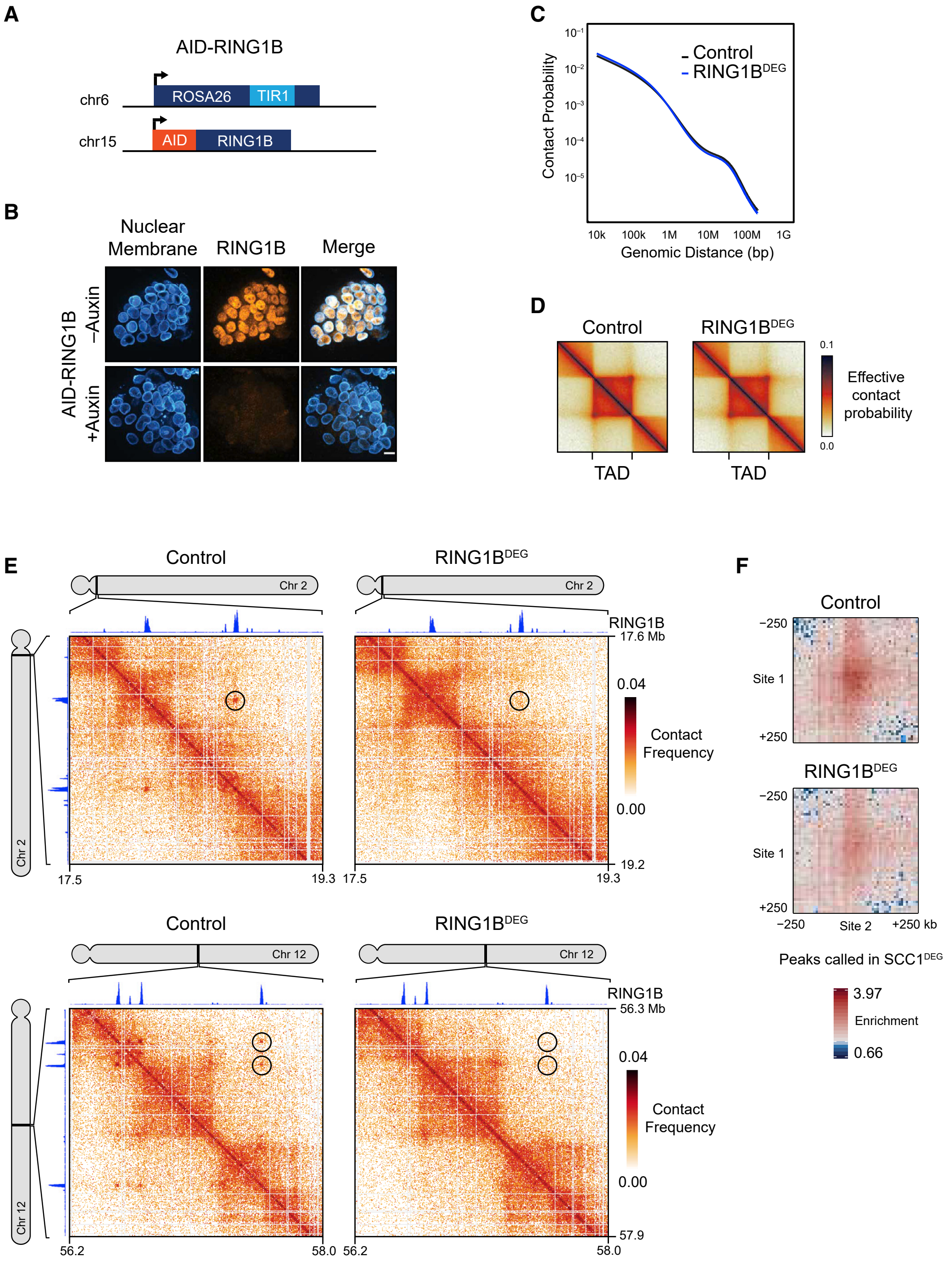

Figure 2. Polycomb Mediates Interactions that Persist in the Absence of Cohesin

(A) A schematic illustrating the genotype of the AID-RING1B cell line.

(B) Immunofluorescence microscopy images of AID-RING1B ESCs ± auxin (6 h). The cells were labeled with antibodies against lamin B1 and RING1B. Scale bar, 10 μm.

(C) Genomic-distance-dependent contact probability from Hi-C in control or RING1B^{DEG} (AID-RING1B + auxin) cells.

(D) Aggregate TAD analysis of control and RING1B^{DEG} cells at 10-kb resolution. Effective contact probability is displayed at a published set of TAD intervals from ESC Hi-C (Bonev et al., 2017).

(E) Hi-C in control and RING1B^{DEG} cells at interactions that persist in the absence of cohesin (black circles) at 5-kb resolution. RING1B ChIP-seq is displayed above and to the left of the matrices.

(F) Aggregate analysis of Hi-C from control and RING1B^{DEG} cells at interactions that persist in the absence of cohesin (n = 336).

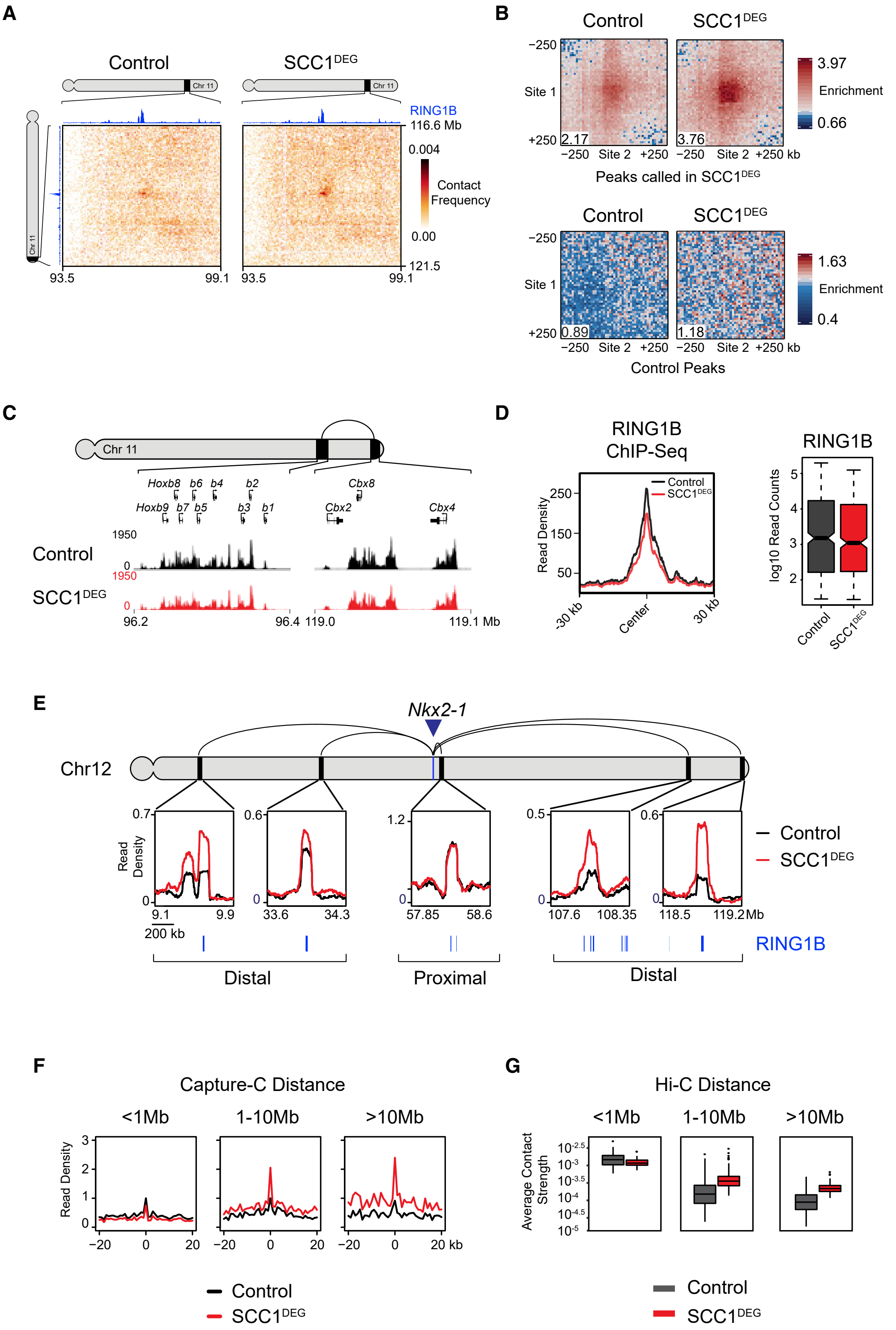

(legend on next page)

slightly lower, than in cells with normal cohesin levels (Figures 3C and 3D). This reveals that cohesin regulates polycomb chromatin domain interactions without affecting PRC1 occupancy.

To further explore polycomb-dependent interactions and their regulation by cohesin, we used a technique called Capture-C that has an advantage over Hi-C in providing increased sensitivity and resolution for interrogating interactions at specific regions in the genome (Hughes et al., 2014). Using Capture-C, we focused on 18 genes that are associated with polycomb chromatin domains and examined their interactions following removal of cohesin. Interestingly, our analysis demonstrated that interactions between polycomb chromatin domains that are in close proximity tended to be unchanged or slightly reduced in the absence of cohesin (Figures 3E, 3F, and S3D–S3I). In contrast, interactions that occurred over long distances, often between different TADs, showed increases in their interaction strength and again this was more pronounced than the modest increases in compartmentalization that result from cohesin removal (Figures 3E, 3F, and S3D–S3G). Importantly, these interactions were lost following PRC1 removal, demonstrating that they rely on intact polycomb chromatin domains (Figures S3C–S3E). This distance-dependent effect was also evident when we examined cohesin-independent interactions in our Hi-C analysis (Figures 3G and S3H), but not when we examined interactions between other repressed gene promoters that lack polycomb chromatin domains (Figure S3J).

Removal of cohesin, even under our rapid inactivation conditions, is likely to have effects on cell-cycle progression given that cohesin has an essential role in sister chromatid cohesion and mitotic progression (Peters et al., 2008). Indeed, when we examined cohesin-depleted cells, there was a slight accumulation of mitotic cells compared to the control (Figures S4A and S4B). To ensure that the effects on polycomb chromatin domain interactions were not due to cell-cycle alterations, we treated cells with nocodazole for 6 h to induce a similar increase in mitotic cells (Figures S4A and S4B) and repeated our Capture-C analysis (Figures S4C–S4E). Importantly, we observed that polycomb chromatin domain interactions were unchanged, or slightly weakened, demonstrating that the effect of cohesin on polycomb chromatin domains is independent of its role in mitotic progression. Therefore, cohesin has little effect on interactions between polycomb chromatin domains that are in close proximity on the chromosome but counteracts interactions between polycomb chromatin domains separated by large distances independently of its effects on the cell cycle.

The Effect of Cohesin on Polycomb Chromatin Domains Is Cell-Type Specific

Having identified a role for cohesin in regulating polycomb chromatin domain interactions, we were keen to understand whether this was unique to ESCs or shared among other cell types. To address this interesting question, we examined interactions between polycomb chromatin domains using Hi-C from other cell types where cohesin function had been perturbed (Figure 4A). In mouse hepatocytes (Schwarzer et al., 2017), we observed some evidence for association between polycomb chromatin domains, and these associations became slightly stronger in the absence of the cohesin loader complex component NIPBL, which also causes loss of cohesin from chromosomes. However, polycomb chromatin domain interactions appeared much weaker and effects on interactions following cohesin removal were more subtle than in ESCs. In contrast, in three different cancer cell lines, HAP1 (Haarhuis et al., 2017), HCT116 (Rao et al., 2017), and HeLa (Wutz et al., 2017), we did not observe obvious punctate interactions between polycomb chromatin domains in control cells, and these sites did not interact following removal of SCC1 (HCT116 and HeLa) or removal of the cohesin loader component SCC4 (HAP1). Together, these observations suggest that the role that cohesin plays in disrupting strong polycomb chromatin domains interactions is specific to ESCs, where the polycomb system is highly expressed and known to play a central role in gene regulation (Boyer et al., 2006; Endoh et al., 2008; Lee et al., 2006).

Previously, it has been reported that interactions between super-enhancers increased in a human cancer cell line (HCT116) when SCC1 was depleted (Rao et al., 2017). Therefore, we were keen to explore whether this effect was also cell-type specific. When we reanalyzed Hi-C data in SCC1-depleted HCT116 cells, we also found evidence for increased interactions between super-enhancers, particularly over long distances (Figure 4B). A similar effect on super-enhancer interactions was also evident in mouse hepatocytes (Schwarzer et al., 2017) and another human

Figure 3. Cohesin Removal Strengthens Long-Range Polycomb Chromatin Domain Interactions

(A) Hi-C illustrating an interaction that increases in strength in the SCC1^{DEG} cell line visualized at 20-kb resolution. RING1B ChIP-seq is displayed above and to the left of the matrices.

(B) Aggregate analysis of Hi-C from control and SCC1^{DEG} cells at peaks that persist in the absence of cohesin (n = 336) (top panels) or at a set of distance matched control sites in B compartments (bottom panels). In each aggregate plot, the trimmed mean of the enrichment scores at the center 50 kb of the aggregated matrix is displayed in the bottom left corner as a quantification of enrichment.

(C) ChIP-seq for RING1B in control and SCC1^{DEG} cells at the interacting sites shown in (A).

(D) RING1B ChIP-seq signal (metaplots, left; boxplots, right) at RING1B peaks overlapping interactions that persist in the absence of cohesin.

(E) Capture-C interaction profiles between the *Nkx2-1* promoter and selected proximal and distal RING1B-occupied sites in control and SCC1^{DEG} cells. RING1B ChIP-seq peaks are shown as blue bars below. The location of the *Nkx2-1* promoter is indicated with a blue arrow/bar and the interactions sites as black bars on the chromosome. Read density corresponds to normalized reads in the capture averaged across 250 DpnII restriction fragments.

(F) Aggregate Capture-C signal in control and SCC1^{DEG} cells at interaction sites segregated based on distance from the capture site. Only interactions between polycomb target gene promoters and RING1B-occupied sites present in SCC1^{DEG} are shown. Read density was normalized to control signal at the summit and the x axis illustrates the distance from the interaction site in DpnII fragments.

(G) Average Hi-C contact strength in the control and SCC1^{DEG} at interactions that persist in the absence of cohesin segregated based on distance between the interactions.

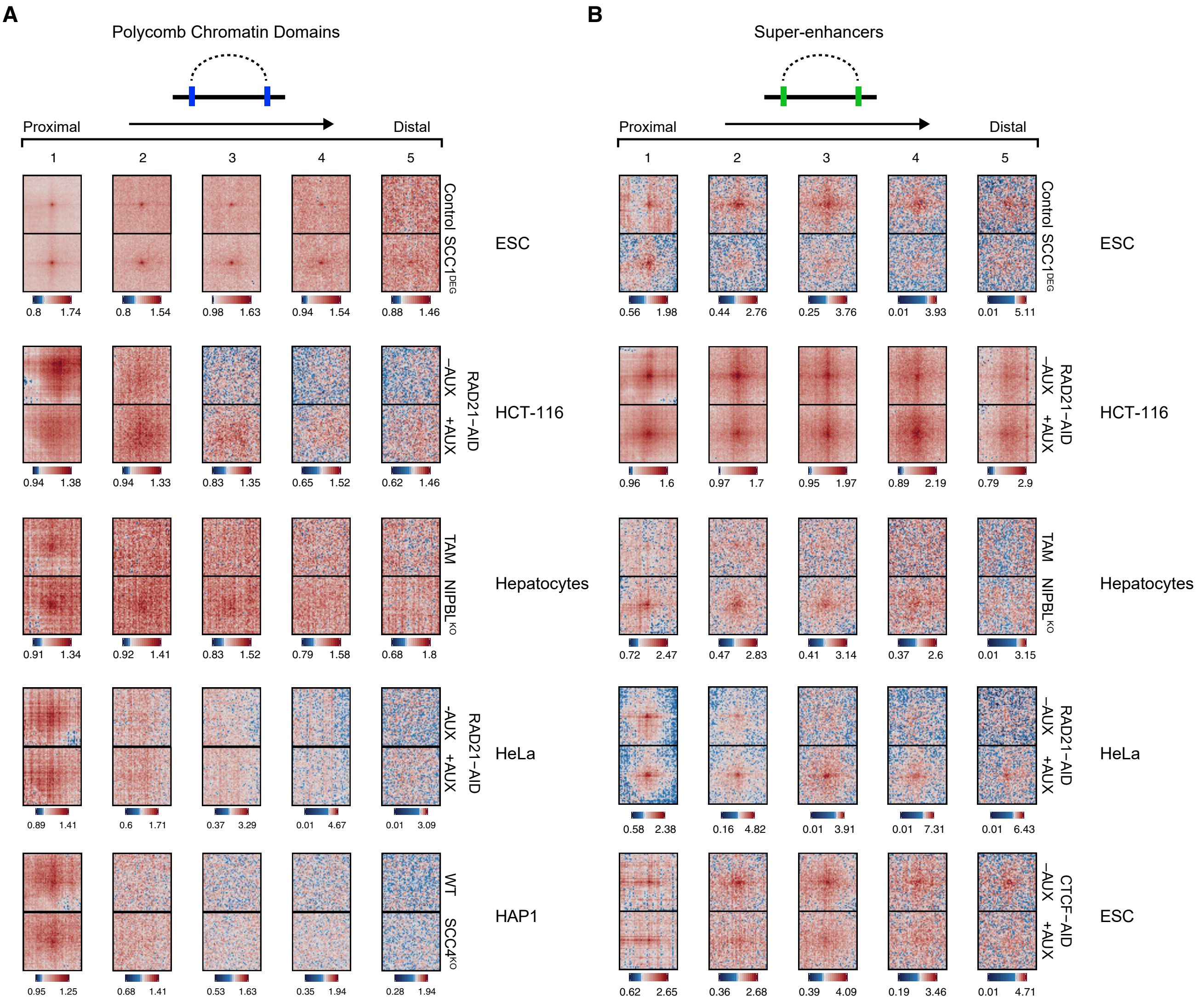

Figure 4. The Effect of Cohesin on Polycomb Chromatin Domains Is Cell-Type Specific

(A) Aggregate peak analysis of Hi-C for interactions between polycomb domains upon perturbation of cohesin in ESCs, HCT116 (Rao et al., 2017), mouse hepatocytes (Schwarzer et al., 2017), HeLa (Wutz et al., 2017), and HAP1 (Haarhuis et al., 2017). Interactions were segregated into five equal-sized quantiles based on distance, with quantile 1 being proximal interactions and quantile 5 the most distal interactions.

(B) Top four groups are as in (A), but for interactions between super-enhancers in ESCs, HCT116 (Rao et al., 2017), mouse hepatocytes (Schwarzer et al., 2017), and HeLa (Wutz et al., 2017). Bottom group illustrates effects on super-enhancer interactions following depletion of CTCF in ESCs (Nora et al., 2017).

cancer cell line (HeLa) (Wutz et al., 2017). However, interestingly, cohesin depletion in ESCs led to a completely distinct effect on super-enhancers. Interactions between super-enhancers were evident in control cells, but in cohesin-depleted cells, these interactions, in contrast to other cell types, were weakened or lost (Figure 4B). This effect was also observed in cells where CTCF was depleted (Nora et al., 2017), suggesting that cohesin and CTCF support, as opposed to disrupt, super-enhancer interactions in ESCs. Together, these observations reveal an unexpected cell-type-specific role for cohesin in regulating chromosomal interactions in ESCs, with cohesin supporting super-enhancer interactions and disrupting long-range polycomb chromatin domain interactions.

Cohesin Counteracts Polycomb Chromatin Domain Interactions Independently of CTCF and Insulation

The capacity of cohesin to disrupt polycomb chromatin domain interactions could be related to a process inherent to cohesin, like loop extrusion, or rely on cohesin functioning with CTCF, as appears to be the case for super-enhancers. To distinguish between these possibilities, we carried out Capture-C in a cell line where insulation is disrupted by removal of CTCF but cohesin and loop extrusion are retained (Figures 5A, 5B, S5A, and S5B; Nora et al., 2017). In contrast to the loss of cohesin, removal of CTCF did not strengthen distal polycomb chromatin domain interactions (Figures 5D, 5E, S5C, and S5D). This was also evident when we examined the interactions that persisted in

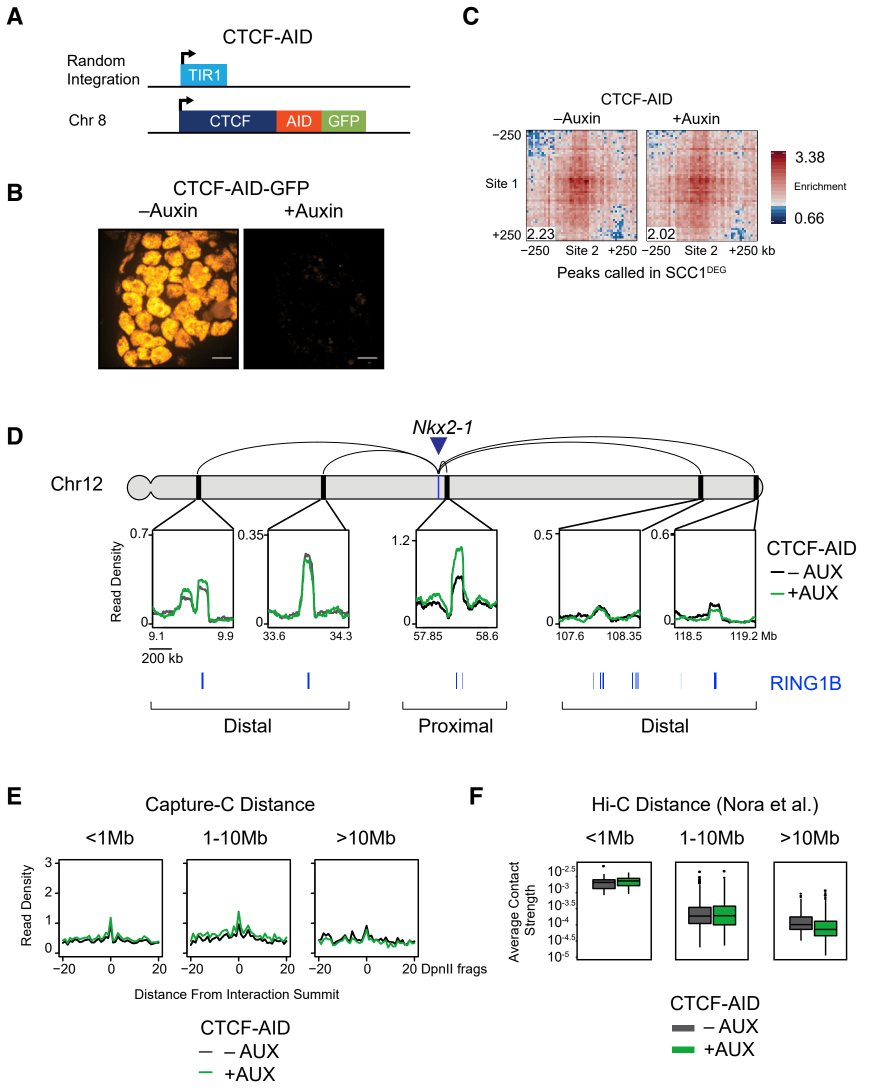

Figure 5. Cohesin Counteracts Polycomb Chromatin Domain Interactions Independently of CTCF and Insulation

(A) A schematic illustrating the genotype of the CTCF-AID-GFP cell line (Nora et al., 2017).

(B) Live-cell microscopy images of CTCF-AID-GFP cells ± auxin (48 h). Scale bar, 10 μm (bottom).

(C) Aggregate analysis of Hi-C from CTCF-AID-GFP cells ± auxin (Nora et al., 2017) at interactions that persist in the absence of cohesin (n = 336). In each aggregate plot, the trimmed mean of the enrichment scores at the center 50 kb of the aggregated matrix is displayed in the bottom left corner as a quantification of enrichment.

(D) Capture-C interaction profiles between the *Nkx2-1* promoter and selected proximal and distal RING1B-occupied sites in the CTCF-AID-GFP cells ± auxin. RING1B ChIP-seq peaks are shown as blue bars below. The location of the *Nkx2-1* promoter is indicated with a blue arrow/bar and the interactions sites as black bars on the chromosome. Read density corresponds to normalized reads in the capture averaged across 250 DpnII restriction fragments.

(E) Aggregate Capture-C signal in the CTCF-AID-GFP cells ± auxin at interactions that persist in the absence of cohesin segregated based on distance from the capture site. Only interactions between polycomb target gene promoters and RING1B-occupied sites present in $SCC1^{DEG}$ are shown. Read density was normalized to signal at the summit in CTCF-AID-GFP cells without auxin, and the x axis illustrates the distance from the interaction site in DpnII fragments.

(F) Average Hi-C contact strength in the CTCF-AID-GFP cells ± auxin at interactions that persist in the absence of cohesin segregated based on distance between the interactions.

the absence of cohesin after CTCF removal using published Hi-C in the same cell line under the same conditions (Nora et al., 2017; Figures 5C and 5F). Therefore, cohesin counteracts polycomb chromatin domain interactions through a process that is independent of CTCF and insulation.

Polycomb Chromatin Domain Interactions Are Disrupted by Cohesin

Chromosome-conformation-capture-based approaches are extremely sensitive and can identify infrequent interaction events like those that lead to the emergence of TADs in ensemble Hi-C analysis. However, neither Hi-C nor Capture-C reveals the absolute frequency of these interactions. Therefore, to characterize polycomb chromatin domain interactions and define the extent to which cohesin regulates these, we set out to measure interactions in single cells. We focused on a pair of genes (*HoxD10* and *Dlx2*) with polycomb chromatin domains that showed increased interaction in Hi-C (Figure 1C) and Capture-C (Figure 6A) after cohesin removal. We generated probes that uniquely mark the *HoxD10/Dlx2* loci and a second set for similarly interacting polycomb target genes, *Nkx2-3/Pax2* (Figure S7), and performed non-denaturing RASER-FISH (Brown et al., 2018) to measure the three-dimensional distance between these loci in individual cells (Figures 6B, S6C, and S7A). This revealed a distribution of distances in cells where cohesin is intact, including some in close proximity (Figures 6C, S6D, S7B, and S7D). When cohesin was removed, the number of very close distances became larger, in agreement with increased contact probabilities between polycomb chromatin domains observed in Hi-C and Capture-C (Figure 3). Furthermore, to test whether this was dependent on both the inactivation of cohesin and the presence of PRC1, we developed a double degron line where SCC1 and RING1B were depleted simultaneously by addition of auxin (Figure S6A). Capture-C in this line revealed a loss of interaction between *HoxD10* and *Dlx2* (Figure S6B). Similarly, in the absence of both cohesin and PRC1, the number of very close distances between *HoxD10/Dlx2* and *Nkx2-3/Pax2* in fluorescence *in situ* hybridization (FISH) was greatly reduced (Figures 6C and S7B). Together, this demonstrates that cohesin counteracts polycomb chromatin domain association in single cells.

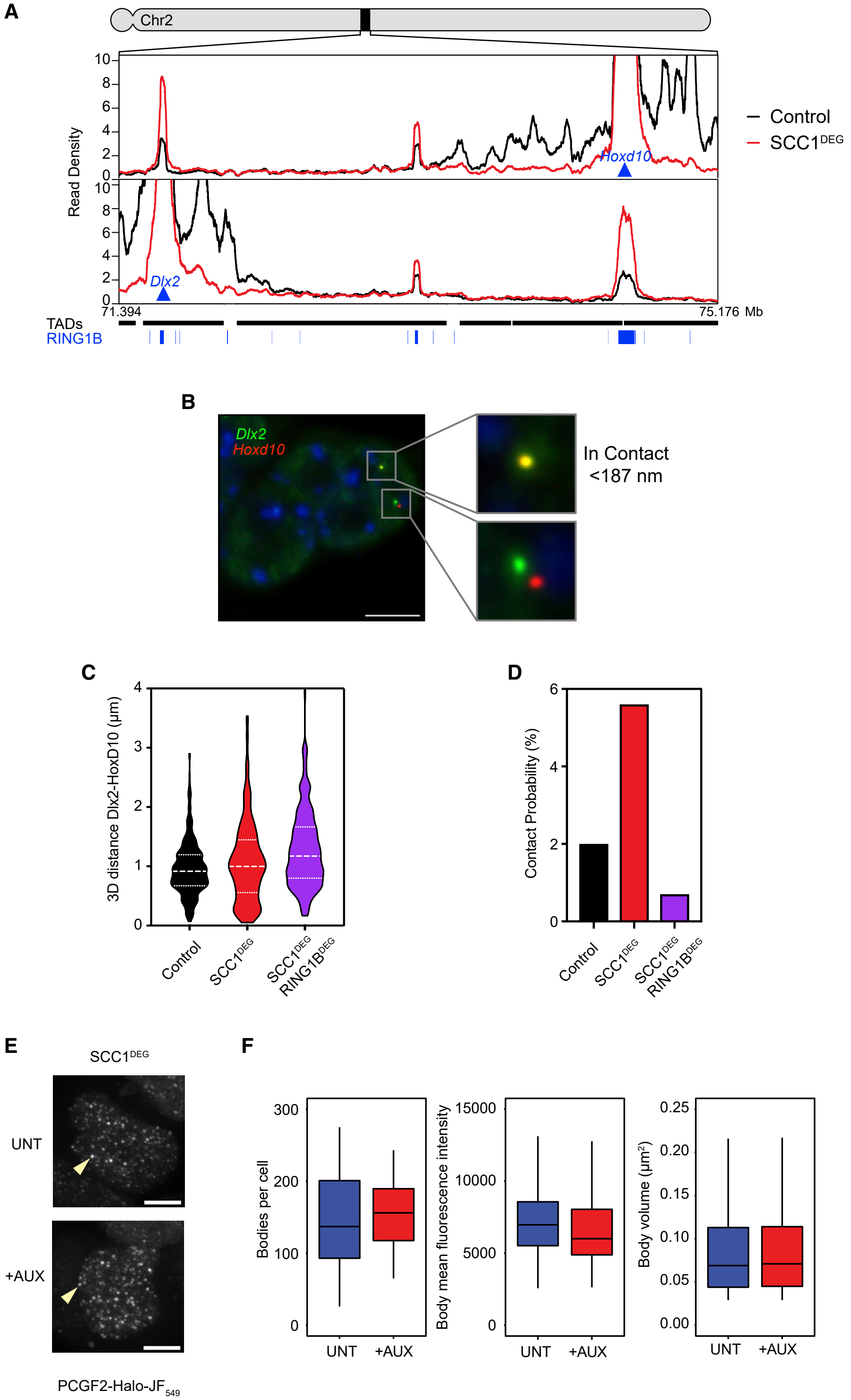

(legend on next page)

Previous studies have reported that loci that display strong interactions in Hi-C do not always equate to frequent interactions in single cells (Bonev et al., 2017; Fudenberg and Imakaev, 2017). Therefore, we wanted to accurately quantitate the frequency of polycomb chromatin domain interactions by determining the number of FISH probe measurements that may be considered to be in contact (Cattoni et al., 2017). This revealed that *HoxD10* and *Dlx2* polycomb chromatin domains were in contact (closer than 187 nm) 2% of the time (Figure 6D). Following cohesin removal, the contact frequency increased to 5.6%, indicating that when cohesin is present, it functions to disrupt interactions between regions of chromatin occupied by the polycomb repressive system. Importantly, this increased association between polycomb chromatin domains was dependent on cohesin and PRC1, as their simultaneous removal reduced the interaction frequency to 0.7%. Again, we observed very similar interaction frequencies when we examined the polycomb chromatin domains associated with the *Nkx2-3* and *Pax2* genes (Figure S7C). Furthermore, these interactions relied on the polycomb system, as their frequency upon removal of PRC1 alone was similar to their frequency upon removal of both PRC1 and cohesin (Figure S7C). It is also important to point out that these interaction values likely underestimate the absolute frequency with which polycomb chromatin domains interact with one another, as interactions between specific pairs of sites are likely to vary between individual cells.

In ESCs, polycomb proteins are enriched in more than 100 cytologically distinct foci called polycomb bodies in which polycomb chromatin domain interactions have been reported to occur (Blackledge et al., 2019; Isono et al., 2013; Ren et al., 2008). Given the increased association between polycomb chromatin domains following cohesin removal, we were curious whether this might also affect the properties of polycomb bodies. To examine this possibility, we imaged polycomb bodies in control and cohesin-depleted cells (Figures 6E and 6F). Interestingly, following cohesin removal, there were no overt changes in the number, intensity, or volume of polycomb bodies. There was a slight trend toward more numerous and less intense polycomb bodies, but the relevance of these minor alterations would require more detailed study. Nevertheless, our observations suggest that these polycomb body features are not directly linked to the increases in polycomb chromatin domain interactions we measure in Hi-C, Capture-C, and DNA-FISH following cohesin removal (Figures 3 and 6). This is consistent with our recent findings that the number of polycomb bodies in ESCs is only modestly affected when polycomb chromatin domain interactions are lost (Blackledge et al., 2019). Therefore, it remains unclear to what extent polycomb bodies represent interactions between polycomb chromatin domains or simply an accumulation of polycomb proteins at individual domains. Nevertheless, our single-cell measurements quantitate the frequency with which polycomb chromatin domains interact in single cells and demonstrate that cohesin disrupts polycomb chromatin domain interactions despite not significantly altering polycomb bodies.

Increased Polycomb Chromatin Domain Association in the Absence of Cohesin Suppresses Gene Expression

In vertebrates, polycomb repressive complexes play important roles in maintaining the repression of genes in cell types where they should not be expressed (Schuettengruber et al., 2017). This is proposed to rely on chromatin modifications and, in some instances, the formation of polycomb-dependent chromatin interactions (Eskeland et al., 2010; Kundu et al., 2017). Here, we demonstrate that cohesin counteracts and disrupts long-range interactions between polycomb chromatin domains and their associated genes. We were therefore interested to test whether cohesin affects polycomb-mediated gene repression. To examine this possibility, we performed calibrated RNA-seq (cRNA-seq) before and after cohesin removal. In agreement with previous analysis following cohesin depletion, changes in gene expression were modest, and the transcription of only several hundred genes was significantly altered (Rao et al., 2017; Figure 7A). Nevertheless, we also observed a more subtle and widespread reduction in gene transcription, in agreement with a proposed role for cohesin in supporting promoter-enhancer interactions and gene expression (Hadjur et al., 2009; Schwarzer et al., 2017). Remarkably, however, RING1B-bound genes were overrepresented (251/365) among the genes whose expression was significantly reduced, indicating that they were disproportionally affected (Figure 7B). Importantly, these alterations in transcription were validated by RNA-FISH (Figures S8A and S8B), independent of any effects on the cell cycle (Figure S8C), and tended to correspond to genes that have particularly high levels of polycomb, but not trithorax-associated, chromatin modifications (Figures S8D and S8E). A more detailed analysis of polycomb-bound genes with detectable expression (Figures S8I and S8J) in our cRNA-seq showed that reductions in expression were larger in magnitude following cohesin removal if the gene interacted with another polycomb chromatin domain (Figures 7C and 7D). In contrast,

Figure 6. Polycomb Chromatin Domain Interactions Are Disrupted by Cohesin

(A) Capture-C interaction profiles from HoxD10 (top) and Dlx2 (bottom) viewpoints in control and SCC1^{DEG} lines. RING1B ChIP-seq peaks are displayed as blue bars and TAD intervals as black bars.

(B) Representative image of RASER-FISH showing signals classed as in contact (top pair, 0.0905 μm apart) and not in contact (bottom pair, 1.1629 μm apart). Probes are for Dlx2 (green) and HoxD10 (red). Scale bar, 5 μm.

(C) Violin plots showing 3D distance measurements between Dlx2 and HoxD10 in the indicated cells lines. The dashed lines show the median and interquartile range of n = 376–409 alleles for each cell line.

(D) Absolute contact probabilities showing the percent of signals judged as colocalized from observations in (B) and (C) (see STAR Methods).

(E) Maximum intensity projections of PCGF2-Halo-JF549 SCC1-mAID-GFP cells before (UNT) and after auxin (6 h) treatment (+AUX). Example nuclear foci (polycomb bodies) are indicated by arrowheads. Scale bar, 5 μm.

(F) Boxplots comparing the number of polycomb bodies per cell in PCGF2-Halo-JF549 SCC1^{DEG} ESCs untreated (35 cells, UNT, blue) and after auxin (6 h) treatment (28 cells, +AUX, red) (left). Mean fluorescence intensity (middle) and volume (right) of polycomb bodies before (5283 foci, UNT, blue) and after auxin (6 h) treatment (4321 foci, +AUX, red).

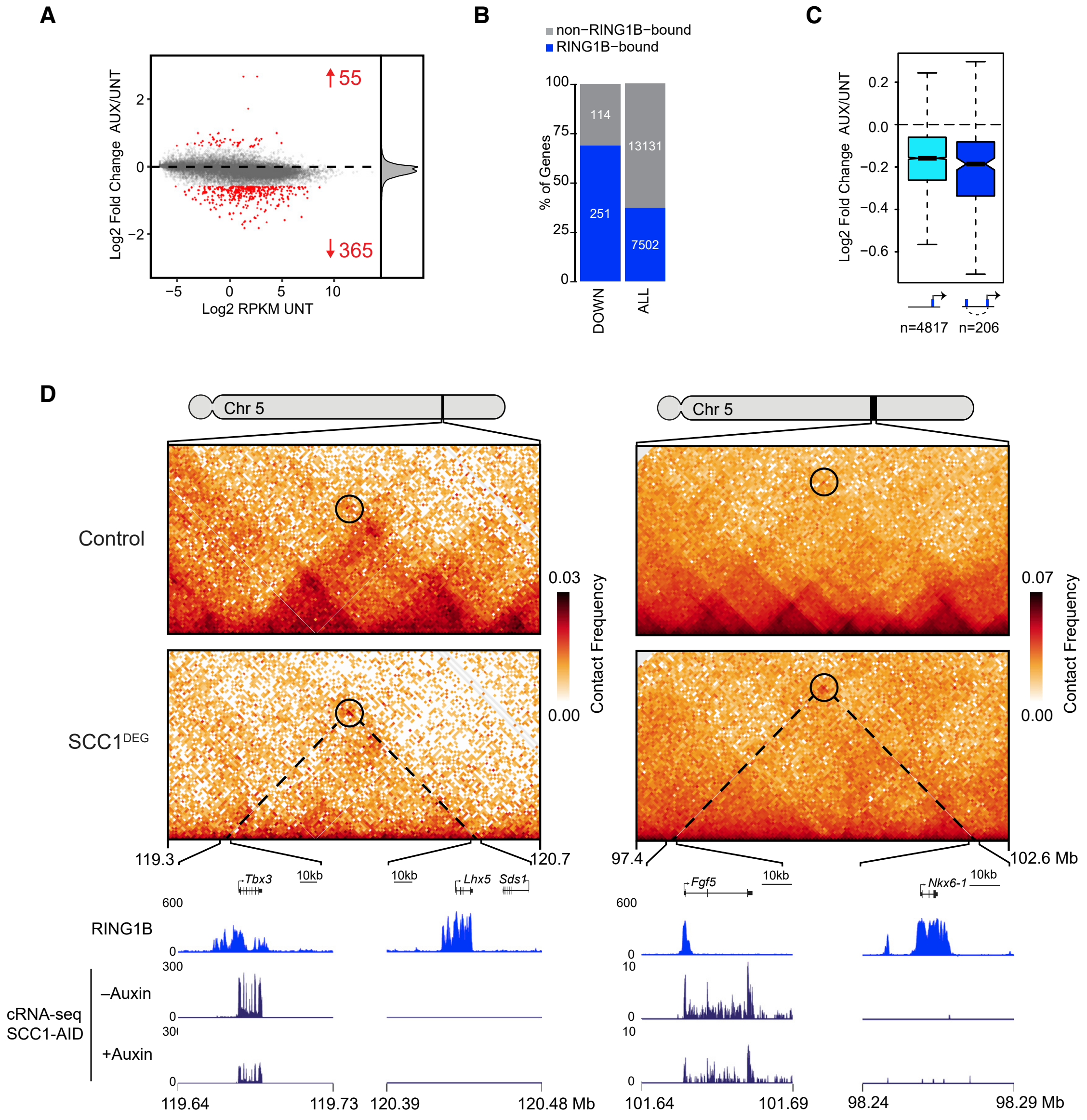

Figure 7. Increased Polycomb Chromatin Domain Association in the Absence of Cohesin Suppresses Gene Expression

(A) An MA plot of gene expression alterations in Scc1-mAID-GFP cells ± auxin (6 h). The number of genes with increased or decreased expression (p-adj < 0.05 and > 1.5-fold) is shown in red. The density of log2 fold changes is shown on the right.

(B) RING1B binding (±1 kb from the TSS (transcription start site), blue bars) at gene promoters that show reductions in gene expression following cohesin removal (left) compared to all genes (right). Empirical p value for RING1B-bound genes enrichment within the downregulated genes: $p < 0.0001$ (n = 10,000 random tests).

(C) The magnitude of gene expression change at expressed RING1B-bound genes that do (right) or do not (left) interact with another RING1B-bound site in Hi-C.

(D) Hi-C (left at 40-kb and right at 10-kb resolution), cRNA-seq, and RING1B ChIP-seq for two examples of genes with interactions in Hi-C that are strengthened after cohesin removal and whose gene expression decreases.

the much smaller number of upregulated genes, although slightly enriched for polycomb chromatin domains (Figure S8F), was on average closer to TAD boundaries and nearby super-enhancers (Figure S8G). Interestingly, these genes also increased in expression following CTCF depletion (Figure S8H; Nora et al., 2017), suggesting the effects on expression may be due to loss of insulation. Together, these observations suggest that cohesin, and presumably its loop-extruding activity, play a direct role in counteracting long-range polycomb chromatin domain interactions and modulating gene repression by the polycomb system.

DISCUSSION

How cohesin functions to shape chromosome structure and function remains poorly understood. Here, using degron

alleles and chromosome conformation capture approaches, we identify a series of long-range interactions that persist in the absence of cohesin and correspond to polycomb chromatin domains (Figure 1). We demonstrate that PRC1 is essential for the formation of these interactions (Figure 2). In the absence of cohesin, polycomb chromatin domain interactions are strengthened, revealing that they are normally counteracted by cohesin (Figure 3). This effect on strong polycomb chromatin domain interactions is unique to ESCs where polycomb protein levels are high and the polycomb system is known to play important roles in gene regulation (Figure 4). Importantly, cohesin regulates these interactions independently of CTCF and insulation (Figure 5). Using cellular imaging, we visualize polycomb chromatin domain interactions, quantify their frequency, and further demonstrate a role for cohesin in disrupting polycomb chromatin domain interactions in single cells independently of effects on polycomb bodies (Figure 6). Finally, we demonstrate that regulation of polycomb chromatin domain interactions by cohesin appears to modulate expression of polycomb target genes (Figure 7). These findings reveal a link between the capacity of the polycomb system to form long-range transcriptionally repressive chromosome interactions and cohesin that appears to actively counteract and regulate this process (Figure S9).

Initially, the observation that cohesin disrupts repressive polycomb chromatin domain interactions may seem counterintuitive. Why would it be advantageous for a cell to disrupt chromosomal interactions, like those formed between polycomb chromatin domains, which function to protect against inappropriate gene expression? One simple explanation may be that, if left unchecked, progressive association by factors that nucleate and promote repressive chromatin interactions could lead to an irreversibly silent state. In pluripotent cells or at early developmental stages, such a static situation could be deleterious because many genes that are occupied by polycomb chromatin domains, and that engage in long-range interactions, must be expressed later in development. This may explain why cohesin disrupts polycomb chromatin domain interactions in ESCs. Polycomb chromatin domain interactions have been proposed to occur within polycomb bodies (Bantignies et al., 2011; Isono et al., 2013), and polycomb bodies have recently been linked to phase separation (Plys et al., 2019; Tatavosian et al., 2019). However, we find that increases in polycomb chromatin domain interactions following cohesin removal have very little effect on polycomb body features, and removing polycomb chromatin domain interactions has only a modest effect on polycomb bodies (Blackledge et al., 2019). This suggests that polycomb chromatin domain interactions are not inextricably linked to polycomb bodies and phase separation. Nevertheless, based on the capacity for cohesin to disrupt polycomb chromatin domain interactions, it is tempting to speculate that cohesin may function ubiquitously on interphase chromosomes to counteract the potential for stasis, in agreement with predictions from polymer modeling and simulations of loop extrusion (Nuebler et al., 2018). By periodically breaking up self-associating chromatin domains, loop extrusion may provide an opportunity for factors in the nucleus to constantly sample these regions of the genome should they be required for future gene expression programs. Based on these observations, it will be important in future work to understand in more detail the relevance of these effects on polycomb chromatin domain interactions and gene expression in stem cell biology, differentiation, and development.

Interestingly, interactions between super-enhancers in cancer cells were previously shown to occur independently of cohesin, and long-range super-enhancer associations were also increased following cohesin removal (Rao et al., 2017). Conceptually aligned with the idea that cohesin and loop extrusion may counteract polycomb chromatin domain interactions to mitigate stasis, one could envisage how periodically disrupting super-enhancer associations and possibly their interactions with gene promoters might support a constant reevaluation of gene-regulatory interactions. To test the generality of these observations, we examined whether removal of cohesin had a similar effect on super-enhancers in ESCs. Unlike the situation in cancer cells where cohesin counteracts super-enhancer interaction, in ESCs, cohesin was required for super-enhancer interactions, and this also relied on CTCF. This interesting observation suggests that the processes by which cohesin supports super-enhancer interactions in ESCs are distinct from those that disrupt polycomb chromatin domain interactions, as the depletion of cohesin, but not CTCF, stabilizes polycomb chromatin domain interactions. More importantly, this also demonstrates that cohesin can have remarkably cell-type-specific effects on chromosomal interactions. This was further evident when we examined polycomb chromatin domain interactions in cancer cells where long-range interactions were not present in wild-type cells, nor did they increase when cohesin was removed. These observations argue that detailed analysis of cohesin function in diverse cell types may reveal new and unexpected roles for cohesin in shaping chromosomal interactions.

We demonstrate that cohesin plays an important role in disrupting polycomb chromatin domain interactions. Although the defined mechanisms by which this is achieved remain to be determined, we speculate that this may rely on the topological manner in which entrapped chromatin would extrude through the cohesin complex; however, translocation of cohesin through polycomb domains by other mechanisms could also disrupt interactions. We envisage that loading of cohesin on the chromosome in proximity to a polycomb chromatin domain, followed by loop extrusion, could break up interactions with other polycomb chromatin domains, either by transiently displacing PRC1 or breaking the biochemical links between polycomb chromatin domains, irrespective of whether they are separated by large distance on the chromosome or even between chromosomes (Figure S9). This would also explain why CTCF and its proposed activity in halting extrusion would not affect the ability of cohesin to counteract polycomb chromatin domain interactions as we observe. Instead, CTCF and termination of loop extrusion may function to restrict the activity of gene regulatory elements to regions between CTCF sites by limiting mixing of chromatin that might result from unconstrained loop extrusion. To further understand how cohesin disrupts polycomb chromatin domains, it will also be important in

future work to dissect which components of the cohesin complex are required for this process. It was recently shown using small interfering RNA (siRNA)-based knockdown approaches that these effects may rely in part on the SA1 paralog of the cohesin subunit stromal antigen (SA) (Cuadrado et al., 2019). However, determining the relevance of these effects and the contribution of other cohesin complex subunits to the disruption of polycomb chromatin domain interactions will require rapid degron-based depletion approaches that mitigate secondary effects inherent to knockdown approaches.

Finally, cohesin is best characterized for the role it plays in holding sister chromatids together after replication and during cell division. In contrast, other structural maintenance of chromosomes (SMC) complexes, for example bacterial SMC-ScpAB and eukaryotic condensin, have been proposed to play roles in separating chromosomes through processes that are thought to rely on loop extrusion (Goloborodko et al., 2016; Nasmyth, 2001; Wang et al., 2017). Our observations provide evidence to suggest that in addition to its role in sister chromatid cohesion, cohesin also retains its primordial SMC complex activity in separating regions of chromosomes, as is evident from the role it plays in disrupting long-range polycomb chromatin domain interactions.

STAR★METHODS

Detailed methods are provided in the online version of this paper and include the following:

- KEY RESOURCES TABLE
- LEAD CONTACT AND MATERIALS AVAILABILITY
- EXPERIMENTAL MODEL AND SUBJECT DETAILS
  - Cell Culture
  - Protein Degradation and Nocodazole treatment
- METHOD DETAILS
  - Generation, culturing and treatment of cell lines
  - *In situ* Hi-C library generation for low cell input
  - Capture-C
  - Calibrated RNA-seq and ChIP-seq
  - FISH
  - smRNA-FISH
  - Characterization of Polycomb bodies
  - FACS
  - Antibodies
- QUANTIFICATION AND STATISTICAL ANALYSIS
  - Hi-C
  - Intervals for Aggregate Peak Analysis
  - Capture-C
  - Calibrated RNA-seq (cRNA-seq) and ChIP-seq (cChIP-seq)
  - FISH
  - smRNA-FISH
  - Characterization of Polycomb bodies
- DATA AND CODE AVAILABILITY

SUPPLEMENTAL INFORMATION

Supplemental Information can be found online at https://doi.org/10.1016/j.celrep.2019.12.057.

ACKNOWLEDGMENTS

We would like to thank Elphège Nora and Benoit Bruneau for providing the CTCF degron cell line. We would like to thank Neil Brockdorff and Francis Barr for providing comments on the manuscript. We are grateful to Amanda Williams at the Department of Zoology (Oxford, UK) for sequencing support on the NextSeq 500. We are grateful to Jim Hughes, Damien Downes, and Priscila Hirschfeld for providing advice, protocols, and probes for Capture-C prior to publication. We furthermore thank Leah Bury at Whitehead Institute (Cambridge, MA) for help with the model and graphical abstract.

Work in the Klose lab is supported by the Wellcome Trust (209400/Z/17/Z), the European Research Council (681440), and the Lister Institute of Preventive Medicine. A.F. is supported by a Sir Henry Wellcome Trust fellowship. Work in the Nasmyth lab is supported by the Wellcome Trust (grant 107935/Z/15/Z), Cancer Research UK (grant 26747), and the European Research Council (proposal 294401). Work in the Vaquerizas lab is supported by the Max Planck Society, the Deutsche Forschungsgemeinschaft (DFG) Priority Programme SPP2202 Spatial Genome Architecture in Development and Disease (project VA 1456/1), and the Medical Research Council UK. Work in the Buckle lab is supported by the MRC (MC_UU_00016/1) and the Wolfson Imaging Centre Oxford, the Wolfson Foundation (joint MRC/BBSRC/EPSRC), and the Wellcome Trust.

AUTHOR CONTRIBUTIONS

Conceptualization, J.D.P.R., A.F., B.H.-R., N.D., J.M.V., and R.J.K.; Methodology, J.D.P.R., A.F., B.H.-R., N.D., J.M.B., N.A.F., N.P.B., P.D., M.H., A.S., and K.K.; Investigation, J.D.P.R., A.F., B.H.R., N.D., J.M.B., N.A.F., P.D., M.H., A.S., P.P., and K.K.; Formal Analysis, A.F., B.H.-R., N.A.F., M.H., A.S., J.M.B., and K.K.; Resources, J.D.P.R., A.F., N.P.B., P.P., K.K., J.M.B., M.H., and A.S.; Writing – Original Draft, J.D.P.R., A.F., B.H.-R., N.D., J.M.V, and R.J.K.; Writing – Review & Editing, J.D.P.R., A.F., B.H.-R., N.D., N.P.B., N.F., M.H., P.D., K.A.N., V.J.B., J.M.V., and R.J.K.; Funding Acquisition, A.F., K.A.N., V.J.B., J.M.V., and R.J.K.; Supervision, K.A.N., V.J.B., J.M.V., and R.J.K.

DECLARATION OF INTERESTS

The authors declare no competing interests.

Received: March 30, 2019
Revised: November 25, 2019
Accepted: December 16, 2019
Published: January 21, 2020

REFERENCES

Abdennur, N., and Mirny, L. (2019). Cooler: scalable storage for Hi-C data and other genomically-labeled arrays. Bioinformatics *16*, 967.

Atsuta, T., Fujimura, S., Moriya, H., Vidal, M., Akasaka, T., and Koseki, H. (2001). Production of monoclonal antibodies against mammalian Ring1B proteins. Hybridoma *20*, 43–46.

Azuara, V., Perry, P., Sauer, S., Spivakov, M., Jørgensen, H.F., John, R.M., Gouti, M., Casanova, M., Warnes, G., Merkenschlager, M., and Fisher, A.G. (2006). Chromatin signatures of pluripotent cell lines. Nat. Cell Biol. *8*, 532–538.

Bantignies, F., Roure, V., Comet, I., Leblanc, B., Schuettengruber, B., Bonnet, J., Tixier, V., Mas, A., and Cavalli, G. (2011). Polycomb-dependent regulatory contacts between distant Hox loci in Drosophila. Cell *144*, 214–226.

Bernstein, B.E., Mikkelsen, T.S., Xie, X., Kamal, M., Huebert, D.J., Cuff, J., Fry, B., Meissner, A., Wernig, M., Plath, K., et al. (2006). A bivalent chromatin structure marks key developmental genes in embryonic stem cells. Cell *125*, 315–326.

Bintu, B., Mateo, L.J., Su, J.H., Sinnott-Armstrong, N.A., Parker, M., Kinrot, S., Yamaya, K., Boettiger, A.N., and Zhuang, X. (2018). Super-resolution chromatin tracing reveals domains and cooperative interactions in single cells. Science *362*, eaau1783.

Blackledge, N.P., Fursova, N.A., Kelley, J.R., Huseyin, M.K., Feldmann, A., and Klose, R.J. (2019). PRC1 catalytic activity is central to Polycomb system function. Mol. Cell. https://doi.org/10.1016/j.molcel.2019.12.001.

Bonev, B., Mendelson Cohen, N., Szabo, Q., Fritsch, L., Papadopoulos, G.L., Lubling, Y., Xu, X., Lv, X., Hugnot, J.P., Tanay, A., and Cavalli, G. (2017). Multiscale 3D genome rewiring during mouse neural development. Cell *171*, 557–572.e24.

Boyer, L.A., Plath, K., Zeitlinger, J., Brambrink, T., Medeiros, L.A., Lee, T.I., Levine, S.S., Wernig, M., Tajonar, A., Ray, M.K., et al. (2006). Polycomb complexes repress developmental regulators in murine embryonic stem cells. Nature *441*, 349–353.

Brown, J.M., Roberts, N.A., Graham, B., Waithe, D., Lagerholm, C., Telenius, J.M., De Ornellas, S., Oudelaar, A.M., Scott, C., Szczerbal, I., et al. (2018). A tissue-specific self-interacting chromatin domain forms independently of enhancer-promoter interactions. Nat. Commun. *9*, 3849.

Cairns, J., Freire-Pritchett, P., Wingett, S.W., Várnai, C., Dimond, A., Plagnol, V., Zerbino, D., Schoenfelder, S., Javierre, B.M., Osborne, C., et al. (2016). CHiCAGO: robust detection of DNA looping interactions in Capture Hi-C data. Genome Biol. *17*, 127.

Campagne, A., Lee, M.-K., Zielinski, D., Michaud, A., Le Corre, S., Dingli, F., Chen, H., Shahidian, L.Z., Vassilev, I., Servant, N., Loew, D., Pasmant, E., Postel-Vinay, S., Wassef, M., and Margueron, R. (2019). BAP1 complex promotes transcription by opposing PRC1-mediated H2A ubiquitylation. Nat. Commun. *10*, 348.

Cattoni, D.I., Cardozo Gizzi, A.M., Georgieva, M., Di Stefano, M., Valeri, A., Chamousset, D., Houbron, C., Déjardin, S., Fiche, J.B., González, I., et al. (2017). Single-cell absolute contact probability detection reveals chromosomes are organized by multiple low-frequency yet specific interactions. Nat. Commun. *8*, 1753.

Cock, P.J.A., Antao, T., Chang, J.T., Chapman, B.A., Cox, C.J., Dalke, A., Friedberg, I., Hamelryck, T., Kauff, F., Wilczynski, B., and de Hoon, M.J.L. (2009). Biopython: freely available Python tools for computational molecular biology and bioinformatics. Bioinformatics *25*, 1422–1423.

Cournac, A., Marie-Nelly, H., Marbouty, M., Koszul, R., and Mozziconacci, J. (2012). Normalization of a chromosomal contact map. BMC Genomics *13*, 436.

Cuadrado, A., Giménez-Llorente, D., Kojic, A., Rodríguez-Corsino, M., Cuartero, Y., Martín-Serrano, G., Gómez-López, G., Marti-Renom, M.A., and Losada, A. (2019). Specific contributions of cohesin-SA1 and cohesin-SA2 to TADs and polycomb domains in embryonic stem cells. Cell Rep. *27*, 3500–3510.e4.

Davies, J.O.J., Telenius, J.M., McGowan, S.J., Roberts, N.A., Taylor, S., Higgs, D.R., and Hughes, J.R. (2016). Multiplexed analysis of chromosome conformation at vastly improved sensitivity. Nat. Methods *13*, 74–80.

Denholtz, M., Bonora, G., Chronis, C., Splinter, E., de Laat, W., Ernst, J., Pellegrini, M., and Plath, K. (2013). Long-range chromatin contacts in embryonic stem cells reveal a role for pluripotency factors and polycomb proteins in genome organization. Cell Stem Cell *13*, 602–616.

Despang, A., Schöpflin, R., Franke, M., Ali, S., Jerković, I., Paliou, C., Chan, W.L., Timmermann, B., Wittler, L., Vingron, M., et al. (2019). Functional dissection of the Sox9-Kcnj2 locus identifies nonessential and instructive roles of TAD architecture. Nat. Genet. *51*, 1263–1271.

Díaz, N., Kruse, K., Erdmann, T., Staiger, A.M., Ott, G., Lenz, G., and Vaquerizas, J.M. (2018). Chromatin conformation analysis of primary patient tissue using a low input Hi-C method. Nat. Commun. *9*, 4938.

Dixon, J.R., Selvaraj, S., Yue, F., Kim, A., Li, Y., Shen, Y., Hu, M., Liu, J.S., and Ren, B. (2012). Topological domains in mammalian genomes identified by analysis of chromatin interactions. Nature *485*, 376–380.

Dobin, A., Davis, C.A., Schlesinger, F., Drenkow, J., Zaleski, C., Jha, S., Batut, P., Chaisson, M., and Gingeras, T.R. (2013). STAR: ultrafast universal RNA-seq aligner. Bioinformatics *29*, 15–21.

ENCODE Project Consortium (2012). An integrated encyclopedia of DNA elements in the human genome. Nature *489*, 57–74.

Endoh, M., Endo, T.A., Endoh, T., Fujimura, Y., Ohara, O., Toyoda, T., Otte, A.P., Okano, M., Brockdorff, N., Vidal, M., and Koseki, H. (2008). Polycomb group proteins Ring1A/B are functionally linked to the core transcriptional regulatory circuitry to maintain ES cell identity. Development *135*, 1513–1524.

Eskeland, R., Leeb, M., Grimes, G.R., Kress, C., Boyle, S., Sproul, D., Gilbert, N., Fan, Y., Skoultchi, A.I., Wutz, A., and Bickmore, W.A. (2010). Ring1B compacts chromatin structure and represses gene expression independent of histone ubiquitination. Mol. Cell *38*, 452–464.

Finn, E.H., Pegoraro, G., Brandão, H.B., Valton, A.L., Oomen, M.E., Dekker, J., Mirny, L., and Misteli, T. (2019). Extensive heterogeneity and intrinsic variation in spatial genome organization. Cell *176*, 1502–1515.e10.

Flyamer, I.M., Gassler, J., Imakaev, M., Brandão, H.B., Ulianov, S.V., Abdennur, N., Razin, S.V., Mirny, L.A., and Tachibana-Konwalski, K. (2017). Single-nucleus Hi-C reveals unique chromatin reorganization at oocyte-to-zygote transition. Nature *544*, 110–114.

Flyamer, I.M., Illingworth, R.S., and Bickmore, W.A. (2019). Coolpup.py: a versatile tool to perform pile-up analysis of Hi-C data. bioRxiv. https://doi.org/10.1101/586537.

Fudenberg, G., and Imakaev, M. (2017). FISH-ing for captured contacts: towards reconciling FISH and 3C. Nat. Methods *14*, 673–678.

Fudenberg, G., Imakaev, M., Lu, C., Goloborodko, A., Abdennur, N., and Mirny, L.A. (2016). Formation of chromosomal domains by loop extrusion. Cell Rep. *15*, 2038–2049.

Fursova, N.A., Blackledge, N.P., Nakayama, M., Ito, S., Koseki, Y., Farcas, A.M., King, H.W., Koseki, H., and Klose, R.J. (2019). Synergy between Variant PRC1 complexes defines polycomb-mediated gene repression. Mol. Cell *74*, 1020–1036.e8.

Goloborodko, A., Imakaev, M.V., Marko, J.F., and Mirny, L. (2016). Compaction and segregation of sister chromatids via active loop extrusion. eLife *5*, 11202.

Grimm, J.B., English, B.P., Chen, J., Slaughter, J.P., Zhang, Z., Revyakin, A., Patel, R., Macklin, J.J., Normanno, D., Singer, R.H., Lionnet, T., and Lavis, L.D. (2015). A general method to improve fluorophores for live-cell and single-molecule microscopy. Nat. Methods *12*, 244–250.

Guacci, V., Koshland, D., and Strunnikov, A. (1997). A direct link between sister chromatid cohesion and chromosome condensation revealed through the analysis of MCD1 in S. cerevisiae. Cell *91*, 47–57.

Haarhuis, J.H.I., van der Weide, R.H., Blomen, V.A., Yáñez-Cuna, J.O., Amendola, M., van Ruiten, M.S., Krijger, P.H.L., Teunissen, H., Medema, R.H., van Steensel, B., et al. (2017). The cohesin release factor WAPL restricts chromatin loop extension. Cell *169*, 693–707.e14.

Hadjur, S., Williams, L.M., Ryan, N.K., Cobb, B.S., Sexton, T., Fraser, P., Fisher, A.G., and Merkenschlager, M. (2009). Cohesins form chromosomal cis-interactions at the developmentally regulated IFNG locus. Nature *460*, 410–413.

Heinz, S., Benner, C., Spann, N., Bertolino, E., Lin, Y.C., Laslo, P., Cheng, J.X., Murre, C., Singh, H., and Glass, C.K. (2010). Simple combinations of lineage-determining transcription factors prime cis-regulatory elements required for macrophage and B cell identities. Mol. Cell *38*, 576–589.

Hnisz, D., Abraham, B.J., Lee, T.I., Lau, A., Saint-André, V., Sigova, A.A., Hoke, H.A., and Young, R.A. (2013). Super-enhancers in the control of cell identity and disease. Cell *155*, 934–947.

Hu, B., Petela, N., Kurze, A., Chan, K.L., Chapard, C., and Nasmyth, K. (2015). Biological chromodynamics: a general method for measuring protein occupancy across the genome by calibrating ChIP-seq. Nucleic Acids Res. *43*, e132.

Huber, W., Carey, V.J., Gentleman, R., Anders, S., Carlson, M., Carvalho, B.S., Bravo, H.C., Davis, S., Gatto, L., Girke, T., Gottardo, R., Hahne, F., Hansen, K.D., Irizarry, R.A., Lawrence, M., Love, M.I., MacDonald, J., Obenchain, V., Oleś, A.K., Pagès, H., Reyes, A., Shannon, P., Smyth, G.K., Tenenbaum, D., Waldron, L., and Morgan, M. (2015). Orchestrating high-throughput genomic analysis with Bioconductor. Nat. Meth. *12*, 115–121.

Hughes, J.R., Roberts, N., McGowan, S., Hay, D., Giannoulatou, E., Lynch, M., De Gobbi, M., Taylor, S., Gibbons, R., and Higgs, D.R. (2014). Analysis of hundreds of cis-regulatory landscapes at high resolution in a single, high-throughput experiment. Nat. Genet. *46*, 205–212.

Isono, K., Endo, T.A., Ku, M., Yamada, D., Suzuki, R., Sharif, J., Ishikura, T., Toyoda, T., Bernstein, B.E., and Koseki, H. (2013). SAM domain polymerization links subnuclear clustering of PRC1 to gene silencing. Dev. Cell *26*, 565–577.

Joo, M.S., Koo, J.H., Kim, T.H., Kim, Y.S., and Kim, S.G. (2019). LRH1-driven transcription factor circuitry for hepatocyte identity: Super-enhancer cistromic analysis. EBioMedicine *40*, 488–503.

Joshi, O., Wang, S.Y., Kuznetsova, T., Atlasi, Y., Peng, T., Fabre, P.J., Habibi, E., Shaik, J., Saeed, S., Handoko, L., et al. (2015). Dynamic reorganization of extremely long-range promoter-promoter interactions between two states of pluripotency. Cell Stem Cell *17*, 748–757.

Kent, W.J., Sugnet, C.W., Furey, T.S., Roskin, K.M., Pringle, T.H., Zahler, A.M., and Haussler, D. (2002). The human genome browser at UCSC. Genome Res. *12*, 996–1006.

Kerpedjiev, P., Abdennur, N., Lekschas, F., McCallum, C., Dinkla, K., Strobelt, H., Luber, J.M., Ouellette, S.B., Azhir, A., Kumar, N., Hwang, J., Lee, S., Alver, B.H., Pfister, H., Mirny, L.A., Park, P.J., and Gehlenborg, N. (2018). HiGlass: web-based visual exploration and analysis of genome interaction maps. Genome Biol. *19*, 125.

King, H.W., and Klose, R.J. (2017). The pioneer factor OCT4 requires the chromatin remodeller BRG1 to support gene regulatory element function in mouse embryonic stem cells. eLife *6*, 380.

Knight, P.A., and Ruiz, D. (2012). A fast algorithm for matrix balancing. IMA J. Numer. Anal *33*, 1029–1047.

Kraft, K., Magg, A., Heinrich, V., Riemenschneider, C., Schöpflin, R., Markowski, J., Ibrahim, D.M., Acuna-Hidalgo, R., Despang, A., Andrey, G., et al. (2019). Serial genomic inversions induce tissue-specific architectural stripes, gene misexpression and congenital malformations. Nat. Cell Biol. *21*, 305–310.

Kundu, S., Ji, F., Sunwoo, H., Jain, G., Lee, J.T., Sadreyev, R.I., Dekker, J., and Kingston, R.E. (2017). Polycomb repressive complex 1 generates discrete compacted domains that change during differentiation. Mol. Cell *65*, 432–446.e5.

Langmead, B., and Salzberg, S.L. (2012). Fast gapped-read alignment with Bowtie 2. Nat. Meth. *9*, 357–359.

Langmead, B., Trapnell, C., Pop, M., and Salzberg, S.L. (2009). Ultrafast and memory-efficient alignment of short DNA sequences to the human genome. Genome Biol. *10*, R25.

Lawrence, M., Huber, W., Pagès, H., Aboyoun, P., Carlson, M., Gentleman, R., Morgan, M.T., and Carey, V.J. (2013). Software for computing and annotating genomic ranges. PLoS Comput. Biol. *9*, e1003118.

Lee, T.I., Jenner, R.G., Boyer, L.A., Guenther, M.G., Levine, S.S., Kumar, R.M., Chevalier, B., Johnstone, S.E., Cole, M.F., Isono, K., et al. (2006). Control of developmental regulators by Polycomb in human embryonic stem cells. Cell *125*, 301–313.

Li, H., Handsaker, B., Wysoker, A., Fennell, T., Ruan, J., Homer, N., Marth, G., Abecasis, G., and Durbin, R. (2009). 1000 Genome Project Data Processing Subgroup, 2009. The Sequence Alignment/Map format and SAMtools. Bioinformatics *25*, 2078–2079.

Lieberman-Aiden, E., van Berkum, N.L., Williams, L., Imakaev, M., Ragoczy, T., Telling, A., Amit, I., Lajoie, B.R., Sabo, P.J., Dorschner, M.O., et al. (2009). Comprehensive mapping of long-range interactions reveals folding principles of the human genome. Science *326*, 289–293.

Love, M.I., Huber, W., and Anders, S. (2014). Moderated estimation of fold change and dispersion for RNA-seq data with DESeq2. Genome Biol. *15*, 550.

Lupiáñez, D.G., Kraft, K., Heinrich, V., Krawitz, P., Brancati, F., Klopocki, E., Horn, D., Kayserili, H., Opitz, J.M., Laxova, R., et al. (2015). Disruptions of topological chromatin domains cause pathogenic rewiring of gene-enhancer interactions. Cell *161*, 1012–1025.

Michaelis, C., Ciosk, R., and Nasmyth, K. (1997). Cohesins: chromosomal proteins that prevent premature separation of sister chromatids. Cell *91*, 35–45.

Miron, E., Oldenkamp, R., Pinto, D.M.S., Brown, J.M., Faria, A.R.C., Shaban, H.A., Rhodes, J.D.P., Innocent, C., de Ornellas, S., Buckle, V., and Schermelleh, L. (2019). Chromatin arranges in filaments of blobs with nanoscale functional zonation. bioRxiv. https://doi.org/10.1101/566638.

Nasmyth, K. (2001). Disseminating the genome: joining, resolving, and separating sister chromatids during mitosis and meiosis. Annu. Rev. Genet. *35*, 673–745.

Natsume, T., Kiyomitsu, T., Saga, Y., and Kanemaki, M.T. (2016). Rapid protein depletion in human cells by auxin-inducible degron tagging with short homology donors. Cell Rep. *15*, 210–218.

Nora, E.P., Lajoie, B.R., Schulz, E.G., Giorgetti, L., Okamoto, I., Servant, N., Piolot, T., van Berkum, N.L., Meisig, J., Sedat, J., et al. (2012). Spatial partitioning of the regulatory landscape of the X-inactivation centre. Nature *485*, 381–385.

Nora, E.P., Goloborodko, A., Valton, A.L., Gibcus, J.H., Uebersohn, A., Abdennur, N., Dekker, J., Mirny, L.A., and Bruneau, B.G. (2017). Targeted degradation of CTCF decouples local insulation of chromosome domains from genomic compartmentalization. Cell *169*, 930–944.e22.

Nuebler, J., Fudenberg, G., Imakaev, M., Abdennur, N., and Mirny, L.A. (2018). Chromatin organization by an interplay of loop extrusion and compartmental segregation. Proc. Natl. Acad. Sci. USA *115*, E6697–E6706.

Ollion, J., Cochennec, J., Loll, F., Escudé, C., and Boudier, T. (2013). TANGO: a generic tool for high-throughput 3D image analysis for studying nuclear organization. Bioinformatics *29*, 1840–1841.

Peters, J.M., Tedeschi, A., and Schmitz, J. (2008). The cohesin complex and its roles in chromosome biology. Genes Dev. *22*, 3089–3114.

Plys, A.J., Davis, C.P., Kim, J., Rizki, G., Keenen, M.M., Marr, S.K., and Kingston, R.E. (2019). Phase separation of Polycomb-repressive complex 1 is governed by a charged disordered region of CBX2. Genes Dev. *33*, 799–813.

Quinlan, A.R. (2014). BEDTools: the Swiss-army tool for genome feature analysis. Curr. Protoc. Bioinformatics *47*, 11.12.1–34.

Rada-Iglesias, A., Bajpai, R., Swigut, T., Brugmann, S.A., Flynn, R.A., and Wysocka, J. (2011). A unique chromatin signature uncovers early developmental enhancers in humans. Nature *470*, 279–283.

Ramírez, F., Dündar, F., Diehl, S., Grüning, B.A., and Manke, T. (2014). deepTools: a flexible platform for exploring deep-sequencing data. Nucleic. Acids Res. *42*, W187–W191.

Ramírez, F., Lingg, T., Toscano, S., Lam, K.C., Georgiev, P., Chung, H.R., Lajoie, B.R., de Wit, E., Zhan, Y., de Laat, W., et al. (2015). High-affinity sites form an interaction network to facilitate spreading of the MSL complex across the X chromosome in Drosophila. Mol. Cell *60*, 146–162.

Rao, S.S.P., Huntley, M.H., Durand, N.C., Stamenova, E.K., Bochkov, I.D., Robinson, J.T., Sanborn, A.L., Machol, I., Omer, A.D., Lander, E.S., and Aiden, E.L. (2014). A 3D map of the human genome at kilobase resolution reveals principles of chromatin looping. Cell *159*, 1665–1680.

Rao, S.S.P., Huang, S.C., Glenn St Hilaire, B., Engreitz, J.M., Perez, E.M., Kieffer-Kwon, K.R., Sanborn, A.L., Johnstone, S.E., Bascom, G.D., Bochkov, I.D., et al. (2017). Cohesin loss eliminates all loop domains. Cell *171*, 305–320.e24.

Ren, X., Vincenz, C., and Kerppola, T.K. (2008). Changes in the distributions and dynamics of polycomb repressive complexes during embryonic stem cell differentiation. Mol. Cell. Biol. *28*, 2884–2895.

Rowley, M.J., and Corces, V.G. (2018). Organizational principles of 3D genome architecture. Nat. Rev. Genet. *19*, 789–800.

Sanborn, A.L., Rao, S.S.P., Huang, S.C., Durand, N.C., Huntley, M.H., Jewett, A.I., Bochkov, I.D., Chinnappan, D., Cutkosky, A., Li, J., et al. (2015). Chromatin extrusion explains key features of loop and domain formation in wild-type and engineered genomes. Proc. Natl. Acad. Sci. USA *112*, E6456–E6465.

Schindelin, J., Arganda-Carreras, I., Frise, E., Kaynig, V., Longair, M., Pietzsch, T., Preibisch, S., Rueden, C., Saalfeld, S., Schmid, B., Tinevez, J.-Y., White,

D.J., Hartenstein, V., Eliceiri, K., Tomancak, P., and Cardona, A. (2012). Fiji: an open-source platform for biological-image analysis. Nat. Meth. *9*, 676–682.

Schoenfelder, S., Sugar, R., Dimond, A., Javierre, B.M., Armstrong, H., Mifsud, B., Dimitrova, E., Matheson, L., Tavares-Cadete, F., Furlan-Magaril, M., et al. (2015). Polycomb repressive complex PRC1 spatially constrains the mouse embryonic stem cell genome. Nat. Genet. *47*, 1179–1186.

Schuettengruber, B., Bourbon, H.M., Di Croce, L., and Cavalli, G. (2017). Genome regulation by polycomb and trithorax: 70 years and counting. Cell *171*, 34–57.

Schwarzer, W., Abdennur, N., Goloborodko, A., Pekowska, A., Fudenberg, G., Loe-Mie, Y., Fonseca, N.A., Huber, W., Haering, C.H., Mirny, L., and Spitz, F. (2017). Two independent modes of chromatin organization revealed by cohesin removal. Nature *551*, 51–56.

Tarasov, A., Vilella, A.J., Cuppen, E., Nijman, I.J., and Prins, P. (2015). Sambamba: fast processing of NGS alignment formats. Bioinformatics *31*, 2032–2034.

Taruttis, F., Feist, M., Schwarzfischer, P., Gronwald, W., Kube, D., Spang, R., and Engelmann, J.C. (2017). External calibration with Drosophila whole-cell spike-ins delivers absolute mRNA fold changes from human RNA-Seq and qPCR data. Biotechniques *62*, 53–61.

Tatavosian, R., Kent, S., Brown, K., Yao, T., Duc, H.N., Huynh, T.N., Zhen, C.Y., Ma, B., Wang, H., and Ren, X. (2019). Nuclear condensates of the Polycomb protein chromobox 2 (CBX2) assemble through phase separation. J. Biol. Chem. *294*, 1451–1463.

Wang, X., Brandão, H.B., Le, T.B.K., Laub, M.T., and Rudner, D.Z. (2017). Bacillus subtilis SMC complexes juxtapose chromosome arms as they travel from origin to terminus. Science *355*, 524–527.

Whyte, W.A., Orlando, D.A., Hnisz, D., Abraham, B.J., Lin, C.Y., Kagey, M.H., Rahl, P.B., Lee, T.I., and Young, R.A. (2013). Master transcription factors and mediator establish super-enhancers at key cell identity genes. Cell *153*, 307–319.

Wingett, S., Ewels, P., Furlan-Magaril, M., Nagano, T., Schoenfelder, S., Fraser, P., and Andrews, S. (2015). HiCUP: pipeline for mapping and processing Hi-C data. F1000Res. *4*, 1310.

Wutz, G., Várnai, C., Nagasaka, K., Cisneros, D.A., Stocsits, R.R., Tang, W., Schoenfelder, S., Jessberger, G., Muhar, M., Hossain, M.J., et al. (2017). Topologically associating domains and chromatin loops depend on cohesin and are regulated by CTCF, WAPL, and PDS5 proteins. EMBO J. *36*, 3573–3599.

Zhang, Y., Liu, T., Meyer, C.A., Eeckhoute, J., Johnson, D.S., Bernstein, B.E., Nusbaum, C., Myers, R.M., Brown, M., Li, W., and Liu, X.S. (2008). Model-based analysis of ChIP-Seq (MACS). Genome Biol. *9*, R137–R139.

STAR★METHODS

KEY RESOURCES TABLE

REAGENT or RESOURCE	SOURCE	IDENTIFIER
Antibodies		
anti-RAD21, 1:1000	Abcam	Cat# ab154769; RRID:AB_2783833
anti-RING1B (WB), 1:1000	Atsuta et al., 2001	N/A
anti-RING1B (ChIP), 1:1000	Cell Signaling Technology	Cat# 5694; RRID:AB_10705604
anti-CTCF, 1:1000	Abcam	Cat# ab70303; RRID:AB_1209546
anti-TUBULIN, 1:500	Abcam	Cat# ab6046; RRID:AB_2210370
anti-H3S10p, 1:25	Cell Signaling Technology	Cat# 9706; RRID:AB_331748
anti-mouse-Alexa594, 1:1000	Thermo Fisher Scientific	Cat# ab150116; RRID:AB_2650601
Anti-Digoxigenin-Fluorescein	Roche	Cat# 11207741910; RRID:AB_514498
Fluorescein Rabbit anti-sheep IgG	Vector Laboratories	Cat# FI-6000; RRID:AB_2336218
Chemicals, Peptides, and Recombinant Proteins		
Methanol-free Formaldehyde	Thermo Fisher Scientific	Cat# 10751395
SensiMix SYBR No-ROX Kit	Bioline	Cat# QT650-20
Lipofectamine 3000	Thermo Fisher Scientific	Cat# L3000015
DpnII (Capture-C)	Klose lab, in-house	N/A
T4 DNA ligase, HC 30U/ul (Capture-C)	Thermo Fisher Scientific	Cat# 10548730
Mouse COT-1 DNA	Invitrogen	Cat# 18440-016
Indole-3-acetic acid sodium salt	Sigma Aldrich	Cat# I5148
MboI (Hi-C)	NEB	Cat# R0147L
biotin-14-dATP	Thermo Fisher Scientific	Cat# 19518-018
Dynabeads MyOne Streptavidin C1	Thermo Fisher Scientific	Cat# 65001
Halo-JF549	Gift, Luke D. Lavis and Jonathan B. Grimm (Janelia Research Campus, HHMI)	n/a
Hoechst 33258	Thermo Fisher Scientific	Cat# H3569
PFA	Alfa Aesa	Cat# 43368
DNase I, recombinant	Roche	Cat# 4716728001
DNA polymerase I	NEB	Cat# M0209S
Cy3-dUTP	GE Healthcare	Cat# PA 53022
Digoxygenin-11-dUTP	Roche	Cat# 1073963
HEPES	GIBCO	Cat# 15630056
DAPI	Roche	Cat# 10236276001
Exonuclease III	NEB	Cat# M0206L
Kreatech Hybridization Buffer	Leica	Cat# KBI-FHB
T4 DNA ligase	Thermo Fisher Scientific	Cat# EL0012
T4 DNA polymerase	NEB	Cat# M0203L
RNase A	Applichem	Cat# A3832,0050
Proteinase K solution	Merck Chemicals	Cat# E00012
IGEPAL CA-630	Sigma-Aldrich	Cat# 18896
cOmplete ULTRA Tablets, Mini. EASY Pack	Roche	Cat# 05 892 970 001
Agencourt AMPure XP beads	Beckman Coulter	Cat# A63881
BSA-Molecular Biology Grade (20 mg/ml)	NEB	Cat# B9000S
1-4-Dithiothreitol	Roth	Cat# 6908.1

(*Continued on next page*)

Continued

REAGENT or RESOURCE	SOURCE	IDENTIFIER
Critical Commercial Assays		
NEBNext® Multiplex Oligos for Illumina® (Index Primers Set 1)	NEB	Cat# E7335L
NEBNext® Multiplex Oligos for Illumina® (Index Primers Set 2)	NEB	Cat# E7500L
NEBNext® Ultra DNA Library Prep Kit for Illumina®	NEB	Cat# E7370L
NEBNext® Ultra II Directional RNA Library Prep Kit for Illumina ®	NEB	Cat# E7760L
NEBNext® Ultra II FS DNA Library Prep Kit for Illumina	NEB	Cat# E7805L
NEBNext rRNA Depletion Kit (HumanMouseRat)	NEB	Cat# E6310L
High Sensitivity DNA Kit for Bioanalyzer	Agilent	Cat# 5067-4626
RNA Pico 6000 Kit for Bioanalyzer	Agilent	Cat# 5067-1513
TURBO DNA-free Kit	Thermo Fisher Scientific	Cat# AM1907
NextSeq® 500/550 High Output Kit v2 (150 cycles)	Illumina	Cat# FC-404-2002
NextSeq 500/550 High Output v2 Kit (75 cycles)	Illumina	Cat# FC-404-2005
KAPA Illumina DNA Standards	Roche	Cat# 7960387001
ChIP DNA Clean and Concentrator	Zymo Research	Cat# D5205
SeqCap EZ Hybridization and Wash Kit	Roche (Nimblegen)	Cat# 5634261001
SeqCap EZ Accessory Kit v2	Roche (Nimblegen)	Cat# 07145594001
SeqCap EZ HE-Oligo Kit A	Roche (Nimblegen)	Cat# 6777287001
SeqCap EZ HE-Oligo Kit B	Roche (Nimblegen)	Cat# 6777317001
NEBNext® End Repair Module	NEB	Cat# E6050L
NEBNext® dA-Tailing Module	NEB	Cat# E6053L
NEBNext® Quick Ligation Module	NEB	Cat# E6056L
Dynabeads® M-270 Streptavidin	Thermo Fisher Scientific	Cat# 65306
Herculase II Fusion DNA Polymerases	Agilent	Cat# 600677
illustra G-50 microspin columns	GE Healthcare	Cat# 27533002
NEBNext® Q5® Hot Start HiFi PCR Master Mix	NEB	Cat# M0543L
Deposited Data		
Capture-C	This study	ArrayExpress: E-MTAB-7840
calibrated ChIP-Seq	This study	ArrayExpress: E-MTAB-7817
calibrated RNA-Seq	This study	ArrayExpress: E-MTAB-7818
Hi-C	This study	ArrayExpress: E-MTAB-7816
Experimental Models: Cell Lines		
Mouse ESC: *Scc1*-mAID-eGFP	This study	N/A
Mouse ESC: AID-*Ring1b*;*Ring1a*−/−	This study	N/A
Mouse ESC: *Scc1*-mAID-eGFP;*Ring1b*-AID;*Ring1a*−/−	This study	N/A
Mouse ESC: *Ctcf.*-AID	Nora et al., 2017	N/A
Mouse ESC: *Scc1*-mAID-eGFP;*Pcgf2*-HaloTag	This study	N/A
Oligonucleotides		
CaptureC probes	This study	See Table S2
gRNA ROSA26 CGCCCATCTTCTAGAAAGAC	This study	N/A
gRNA SCC1 3′ CCACGGTTCCATATTATCTG	This study	N/A
gRNA RING1A 5′UTR CTCAGCGGAGCCCCGCTTGG	This study	N/A
gRNA RING1A Intron 3 GCGACCGTGCAGCTGACGTT	This study	N/A
gRNA RING1B 5′ GCACAGCCTGAGACATTTCT	This study	N/A
gRNA PCGF2 3′ CCCTTTCCTCAAGGGGGGCA	This study	N/A

(*Continued on next page*)

Continued

REAGENT or RESOURCE	SOURCE	IDENTIFIER
Software and Algorithms		
SAMtools (v1.7)	Li et al., 2009	http://www.htslib.org/
Bowtie 2 (v2.3.4)	Langmead and Salzberg, 2012	http://bowtie-bio.sourceforge.net/bowtie2/index.shtml
Sambamba (v0.6.7)	Tarasov et al., 2015	http://lomereiter.github.io/sambamba/
deepTools (v3.0.1)	Ramírez et al., 2014	https://deeptools.readthedocs.io/en/develop/
STAR (v2.5.4)	Dobin et al., 2013	https://github.com/alexdobin/STAR
MACS2 (v2.1.1)	Zhang et al., 2008	https://github.com/taoliu/MACS/tree/master/MACS2
UCSC Genome Browser	Kent et al., 2002	https://genome.ucsc.edu/
Bioconductor (v3.6)	Huber et al., 2015	https://www.bioconductor.org/
DESeq2	Love et al., 2014	https://bioconductor.org/packages/release/bioc/html/DESeq2.html
HOMER	Heinz et al., 2010	http://homer.ucsd.edu/homer/
Chicago (v1.0.4)	Cairns et al., 2016	https://bioconductor.org/packages/release/bioc/html/Chicago.html
HiCUP (v0.5.7)	Wingett et al., 2015	https://www.bioinformatics.babraham.ac.uk/projects/hicup/
IRanges	Lawrence et al., 2013	https://bioconductor.org/packages/release/bioc/html/IRanges.html
BEDtools (v2.17.0)	Quinlan, 2014	https://bedtools.readthedocs.io/en/latest/
GenomicFeatures	Lawrence et al., 2013	https://bioconductor.org/packages/release/bioc/html/GenomicFeatures.html
GenomicRanges	Lawrence et al., 2013	https://bioconductor.org/packages/release/bioc/html/GenomicRanges.html
ImageJ	Schindelin et al., 2012	https://imagej.net/Welcome
TANGO in ImageJ	Ollion et al., 2013	https://biophysique.mnhn.fr/tango/HomePage
biopython (v1.73) “Restriction” module	Cock et al., 2009	https://biopython.org/
HiCCUPs	Rao et al., 2014	https://github.com/aidenlab/juicer/wiki/HiCCUPS
scipy (v1.3.1)		https://www.scipy.org/
cooler (v0.8.5)	Abdennur and Mirny, 2019	https://github.com/mirnylab/cooler
higlass-manage (v0.7.3)	Kerpedjiev et al., 2018	https://github.com/higlass/higlass-manage
coolpup.py (v0.8.7)	Flyamer et al., 2019	https://github.com/Phlya/coolpuppy

LEAD CONTACT AND MATERIALS AVAILABILITY

Further information and requests for resources and reagents should be directed to and will be fulfilled by the Lead Contact, Rob Klose (rob.klose@bioch.ox.ac.uk). All unique/stable reagents generated in this study are available from the Lead Contact with a completed Materials Transfer Agreement.

EXPERIMENTAL MODEL AND SUBJECT DETAILS

Cell Culture

All cell lines were generated from a wild-type male E14 mouse ESCs cell background. ESCs were grown on gelatin-coated plates in DMEM supplemented with 10% FBS, penicillin/streptomycin, 5 μM 2-Mercaptoethanol, 2 mM L-glutamine, non-essential amino-acids and 10 ng/mL recombinant Leukaemia-Inhibitory Factor (LIF).

Protein Degradation and Nocodazole treatment

ESCs were plated on 10 cm dishes one day before treatment. Medium was replaced with equilibrated (37°C and 5% CO_2) medium containing either auxin sodium salt (500 μM) (Sigma) or 100 ng/ml nocodazole (Sigma). The cells were incubated for 6 h or 48h (CTCF-AID) before trypsinisation and cell counting (in auxin containing medium). 100,000 cells were used for Hi-C and the rest for western blotting to confirm protein degradation.

METHOD DETAILS

Generation, culturing and treatment of cell lines

Cloning

pSpCas9(BB)-2A-Puro (PX459) was used to construct CRISPR/Cas9 vectors (Addgene 48139). The following gRNA oligos were cloned into the BbsI restriction site:

ROSA26 CGCCCATCTTCTAGAAAGAC
SCC1 3′ CCACGGTTCCATATTATCTG
RING1A 5′UTR CTCAGCGGAGCCCCGCTTGG
RING1A Intron 3 GCGACCGTGCAGCTGACGTT
RING1B 5′ GCACAGCCTGAGACATTTCT
PCGF2 3′ CCCTTTCCTCAAGGGGGGCA

For homology directed gene targeting and repair 500-1000 bp homology arms were generated by Gibson Assembly.

Gene Editing

The coding sequence for *Oryza sativa* TIR1 and a splice acceptor was heterozygously introduced into the ROSA26 locus by cotransfection of pX459 ROSA26 and pUC19 ROSA-TIR1. The resulting ESCs were ROSA-TIR1.

The mini-AID and eGFP was introduced at the C terminus of SCC1 in ROSA-TIR1 ESCs by cotransfection of pX459 SCC1 3′ and pUC19 SCC1-mAID-eGFP. The resulting ESCs were ROSA-TIR1 SCC1-mAID-eGFP (SCC1-AID).

RING1A was deleted from ROSA-TIR1 ESCs by cotransfection of two gRNAs spanning exons 1 to 3 (pX459 RING1A 5′ UTR and pX459 RING1A Intron 3). The resulting ESCs were ROSA-TIR1 RING1AΔ.

Full length AID was introduced at the N terminus of RING1B in ROSA-TIR1 RING1AΔ ESCs by cotransfection of pX459 RING1B 5′ and pUC19 AID-RING1B. The resulting ESCs were ROSA-TIR1 RING1AΔ AID-RING1B (RING1B-AID).

The mini-AID and GFP was introduced at the C terminus of SCC1 in ROSA-TIR1 RING1AΔ AID-RING1B ESCs by cotransfection of pX459 SCC1 3′ and pUC19 SCC1-mAID-GFP. The resulting ESCs were ROSA-TIR1 RING1AΔ AID-RING1B SCC1-mAID-GFP (SCC1-AID RING1B-AID).

The HaloTag was introduced at the C terminus of PCGF2 in SCC1-mAID-eGFP ESCs by cotransfection pX459 PCGF2 3′ and pUC19 PCGF2-HT. Transfected cells were labeled with 500 nM Halo-TMR and FACS-selected to identify clones with functional HaloTagCells were transfected using lipofectamine 2000. The next day cells were passaged and transfected cells were selected with puromycin (1 μg/ml) for two days. Eight days after puromycin removal, colonies were picked and genotyped by PCR and western blotting.

In situ Hi-C library generation for low cell input

We performed *in situ* Hi-C on control (TIR1+Auxin), SCC1^{DEG} (SCC1-AID+Auxin), RING1B^{DEG} (RING1B-AID+Auxin) ESCs (Díaz et al., 2018) in biological duplicates. 100,000 ESCs were crosslinked in 1% formaldehyde and incubated for 10 min at room temperature with rotation (20 rpm). The reaction was quenched by adding glycine (0.2 M) and incubating for 5 min at room temperature with gentle rotation (20 rpm). Cells were washed three times with 1 mL of cold PBS (centrifuged at 300 g for 5 min at 4°C) and then gently resuspended in 250 μL of ice-cold *in situ* Hi-C buffer (10 mM Tris-Cl pH 8.0, 10 mM NaCl, 0.2% IGEPAL CA-630, cOmplete Ultra protease inhibitors) and incubated on ice for 15 min. Samples were then centrifuged and resuspended in 250 μl of *in situ* Hi-C buffer. Cells were centrifuged (13,000 g for 5 min at 4°C) and resuspended in 250 μl ice-cold 10x NEB2 buffer. Nuclei were centrifuged (13,000 g for 5 min at 4°C) and permeabilised by resuspending them in 50 μl of 0.4% SDS and incubating at 65°C for 10 min. SDS was quenched by adding 25 μl of 10% Triton X-100 and 145 μl of nuclease-free water and incubated at 37°C for 45 min with shaking (650 rpm). Chromatin was digested by adding 100 U of MboI in 20 μl of 10x NEB2.1 buffer for 90 min at 37°C with rotation. MboI was heat-inactivated at 62°C for 20 min. The overhangs generated by the restriction enzyme were filled-in by adding a mix of 0.4 mM biotin-14-dCTP (Thermo Fisher Scientific), 10 mM dATP/dGTP/dTTP (0.75 μl of each dinucleotide), and 5 U/μl DNA polymerase I Klenow (8 μl; New England Biolabs), and incubated for 90 min at 37°C with rotation. DNA fragments were ligated in nuclease-free water (657 μl), 10x T4 DNA ligase buffer (120 μl), 10% Triton X-100 (100 μl), 20 mg/mL BSA (12 μl) and 5 Weiss U/μl T4 DNA ligase (5 μl in two instalments; Thermo Fisher Scientific) by incubating 4 h at 20°C with gentle rotation. Nuclei were centrifuged (2,500 g for 5 min at room temperature) and resuspended in 500 μl extraction buffer. Protein was digested with 20 μl of 20 mg/mL Proteinase K (Applichem), for 30 min at 55°C with shaking (1,000 rpm). 130 μL of 5M NaCl was added followed by overnight incubation at 65°C with shaking (1,000 rpm). Phenol-Chloroform-Isoamyl alcohol (25:24:1; Sigma-aldrich) extracted DNA was resuspended in 30 μl

of 10mM Tris pH 8.0 (Applichem) and incubated for 15 min at 37°C with 10 mg/ml RNase A (1 μl; Applichem). In order to remove biotin from unligated fragments, DNA samples were incubated at 20°C for 4h without rotation in a mix of 10 μl of 10x NEB2 buffer (New England Biolabs), 1 mM of a dNTPs mix (10 μl), 20 mg/mL BSA (0.5 μl), 3 U/μl T4 DNA polymerase (5 μl; New England Biolabs) and nuclease-free water (up to 100 μl). Samples were sheared using a Covaris S220 instrument (2 cycles, each 50 s, 10% duty, 4 intensity, 200 cycles/burst). Biotinylated fragments were pulled down using Dynabeads MyOne Streptavidin C1 beads. Libraries were end repaired on beads using the NEBNext Ultra End Repair module (New England Biolabs) and washed twice on 1x B&W (10 mM Tris-Cl pH 7.4, 1 mM EDTA, 2 M NaCl) + 0.1% Triton X-100, resuspended in 50 μl and transferred to a 1.5 mL tube. Adaptors for Illumina sequencing was added using the NEBNext® Ultra dA-Tailing module (New England Biolabs). Final amplification of the libraries was done in 4 parallel reactions per sample as follows: 10 μl of the bead-bound libraries, 25 μl of 2x NEBNext Ultra II Q5 Master Mix, 5 μl of 10 μM Universal PCR primer, 5 μl of 10 μM Indexed PCR primer and 10 μl of nuclease-free water.

Samples were individually barcoded and amplified for 10 (Tir1+Aux_Batch1, Ring1B+Aux_Batch1, Scc1+Aux_Batch1), 12 (Ring1B+Aux_Batch3) or 14 (Tir1+Aux_Batch3, Scc1+Aux_Batch3) cycles following the program: 98°C for 1 min, (98°C for 10 s, 65°C for 75 s, ramping 1.50°C/s) repeated 10-14 times, 65°C for 5 min, 4°C hold.

The four reactions were combined into one tube and size-selected using Ampure XP beads (Beckman Coulter). Final Hi-C libraries were quantified using Qubit dsDNA HS assay kit and a DNA HS kit on a 2100 Bioanalyzer (Agilent). Libraries were first pooled and shallow sequenced on an Illumina MiSeq (2x84bp paired-end; MiSeq reagent kit v3-150 cycles) to assess library quality. They were then sequenced on an Illumina NextSeq (2x80 bp paired-end; NextSeq 500/550 High Output kit v2-150 cycles).

Capture-C

Capture-C library generation

Capture-C libraries were prepared as described previously (Davies et al., 2016). 10^7 mouse ES cells were trypsinized, collected in 50ml falcon tubes in 9.3ml media and crosslinked with 1.25 mL 16% formaldehyde for 10 min at room temperature. Cells were quenched with 1.5ml 1 M glycine, washed with PBS and lysed for 20 min at 4°C while rotating (lysis buffer: 10 mM Tris pH 8, 10 mM NaCl, 0.2% NP-40, supplemented with complete proteinase inhibitors) prior to snap freezing in 1 mL lysis buffer at −80°C. Lysates were then thawed on ice, pelleted and resuspended in 650 μl 1x DpnII buffer (NEB). Three 1.5ml tubes with 200 μl lysate each were treated in parallel with SDS (0.28% final concentration, 1 h, 37°C, interval shaking 500rpm, 30 s on/30 s off), quenched with trypsin (1.67%, 1h at 37°C, interval shaking 500rpm, 30 s on/30sec off) and subjected to a 24 h digestion with 3x10 μl recombinant DpnII (37°C, interval shaking 500rpm, 30 s on/30 s off). Each chromatin aliquot was independently ligated with 8 μl T4 Ligase (240 U) in a volume of 1440 μl (20 h at 16°C). Following this, the nuclei containing ligated chromatin were pelleted, reverse-crosslinked and the ligated DNA was phenol-chloroform purified. The sample was resuspended in 300 μl water and sonicated 13x (Bioruptor Pico, 30 s on, 30 s off) or until a fragment size of approximately 200 bp was reached. Fragments were size selected using AmpureX beads (Beckman Coulter, selection ratios: 0.85x / 0.4x) and the correct size was assessed by Bioanalyzer. 2x 1-5 μg of DNA were adaptor ligated and indexed using the NEBNext DNA library Prep Reagent Set (New England Biolabs: E6040S/L) and NEBNext Multiplex Oligos for Illumina Primer sets 1 (New England) and 2 (New England). The libraries were amplified 7x using Herculase II Fusion Polymerase kit (Agilent).

Capture-C hybridization and sequencing

5′ biotinylated probes (see Table S2) were designed using the online tool by the Hughes lab (CapSequm, http://apps.molbiol.ox.ac.uk/CaptureC/cgi-bin/CapSequm.cgi) to be 70-120bp long and two probes for each promoter of interest. The probes were pooled at 2.9nM each. Samples were captured twice and hybridizations were carried out for 72h and for 24h for the first and the second captures, respectively. To even out capture differences between tubes, libraries were pooled prior to hybridization. For Control, $SCC1^{DEG}$, $RING1B^{DEG}$ and $SCC1^{DEG}$ $RING1B^{DEG}$, 1.5μg of each replicate was individually hybridized and then pooled for the second round of hybridization. CTCF ± AUX were multiplexed prior to the first capture at 2 μg each. Hybridization was carried out using Nimblegen SeqCap (Roche, Nimblegen SeqCap EZ HE-oligo kit A, Nimblegen SeqCap EZ HE-oligo kit B, Nimblegen SeqCap EZ Accessory kit v2, Nimblegen SeqCap EZ Hybridization and wash kit) following manufacturer's instructions for 72 h followed by a 24 h hybridization (double Capture). The captured library molarity was quantified by qPCR using SensiMix SYBR (Bioline, UK) and KAPA Illumina DNA standards (Roche) and sequenced on Illumina NextSeq 500 platform for three biological replicates.

Calibrated RNA-seq and ChIP-seq

Calibrated total RNA-seq (cRNA-seq)

To prepare RNA for cRNA-seq, 5 million mouse ESCs (SCC1-AID ± Auxin) were mixed with 2 million *Drosophila* SG4 cells. Total RNA was extracted using RNeasy Mini Kit (QIAGEN) according to the manufacturer's protocol, followed by treatment with the TURBO DNA-free Kit (ThermoScientific). Quality of RNA was assessed using 2100 Bioanalyzer RNA 6000 Pico kit (Agilent). To construct libraries, for each sample RNA was first depleted of rRNA using the NEBNext rRNA Depletion kit (NEB). RNA-seq libraries were then prepared from 200 ng of RNA using the NEBNext Ultra II Directional RNA-seq kit (NEB). To quantify the consistency of spike-in cell mixing for each individual sample, genomic DNA was isolated from a small aliquot of mixed mouse and fly cells using Quick-DNA Miniprep kit (Zymo Research) according to the manufacturer's protocol. Libraries from 50 ng of genomic DNA were constructed using NEBNext Ultra II FS DNA Library Prep Kit (NEB), following manufacturer's guidelines. NEBNext Multiplex Oligos were used for indexing libraries. The average size of all libraries was analyzed using the 2100 Bioanalyzer High Sensitivity DNA Kit (Agilent)

and the libraries concentration was measured by qPCR using SensiMix SYBR (Bioline, UK) and KAPA Illumina DNA standards (Roche). cRNA-seq and gDNA-seq libraries were sequenced as 80 bp paired-end reads on the Illumina NextSeq 500 platform for four independent biological replicates.

Calibrated ChIP-seq

50 million Control or SCC1^{DEG} mESCs were mixed with 500,000 HEK293 cells before fixation. Cells were fixed for 10 minutes in 1% formaldehyde at room temperature. Formaldehyde was quenched by the addition of glycine to a final concentration of 125 μM. All subsequent steps were as previously described (King and Klose, 2017). Libraries were sequenced for three biological replicates.

FISH

RASER (Resolution After Single-strand Exonuclease Resection)-FISH

RASER-FISH was conducted as previously described (Brown et al., 2018) with minor changes. Briefly, cells were grown on coverslips, labeled for 24 h with BrdU/BrdC mix (3:1) at final conc. of 10 μM, with auxin added at 500 μM for the final 6 h. Cells were fixed in 4% PFA (vol/vol) for 15 min and permeabilised in 0.2% Triton X-100 (vol/vol) for 10 min. Cells were then stained with DAPI (0.5 μg/mL in PBS), exposed to 254 nm wavelength UV light for 15 min, then treated with Exonuclease III (NEB) at 5 U/μL at 37°C for 15 min. Labeled probes (100 ng each) were denatured in hybridization mix at 90°C for 5 min and pre-annealed at 37°C for 10 min. Coverslips were hybridized with prepared probes at 37°C overnight. Following hybridization, coverslips were washed for 30 min twice in 2x SSC at 37°C, once in 1xSSC at RT. Coverslips were blocked in 3% BSA (wt/vol) and digoxigenin was detected with sheep anti-digoxigenin FITC 1/50 (Roche, 11207741910) followed by rabbit anti–sheep FITC 1/100 (Vector Laboratories, FI-6000). Coverslips were stained with DAPI (0.5 μg/mL in PBS), washed with PBS and mounted Vectashield (Vector Laboratories).

Probes and nick-translation labeling

Fosmid probes WIBR1-0935O10 (Nkx2.3 mm9; chr19; 43,659,682-43,698,592), WIBR1-1122P14 (Pax2 mm9; chr19; 44,809,035-44,851,675), WIBR1-1125H10 (Dlx2 mm9; chr2: 71374041-71411685), WIBR1-2777G14 (HoxD10 mm9; chr2: 74511607-74550498) were obtained from BACPAC Resources Center (Children's Hospital Oakland Research Institute; [https://bacpacresources.org/]). Probes were labeled for use in FISH by nick translation as follows: prior to nick translation, 1 μg DNA was treated with RNase (0.02 U) (Sigma), for 30 min at 37°C, nick translation was carried out at 16°C for 1 h in the following reaction mixture; 50 mM Tris-HCl, 5 mM $MgCl_2$, 2.5 μg BSA, 10 mM β-mercaptoethanol, 50 mM dAGC, 20 μM hapten/fluor [digoxigenin-11-dUTP (Sigma); Cy3 dUTP (GE Healthcare)], 15 U recombinant DNase1 (Sigma) and 10 U DNA polymerase I (NEB), made up to a final volume of 50 μL with H_20.

Imaging Equipment and Settings

Widefield fluorescence imaging was performed at 20°C on a DeltaVision Elite system (Applied Precision) equipped with a 100x/1.40 NA UPLSAPO oil immersion objective (Olympus), a CoolSnap HQ2 CCD camera (Photometrics), DAPI (excitation 390/18; emission 435/40), FITC (excitation 475/28; emission 525/45) and TRITC (excitation 542/27; emission 593/45) filters. 12-bit image stacks were acquired with a z-step of 150 nm giving a voxel size of 64.5 nm x 64.5 nm x 150 nm. Image restoration was carried out using Huygens deconvolution Classic Maximum Likelihood Estimation (Scientific Volume Imaging B.V.).

smRNA-FISH

Single molecule RNA fluorescence *in situ* hybridization (smRNA-FISH) has been performed following standard approaches (Stellaris). Briefly, the ESCs growing in colonies on 10 cm culture plates for 48h were treated with auxin and harvested using trypsinisation, fixed in 4% formaldehyde for 10 mins and permeabilised for minimum 30 min in 70% ethanol. Cells were centrifuged, resuspended and transferred to hybridization mixture comprising 20% Dextran Sulfate, 10% formamide, 2x SSC with probes (Q670) specific to introns of the indicated genes in a total volume of 200 μl. After over-night incubation at 37°C, cells were washed multiple times with hybridization buffer without Dextran Sulfate, 2x SSC, stained with DAPI (DNA) and Agglutinin-A488 (cell membranes) mixed with Vectashield (Vectorlabs) and plated on a coverslip to form a cell mono-layer. Images were acquired using an Olympus IX-83 system running Cell Sens software, equipped with 63x 1.4 NA oil objective lens and 1200x1200px2 sCMOS camera (Photometrics), 91.5 nm pixel size. Image stacks comprised 30 images in total and were acquired with 350 nm Z-interval.

Characterization of Polycomb bodies

Polycomb body imaging

To image Polycomb bodies in live cells, PCGF2-HaloTag SCC1^{DEG} cells were plated on a gelatinised 35 mm Petri dish, 14 mm Microwell 1.5 coverglass dishes (MatTek) at least 5 hours prior to imaging. Cells were labeled with 500 nm Halo-JF549 (Grimm et al., 2015) for 15 min at 37°C, followed by three washes, changing medium to Fluorobrite DMEM (Thermo Fisher Scientific) for imaging, which was supplemented as described for general ESC culture above. Cells were incubated for a further 30 min in supplemented Fluorobrite DMEM with 10 μg/mL Hoechst 33258 (Thermo Fisher Scientific) at 37°C and washed once more before imaging. Imaging was performed with an IX81 Olympus microscope connected to a Spinning Disk Confocal system (UltraView VoX PerkinElmer) using an EMCCD camera (ImagEM, Hamamatsu Photonics) in a 37°C heated, humidified, CO_2-controlled chamber. Z stacks were acquired using a PlanApo 100x/1.4 N.A. oil-immersion objective heated to 37°C, using Volocity software (PerkinElmer). PCGF2-HaloTag-JF549 was imaged with a 561 nm laser at 1.25 s exposure at 15% laser power, SCC1-AID-GFP with a 488 nm laser at

1 s exposure at 40% laser power, while Hoechst was imaged with a 405 nm laser at 250 ms exposure at 20% laser power. Z stacks were acquired at 150 nm intervals. A total of at least 28 cells were imaged per condition in two biological replicates.

FACS

Cells were trypsinised and fixed for 15 min in 4% formaldehyde in PBS. Following two washes in PBS the fixed cells were permeabilized in 90% methanol. Three washes were performed in PBS followed by a 1 h incubation in anti-H3S10p primary antibody diluted in 5% bovine serum albumin in PBS (PBS-BSA). The cells were then washed in PBS three times and resuspended in anti-mouse-Alexa594 secondary antibody in PBS-BSA. DNA was stained with a 10 min incubation in 1 μg/ml DAPI. DAPI and H3S10p immunofluorescence intensity was measured by FACS using a BD Calibur cell sorter.

Antibodies

RAD21, 1:1000 (Abcam, ab154769), RING1B (western) 1:1000 (Klose Lab), RING1B (ChIP-seq) 1:1000 (Cell Signaling, 5694), CTCF 1:1000 (Abcam, ab70303) and TUBULIN 1:500 (Abcam, ab6046), H3S10p 1:25 (Cell Signaling, 9706), anti-mouse-Alexa594 1:1000 (Thermo, ab150116)

QUANTIFICATION AND STATISTICAL ANALYSIS

Hi-C

Hi-C data analysis

For each library, paired-end reads were independently mapped against the mm10 reference genome (UCSC) using Bowtie2 in '–very-sensitive' mode. Unmapped reads were truncated by 8bp and realigned iteratively, until a valid alignment could be found or the truncated read was shorter than 30bp. Only uniquely mapping reads with a mapping quality (MAPQ) > = 30 were kept in the downstream analysis. Biopython "Restriction" module was then used to compute predicted restriction fragments. Uniquely mapped reads were assigned to fragments, fragments to pairs and pairs filtered for self-ligated fragments, PCR duplicates, read pairs mapping further than 5 kb from the nearest restriction site, and for uninformative ligation products (Cournac et al., 2012). The genome was binned at 10 kb resolution, and Hi-C matrices were built by counting the number of valid fragment pairs per bin. Bins with less than 10% of the median number of fragments per bin were masked before the matrix was normalized using KR matrix balancing per chromosome (Knight and Ruiz, 2012).

Observed/expected (OE) Hi-C matrix generation

Expected Hi-C contact values were obtained by calculating the average contact intensity for all loci with the same distance. The normalized Hi-C matrix is then transformed into an observed/expected (O/E) matrix by dividing each normalized observed by its corresponding expected value at that distance. O/E matrix generation was performed for each chromosome separately.

A/B compartment quantification

A/B compartment calculation was done following a previously described procedure (Lieberman-Aiden et al., 2009; Flyamer et al., 2017). Briefly, O/E matrices for each chromosome at 500 kb resolution were transformed into a correlation matrix by calculating the Pearson correlation of row i and column j for each (i, j). The first eigenvector of the correlation matrix forms the compartment vector. To ensure that positive values indicate the A (active) compartment and negative values the B (inactive) compartment, we used GC content as a proxy: if the average GC content of regions with negative entries is higher than that of regions with positive entries, the eigenvector sign is inverted. Absolute intra-chromosomal correlation values were compared between conditions as a measurement of compartmentalisation.

Hi-C peak calling

SCC1-AID peaks were called in 100kb resolution matrices using an in-house, CPU implementation of HiCCUPs (Rao et al., 2014). Enrichment and FDR values for each pixel were obtained as described (Rao et al., 2014). Peaks must (i) have a minimum of 2.25-fold enrichment over the donut neighborhood, (ii) have an FDR $\leq$ 0.05 in the donut neighborhood, (iii) have an FDR $\leq$ 0.1 in the remaining the neighborhoods, and (iv) have a minimum observed value of 29 contacts in the peak center. The robustness of these specific values has been confirmed visually for a large number of regions in order to minimize false-positives.

Aggregate Hi-C feature analysis (TADs, peaks and A/B compartments)

Published ESC TAD intervals were used for aggregate TAD analysis (Bonev et al., 2017). For calculating the aggregate TAD plots, subsets of the O/E matrices were extracted and averaged to obtain the output sub-matrices. Sub-matrices of different sizes were interpolated using "imresize" with the "nearest" setting from the Scipy Python package. Using the TAD and peak calls for each of the groups (see the above section "Average Hi-C feature analysis" for parameter details). The aggregate analysis of the O/E matrices were calculated at 10kb resolution for TADs (Flyamer et al., 2017) and at 10 kb resolution for peaks. For calculating the aggregate peak analyses, we used coolpup.py (Flyamer et al., 2019) on 10kb resolution unbalanced matrices over 500kb windows. *Cis* interactions between polycomb domains and superenhancers separated by at least 250kb were compared against a set of 100 shifted regions per interaction to calculate enrichment scores and remove coverage- and distance-dependent- artifacts. Chromosomes X, Y and MT were excluded from these analyses. The trimmed mean of the enrichment scores at the center 50kb of the aggregated matrix is displayed as a value of enrichment. Similar results were obtained using observed versus expected enrichment tests and iteratively corrected matrices.

ChIP-seq read enrichment quantitation at Hi-C peaks

Datasets in Table S1 were processed using the standard pipeline in the lab (see cChIP-seq read processing below). Pileups were built using MACS2 and the obtained bedgraph files were used to quantify read count enrichments. Read count enrichments were quantified separately for source and sink of each interaction using the function annotatePeaks.pl from HOMER (Heinz et al., 2010) with the options –size given -raw. For each peak, an average enrichment was quantified using the mean between source and sink. This was repeated for 1000 distance- and chromosome-matched random source-sink pairs. Fold enrichment was quantified by dividing observed enrichment by the mean enrichment at random source-sink pairs.

Intervals for Aggregate Peak Analysis

Polycomb domains

Polycomb domains for mESCs (n = 2096) were defined as previously described (Fursova et al., 2019). For other cell lines, we used publically available datasets to define polycomb domains. All peaks were called using the macs2 callpeak function from MACS2.

For HCT116 we first called H3K27me3 ChIP-seq peaks (GSM2809625, Rao et al., 2017) with the options –q 0.000001 and–broad-cutoff 0.01. To define polycomb domains (n = 517)), H3K27me3 peaks with a distance of less than 20kb were merged using the reduce() function from the R package IRanges (Lawrence et al., 2013) and then overlapped with EZH2 peaks (GSM3498250, ENCODE Project Consortium, 2012) (-q = 0.1,–broad-cutoff = 0.1).

For HAP1 we first called H3K27me3 (rep1) and H2AK119ub (rep2) ChIP-seq peaks (GSE110143; Campagne et al., 2019) with the options –q 0.000001 and–broad-cutoff 0.01. To define polycomb domains (n = 1797), for both datasets ChIP-seq peaks with a distance of less than 10kb were merged using the reduce() function from the R package IRanges (Lawrence et al., 2013) and overlapping ranges of both peak sets were combined to a single range using the reduce() function.

For HeLa we first called H3K27me3 ChIP-seq peaks (GSM733696, ENCODE Project Consortium, 2012) with the options –q 0.01 and–broad-cutoff 0.1. To obtain H3K27me3 overlapping intervals, for each replicate, H3K27me3 peaks with a distance of less than 20kb were merged using the reduce() function from the R package IRanges (Lawrence et al., 2013) and then combined to a single range, again merging peaks at a distance of less than 10kb. We then called EZH2 peaks (GSM1003520, ENCODE Project Consortium, 2012) with the options –q 0.000001 and–broad-cutoff 0.01. To obtain EZH2 overlapping intervals, EZH2 peaks from both replicates were combined to a single range, and ranges at a distance of less than 1kb were merged using the reduce() function. To define polycomb domains (n = 1386)), overlapping H3K27me3 and EZH2 intervals were combined to a single range and further filtered for a size of over 2kb.

For adult mouse liver we first called H3K27me3 ChIP-seq peaks (GSM769034) with the options –q 0.000001 and–broad-cutoff 0.01. To obtain H3K27me3 overlapping intervals, for each replicate, H3K27me3 peaks with a distance of less than 20kb were merged using the reduce() function from the R package IRanges (Lawrence et al., 2013) and then combined to a single range, again merging peaks at a distance of less than 10kb and further filtering for a size of over 2kb to obtain polycomb domains (n = 1370).

Super-enhancers

Super-enhancer intervals were downloaded from the following publications: Whyte et al. (2013) (mESC), Hnisz et al. (2013) (HeLa, HCT116) and Joo et al. (2019) (hepatocytes) and lifted over to mm10 and hg38 as appropriate.

Distance-matched A and B compartments

TADs and their A and B compartment identity were downloaded from (Bonev et al., 2017). Same number of A and B compartments homotypic pairs were selected as the number of loops called in SCC1^{DEG} cell line. To make the data analysis more comparable, the control 100kb regions of a TAD within a respective compartment were selected in a way that allowed two paired 100kb regions to be distance- and chromosome-matched to SCC1^{DEG} called loops.

Capture-C

Capture-C data analysis

Fastq files were aligned to mm10 genome and filtered using HiCUP (v0.5.7) (Wingett et al., 2015) and Bowtie 2 (Langmead et al., 2009) with the settings of 100bp-800bp for fragment sizes. Paired bam files were then processed using the Bioconductor package Chicago (Cairns et al., 2016) (Version: 1.0.4) according to the Chicago Vignette using the inbuilt mESC-2reps weight settings. Interaction “peaks” were called based on Chicago scores > = 5 and interaction peaks closer than 10 fragments in distance were combined to one peak. Weighted average read counts were extracted from the ChicagoData objects. For visualization in line plots, for each DpnII fragment, percentage reads per promoter (PRPP) was calculated for each sample to normalize the read counts. Briefly, read counts were divided by the total coverage of reads aligned to captured promoters in the sample, multiplied by the amount of promoters captured and then multiplied by 100 to obtain % reads per promoter captured for each DpnII restriction fragment (PRPP = N / cov * nprom * 100). For display purposes reads were then multiplied by 1000 for Figures 3, 4, and 5 (mPRPP). For aggregate peak analysis (Figures 3 and 4) significantly enriched interactions were determined using Chicago default threshold of score > = 5 at the level of individual DpnII fragments. Because peaks between polycomb occupied sites are larger than the average DpnII fragment, interactions with < 10 DpnII fragments distance were merged to one peak. Peak summits were then defined as the local maximum in the Control sample (if peaks were present in this sample) or in the sample in which they were present. In order to

make interactions at different distances comparable, all samples were then normalized to PRPPs at the peak summit in Control. For aggregate analyses in Figures 3 and 4 only interactions between polycomb target gene promoters and a stringent set of RING1B peaks (Fursova et al., 2019) were considered.

Calibrated RNA-seq (cRNA-seq) and ChIP-seq (cChIP-seq)

Data processing and normalization

For cRNA-seq, to filter out reads mapping to rDNA fragments, paired-end reads were aligned using Bowtie 2 (with "–very-fast," "–no-mixed" and "–no-discordant" options) against the concatenated mm10 and dm6 rRNA genomic sequence (GenBank: BK000964.3 and M21017.1). All unmapped reads from this step were then aligned against the genome sequence of concatenated mm10 and dm6 genomes using the STAR aligner (Dobin et al., 2013). Finally, reads that failed to map using STAR were additionally aligned against the mm10+dm6 concatenated genome using Bowtie 2 (with "–sensitive-local," "–no-mixed" and "–no-discordant" options). Uniquely aligned reads from the last two steps were combined for further analysis. PCR duplicates were removed using SAMTools. For cChIP-seq, we aligned paired-end reads to a concatenated mouse and human genome (mm10+hg19) using Bowtie2 with "–no-mixed" and "–no-discordant" options and SAMBAMBA (Tarasov et al., 2015) was used to filter out PCR duplicates. The mean and standard deviation of the insert size was calculated using Picard tools. To visualize gene expression changes, uniquely aligned mouse reads were normalized using *Drosophila* (or human for cChIP-seq) spike-in as described previously (Hu et al., 2015). Briefly, mm10 reads were randomly subsampled based on the total number of dm6 (or hg19) reads in each sample. To account for any minor variations in spike-in cell mixing between replicates, the subsampling factors were additionally corrected using the ratio of dm6 (or hg19)/mm10 total read counts in corresponding gDNA-seq samples. For published ChIP-seq datasets, we aligned paired-end reads to the corresponding mouse (mm10) or human (hg19) genomes using Bowtie2 with "–no-mixed" and "–no-discordant" options and SAMBAMBA (Tarasov et al., 2015) was used to filter out PCR duplicates. Genome coverage tracks were then generated with genomeCoverageBed from BEDTools (Quinlan, 2014) and visualized using the UCSC genome browser (Kent et al., 2002).

Read count quantitation and differential gene expression analysis

For differential gene expression analysis, a custom-built non-redundant mm10 gene set was used to obtain read counts from original bam files prior to spike-in normalization using a custom Perl script. To generate the non-redundant mm10 gene set (n = 20,633), mm10 refGene genes were filtered to remove very short genes with poor sequence mappability and highly similar transcripts. To identify significant changes in gene expression following auxin treatment, a custom R script utilizing DESeq2 package was used (Love et al., 2014). To incorporate spike-in calibration, raw mm10 read counts were normalized using DESeq2 size factors which were calculated based on the read counts for the set of unique dm6 refGene genes as previously described (Taruttis et al., 2017). Prior to quantitation, *Drosophila* reads were pre-normalized using the actual spike-in ratio (dm6/mm10) which was derived from a corresponding gDNA-seq sample. A threshold of $p\text{-adj} < 0.05$ and fold change > 1.5 was used to determine significant changes in gene expression. For visualization normalized read counts were extracted from the DESeq2 table and used to quantify RPKM. These were log2 transformed after addition of a pseudocount of 0.01. Replicate correlations were calculated using the R Bioconductor function cor(method = 'spearman') from the package stats and were > 0.99 throughout. Given the high reproducibility, DEseq2 normalized read counts for the replicates were pooled, RPKM normalized and log2 transformed as described above for visualization in Figure 6.

Read count quantitation and enrichment analysis for cChIP-seq

For cChIP-seq analysis, reads were quantified in a custom set of RING1B peaks. Paired reads were quantified using the function summarizeOverlaps() from the R Bioconductor package "GenomicFeatures" (Lawrence et al., 2013) with the option mode = "Union." A pseudocount of 8 was added prior to log10 transformation. Replicates were compared using the cor(method = 'spearman') function from the R Bioconductor stats package and were > 0.99. For pooled read counts, BAM files were merged using samtools and reads were quantified from merged BAM files using the procedure described above. Metaprofiles were obtained using the computeMatrix and plotHeatmap functions from deepTools suite (Ramírez et al., 2015).

FISH

Image Analysis

As previously described (Brown et al., 2018). Briefly, 3D distance measurements were made using an in-house script in ImageJ (https://imagej.net/Welcome). As a pre-processing step image regions were chromatically corrected to align the green and the red channel images. Parameters for the chromatic correction were calculated through taking measurements from images of 0.1 μm TetraSpeck® (Molecular Probes®) and calculating the apparent offset between images in each color channel. Cells were only selected for analysis where there was no hint of replicated signal. Signal pairs were manually identified whereupon a 20 × 20 pixel and 7-15 z-step sub-volume was automatically generated centered on the identified location. In each identified region, thresholding was applied to segment the foci. First, the image region was saturated beyond the top 96.5% intensity level, to reduce the effect of noisy pixels, and then the threshold was calculated as being 90% of the maximum intensity value of the processed image. This was repeated for both green and red channels. Once segmented, signal centroid positions were mathematically calculated and the inter-centroid 3D distance measurement was output along with a .png image for visual inspection.

Contact probability threshold calculation

To assess what proportion of our inter-probe distance measurements might be considered as co-incident we applied the following rationale, which is as described (Cattoni et al., 2017). To measure the error in colocalization precision within a realistic, non-ideal experimental situation, we labeled and hybridized the same fosmid probe with both digoxygenin (detected with FITC) and Cy3 in cells, as per the experimental conditions. The distance range measured between those two colors shows the colocalization precision error of 73nm ± 38 nm (mean ± SD) in our experimental system. From this we conservatively assume that two probes have 99% chance of co-localization if their separating distances are less than 187 nm (i.e., mean + 3xSD).

smRNA-FISH

The images were analyzed using custom made ImageJ/Fiji scripts. Briefly, relatively sparse smFISH spots were identified from 2D-Maximal projections of the image stacks. Approx. 3000 cells were acquired per condition from 3 biological replicates.

Characterization of Polycomb bodies

Analysis of Polycomb bodies

To segment Polycomb bodies in individual nuclei for analysis, nuclei were first manually segmented based on Hoechst fluorescence using TANGO in ImageJ (Ollion et al., 2013). 561 nm channels of z stacks were deconvolved using Olympus cellSens software (constrained iterative deconvolution, 5 cycles). Loss of SCC1-AID-GFP signal was used to confirm that auxin treatment had been successful for each nucleus. Nuclei in deconvolved 561 nm z stacks were masked using the Hoechst-derived segmentation and individual Polycomb bodies identified using a custom script. Briefly, segmented nuclei were background subtracted using a 4 px rolling ball and a mask of Polycomb bodies generated using Otsu thresholding. 3D Objects Counter in ImageJ was used to quantify the properties of the masked Polycomb bodies, and its outputs were processed and analyzed using a custom R script.

DATA AND CODE AVAILABILITY

The high-throughput data reported in this study have been deposited on ArrayExpress. The accession number for the Capture-C data reported in this paper is ArrayExpress: E-MTAB-7840, for cChIP-seq ArrayExpress: E-MTAB-7817, for cRNA-seq ArrayExpress: E-MTAB-7818 and for Hi-C ArrayExpress: E-MTAB-7816. Published data used in this study are indicated in Table S1. All R and Perl scripts used for data analysis in this study are available upon request.

Cell Reports, Volume *30*

Supplemental Information

Cohesin Disrupts Polycomb-Dependent Chromosome Interactions in Embryonic Stem Cells

James D.P. Rhodes, Angelika Feldmann, Benjamín Hernández-Rodríguez, Noelia Díaz, Jill M. Brown, Nadezda A. Fursova, Neil P. Blackledge, Praveen Prathapan, Paula Dobrinic, Miles K. Huseyin, Aleksander Szczurek, Kai Kruse, Kim A. Nasmyth, Veronica J. Buckle, Juan M. Vaquerizas, and Robert J. Klose

Supplemental Items

Supplementary Figure 1 - Characterisation of SCC1^{DEG} ESCs. Related to Figure 1

Supplementary Figure 2 - Characterisation of RING1B^{DEG} ESCs. Related to Figure 2

Supplementary Figure 3 - Cohesin removal strengthens long-range polycomb chromatin domain interactions. Related to Figure 3

Supplementary Figure 4 – Increased interaction between polycomb chromatin domains is not due to altered cell cycle distribution. Related to Figure 3

Supplementary Figure 5 - Cohesin counteracts polycomb chromatin domain interactions independently of CTCF and insulation. Related to Figure 5

Supplementary Figure 6 - Polycomb chromatin domain interactions are disrupted by cohesin. Related to Figure 6

Supplementary Figure 7 - Polycomb chromatin domain interactions are disrupted by cohesin. Related to Figure 6

Supplementary Figure 8 - Increased polycomb chromatin domain association in the absence of cohesin suppresses gene expression. Related to Figure 7

Supplementary Figure 9 – A model for disruption of polycomb chromatin domain interactions by cohesin and loop extrusion. Related to Figures 1-7

Supplementary Table 1 – Datasets analyzed in this study. Related to Figures 1, 4 and 5

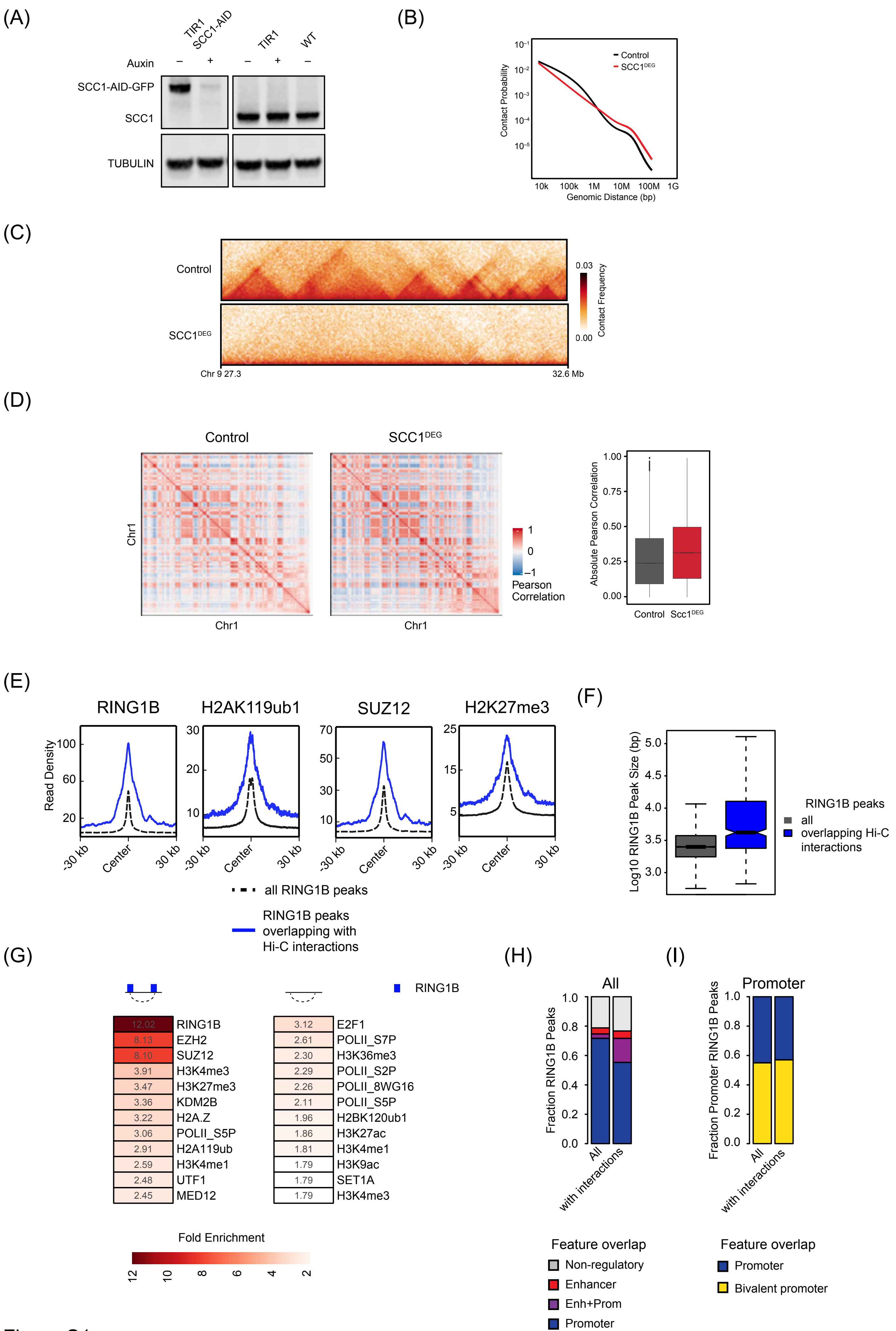
(A)
TIR1 SCC1-AID
TIR1
WT
Auxin
– + – + –
SCC1-AID-GFP
SCC1
TUBULIN
(B)
Contact Probability
Control
SCC1DEG
10k 100k 1M 10M 100M 1G
Genomic Distance (bp)
(C)
Control
SCC1DEG
0.03
0.00
Contact Frequency
Chr 9 27.3
32.6 Mb
(D)
Control
SCC1DEG
Chr1
Chr1
Chr1
1
0
–1
Pearson Correlation
Absolute Pearson Correlation
1.00
0.75
0.50
0.25
0.00
Control
Scc1DEG
(E)
RING1B
H2AK119ub1
SUZ12
H2K27me3
Read Density
-30 kb
Center
30 kb
all RING1B peaks
RING1B peaks overlapping with Hi-C interactions
(F)
Log10 RING1B Peak Size (bp)
RING1B peaks
all
overlapping Hi-C interactions
(G)
RING1B
12.02 RING1B
8.13 EZH2
8.10 SUZ12
3.91 H3K4me3
3.47 H3K27me3
3.36 KDM2B
3.22 H2A.Z
3.06 POLII_S5P
2.91 H2A119ub
2.59 H3K4me1
2.48 UTF1
2.45 MED12
3.12 E2F1
2.61 POLII_S7P
2.30 H3K36me3
2.29 POLII_S2P
2.26 POLII_8WG16
2.11 POLII_S5P
1.96 H2BK120ub1
1.86 H3K27ac
1.81 H3K4me1
1.79 H3K9ac
1.79 SET1A
1.79 H3K4me3
Fold Enrichment
12 10 8 6 4 2
(H)
All
Fraction RING1B Peaks
All
with interactions
Feature overlap
Non-regulatory
Enhancer
Enh+Prom
Promoter
(I)
Promoter
Fraction Promoter RING1B Peaks
All
with interactions
Feature overlap
Promoter
Bivalent promoter

Figure S1

Supplementary Figure 1 - Characterisation of SCC1^{DEG} ESCs. Related to Figure 1

(A) A representative western blot for SCC1 in the TIR1 and SCC1-mAID-GFP (=SCC1^{DEG}) cell lines ± auxin. A wild type cell line is shown for comparison and tubulin is shown as a loading control.
(B) Genomic distance-dependent contact probability from Hi-C in Control or SCC1^{DEG} cells.
(C) Hi-C in Control and SCC1^{DEG} cells at 10 kb resolution.
(D) Pearson correlation coefficient of chromosome 1 from Control and SCC1^{DEG} at 500 kb resolution (left). Bar plot of the genome-wide absolute Pearson correlation for Control and SCC1^{DEG} (right).
(E) RING1B, H2AK119ub1, SUZ12, and H3K27me3 ChIP-seq signal metaplot at RING1B peaks overlapping with interactions that persist in the absence of cohesin (blue line) or all RING1B peaks (dashed-black line). Datasets used for this figure are indicated in the Supplementary Table S1.
(F) A box plot showing the RING1B peak size at RING1B peaks overlapping interactions that persist in the absence of cohesin (blue right) or all RING1B peaks (grey left).
(G) Enrichment of histone modifications and proteins at paired interaction sites compared to the enrichments at matched random interaction sites for interactions with RING1B peaks at both (left), or at neither (right) of the interacting sites. Fold enrichment is indicated within the heatmaps.
(H) Fraction of genomic features overlapping with all RING1B peaks or only RING1B peaks associated with cohesin-independent interactions.
(I) Fraction of all RING1B peaks or RING1B peaks associated with cohesin-independent interactions that overlap with non-bivalent (promoter) or bivalent promoters.

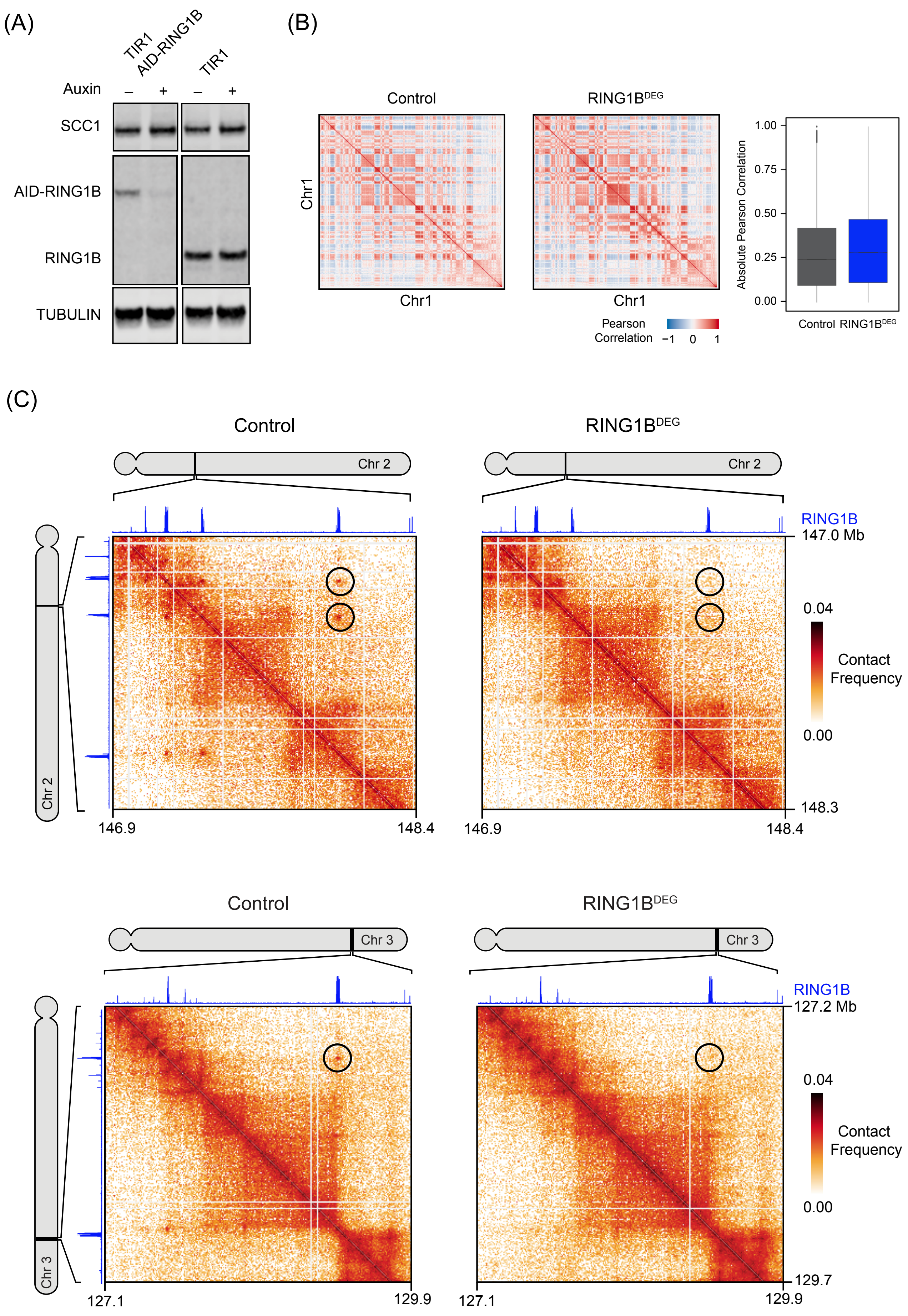
(A)
TIR1
AID-RING1B
TIR1
Auxin
–
+
–
+
SCC1
AID-RING1B
RING1B
TUBULIN
(B)
Control
RING1BDEG
Chr1
Chr1
Chr1
Pearson
Correlation
−1
0
1
Absolute Pearson Correlation
1.00
0.75
0.50
0.25
0.00
Control
RING1BDEG
(C)
Control
RING1BDEG
Chr 2
RING1B
147.0 Mb
0.04
Contact
Frequency
0.00
148.3
146.9
148.4
Chr 3
127.2 Mb
129.7
127.1
129.9

Figure S2

Supplementary Figure 2 - Characterisation of RING1B^{DEG} ESCs. Related to Figure 2

(A) A representative western blot for SCC1 and RING1B in the Control and AID-RING1B cell lines ± auxin. Tubulin is shown as a loading control.
(B) Pearson correlation coefficient of chromosome 1 from Control and RING1B^{DEG} at 500 kb resolution (left). Bar plot of the genome-wide absolute Pearson correlation for Control and RING1B^{DEG} (right).
(C) Hi-C in Control and RING1B^{DEG} cells at two regions (top at 5 kb resolution and bottom at 10 kb resolution). Black circles indicate at interactions that persist in the absence of cohesin and RING1B ChIP-seq is displayed above and to the left of the matrices.

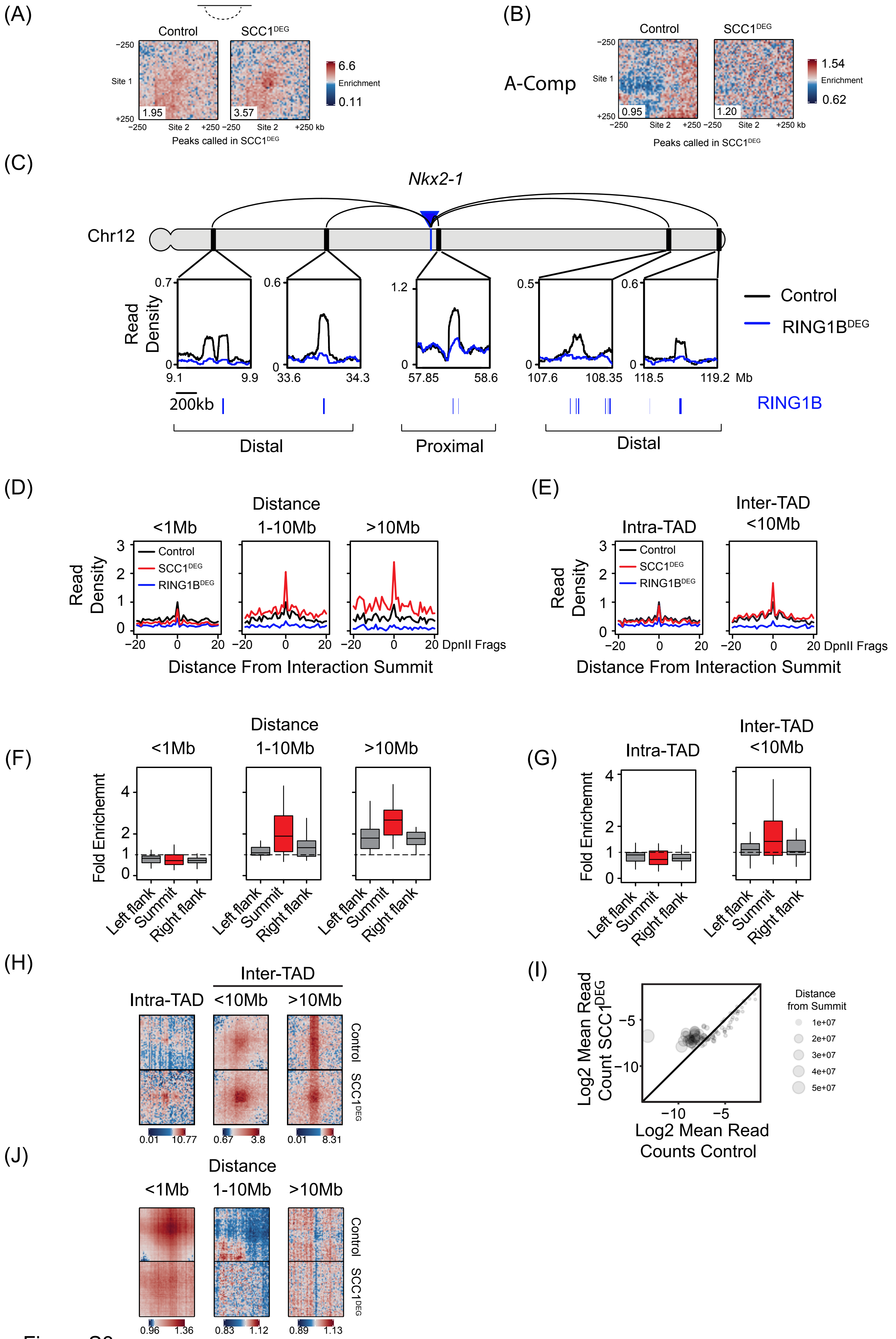
(A)
Control
SCC1DEG
Site 1
1.95
3.57
6.6
Enrichment
0.11
Site 2
Peaks called in SCC1DEG
(B)
A-Comp
0.95
1.20
1.54
0.62
(C)
Nkx2-1
Chr12
Read Density
Control
RING1BDEG
200kb
RING1B
Distal
Proximal
Distal
(D)
Distance
<1Mb
1-10Mb
>10Mb
Distance From Interaction Summit
20 DpnII Frags
(E)
Intra-TAD
Inter-TAD <10Mb
(F)
Fold Enrichemnt
Left flank
Summit
Right flank
(G)
(H)
Inter-TAD
Intra-TAD
<10Mb
>10Mb
(I)
Log2 Mean Read Count SCC1DEG
Log2 Mean Read Counts Control
Distance from Summit
(J)

Figure S3

Supplementary Figure 3 - Cohesin removal strengthens long-range polycomb chromatin domain interactions. Related to Figure 3

(A) Aggregate analysis of Hi-C from Control and SCC1^{DEG} cells at peaks that persist in the absence of cohesin and do not overlap with RING1B at either interacting site (n=49). In each aggregate plot the trimmed mean of the enrichment scores at the centre 50kb of the aggregated matrix is displayed in the bottom left corner as a quantification of enrichment.
(B) Aggregate analysis of Hi-C from Control and SCC1^{DEG} cells using randomly selected 100kb regions within A compartments which are distance and chromosome matched to peaks that persist in the absence of cohesin (n=336). In each aggregate plot the trimmed mean of the enrichment scores at the centre 50kb of the aggregated matrix is displayed in the bottom left corner as a quantification of enrichment.
(C) Capture-C interaction profiles between the *Nkx2-1* promoter and selected proximal and distal RING1B-occupied sites in the Control and RING1B^{DEG} cell lines. RING1B ChIP-seq peaks are shown as blue bars below. The location of the *Nkx2-1* promoter is indicated with a blue arrow/bar and the interactions sites as black bars on the chromosome. Read density corresponds to normalised reads in the capture averaged across 250 DpnII restriction fragments.
(D) Aggregate Capture-C signal in the Control, SCC1^{DEG} and RING1B^{DEG} cells at interaction sites segregated based on distance from the capture site. Only interactions between polycomb target gene promoters and RING1B occupied sites present in SCC1^{DEG} are shown. Read density was normalised to Control signal at the summit and the x-axis illustrates the distance from the interaction site in DpnII fragments.
(E) As in (D) but interactions are segregated based on intra- or inter-TAD location.
(F) Fold enrichment of Capture-C signal in SCC1^{DEG} over the Control cell line for signal in the peak summit (red) and flanks (grey), segregated based on distance as in (D). The left flank corresponds to a promoter-distal region, 80-100 DpnII fragments away from the summit. The right flank corresponds to a promoter-proximal region, 80-100 DpnII fragments away from the summit. Enrichment in the flanks shows average enrichment across 20 DpnII fragments. Boxplots show median and the interquartile range. Dotted line: fold enrichment of 1 (= no enrichment).
(G) As in (F) but segregated based on intra- or inter-TAD location.
(H) Aggregate analysis of Hi-C from Control and SCC1^{DEG} cells at interactions that persist in the absence of cohesin. Interactions are divided into those that occur between loci within a TAD (intra-TAD) and between loci that are in different TADs either separated by less than or more than 10Mb.
(I) Mean normalised read Capture-C counts in the Control and SCC1^{DEG} cell lines for interactions between polycomb target gene promoters and RING1B occupied sites. Circle size indicates the distance between promoter and interactions sites.
(J) Aggregate peak analysis of Hi-C for interactions between repressed but not polycomb occupied TSSs in mESCs. Interactions were segregated based on distance as indicated above the aggregate plots.

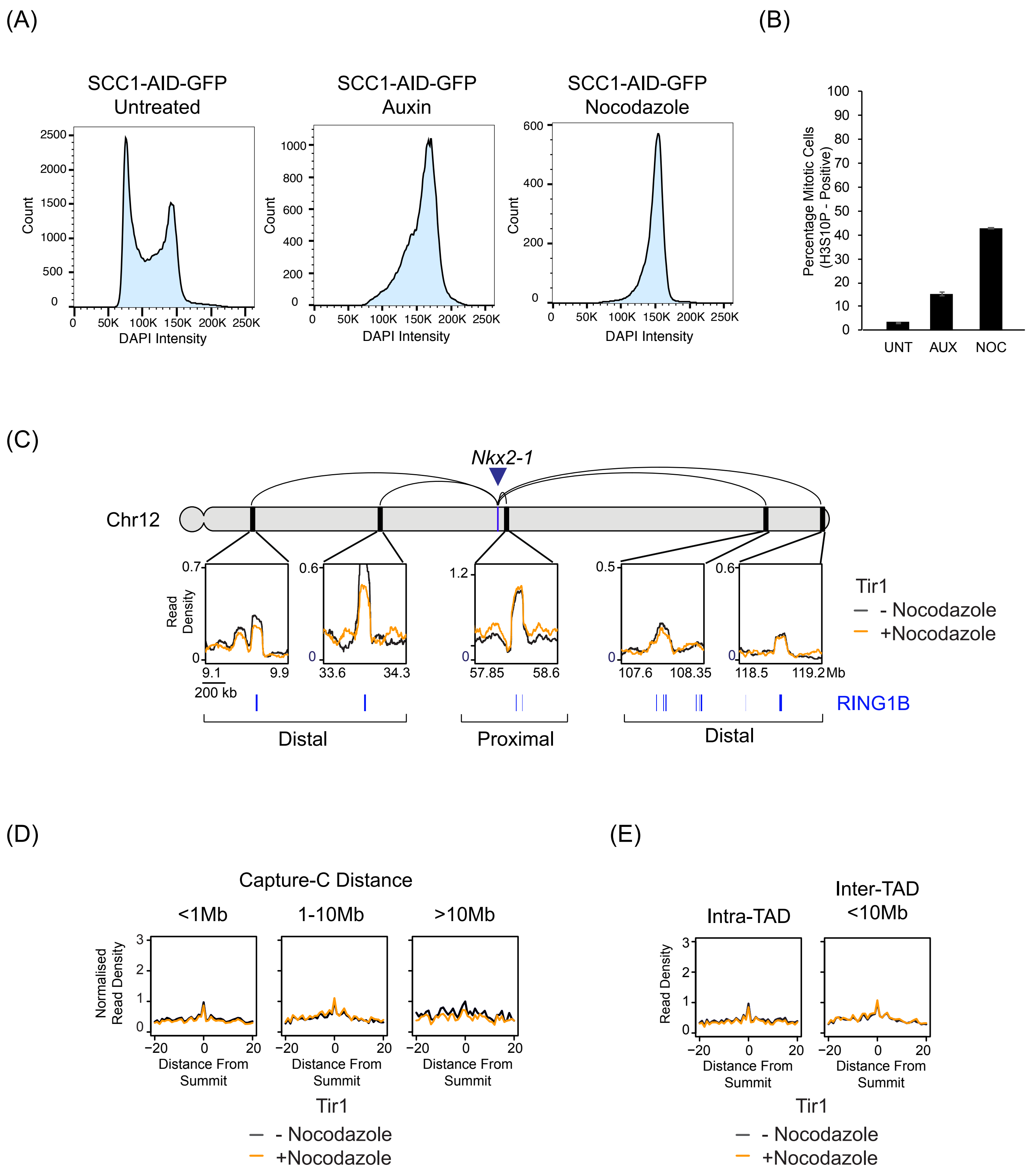
(A)
SCC1-AID-GFP
Untreated
SCC1-AID-GFP
Auxin
SCC1-AID-GFP
Nocodazole
Count
DAPI Intensity
(B)
Percentage Mitotic Cells
(H3S10P - Positive)
UNT
AUX
NOC
(C)
Nkx2-1
Chr12
Read Density
9.1
9.9
200 kb
33.6
34.3
57.85
58.6
107.6
108.35
118.5
119.2
Mb
Tir1
- Nocodazole
+Nocodazole
RING1B
Distal
Proximal
Distal
(D)
Capture-C Distance
<1Mb
1-10Mb
>10Mb
Normalised
Read Density
Distance From
Summit
Tir1
- Nocodazole
+Nocodazole
(E)
Intra-TAD
Inter-TAD
<10Mb
Read Density
Distance From
Summit
Tir1
- Nocodazole
+Nocodazole

Figure S4

Supplementary Figure 4 – Increased interaction between polycomb chromatin domains is not due to altered cell cycle distribution. Related to Figure 3

(A) Cell cycle distribution of SCC1-AID-GFP cells either untreated (left), or treated with Auxin (middle) or Nocodazole (right) for six hours.
(B) Percentage mitotic cells as accessed by histone H3 serine 10 phosphorylation (H3S10P) positive cells in FACS for conditions indicated in (A)(n=3).
(C) Capture-C interaction profiles between the *Nkx2-1* promoter and selected proximal and distal RING1B-occupied sites in untreated (black) or Nocodazole treated (orange) Tir1 ESC line. RING1B ChIP-seq peaks are shown as blue bars below. The location of the *Nkx2-1* promoter is indicated with a blue arrow/bar and the interactions sites as black bars on the chromosome. Read density corresponds to normalised reads in the capture averaged across 250 DpnII restriction fragments.
(D) Aggregate Capture-C signal in untreated (black) or Nocodazole treated (orange) Tir1 ESC line at interaction sites segregated based on distance from the capture site. Only interactions between polycomb target gene promoters and RING1B occupied sites present in $SCC1^{DEG}$ are shown. Read density was normalised to Control signal at the summit and the x-axis illustrates the distance from the interaction site in DpnII fragments.
(E) As in (D) but segregated based on intra- or inter-TAD location.

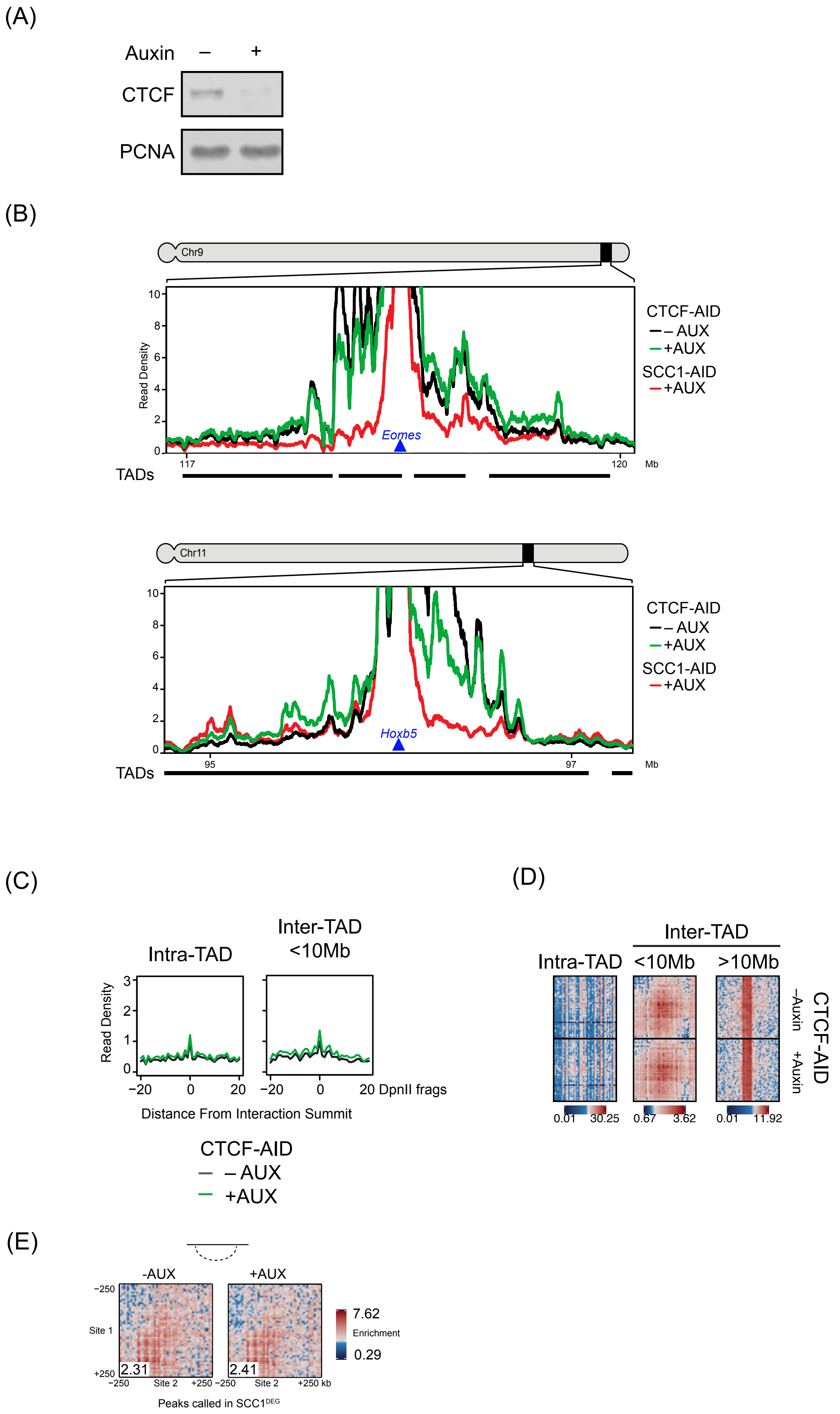
(A)
Auxin – +
CTCF
PCNA
(B)
Chr9
Read Density
10
8
6
4
2
0
Eomes
117
120
Mb
TADs
CTCF-AID
– AUX
+AUX
SCC1-AID
+AUX
Chr11
Hoxb5
95
97
Mb
TADs
(C)
Intra-TAD
Inter-TAD
<10Mb
Read Density
3
2
1
0
−20
0
20
−20
0
20 DpnII frags
Distance From Interaction Summit
CTCF-AID
– AUX
+AUX
(D)
Inter-TAD
Intra-TAD
<10Mb
>10Mb
–Auxin
+Auxin
CTCF-AID
0.01
30.25
0.67
3.62
0.01
11.92
(E)
-AUX
+AUX
−250
Site 1
+250
2.31
2.41
−250
Site 2
+250 −250
Site 2
+250 kb
7.62
Enrichment
0.29
Peaks called in SCC1DEG

Figure S5

Supplementary Figure 5 - Cohesin counteracts polycomb chromatin domain interactions independently of CTCF and insulation. Related to Figure 5

(A) A representative western blot for CTCF in the CTCF-AID cell lines ± auxin. PCNA is shown as a loading control.
(B) Capture-C signal around *Eomes* and *Hoxb5* gene promoters. Shown are normalized read densities for CTCF-AID ± AUX (green and black, respectively) and as comparison for SCC1^{DEG} (red). Read density corresponds to normalised reads in the capture averaged across 80 DpnII restriction fragments. View point is indicated as a blue triangle. TAD boundaries are shown below.
(C) Aggregate Capture-C signal in the CTCF-AID-GFP cells ± auxin at interactions that persist in the absence of cohesin segregated based on TAD identity. Only interactions between polycomb target gene promoters and RING1B occupied sites present in SCC1^{DEG} are shown. Read density was normalised to signal at the summit in CTCF-AID-GFP cells without auxin and the x-axis illustrates the distance from the interaction site in DpnII fragments.
(D) Aggregate analysis of Hi-C from CTCF-AID cells ± auxin at peaks that persist in the absence of cohesin (n=336). Interactions are divided into those that occur between loci within a TAD (intra-TAD), between loci that are in different TAD and are separated by less than 10 Mb (inter TAD >10 Mb) and between loci that are in different TADs and are separated by less than 10 Mb (inter TAD >10 Mb).
(E) Aggregate analysis of Hi-C from CTCF-AID ± auxin cells (Nora et al., 2017) at peaks that persist in the absence of cohesin and do not overlap with RING1B at either interacting site (n=49).

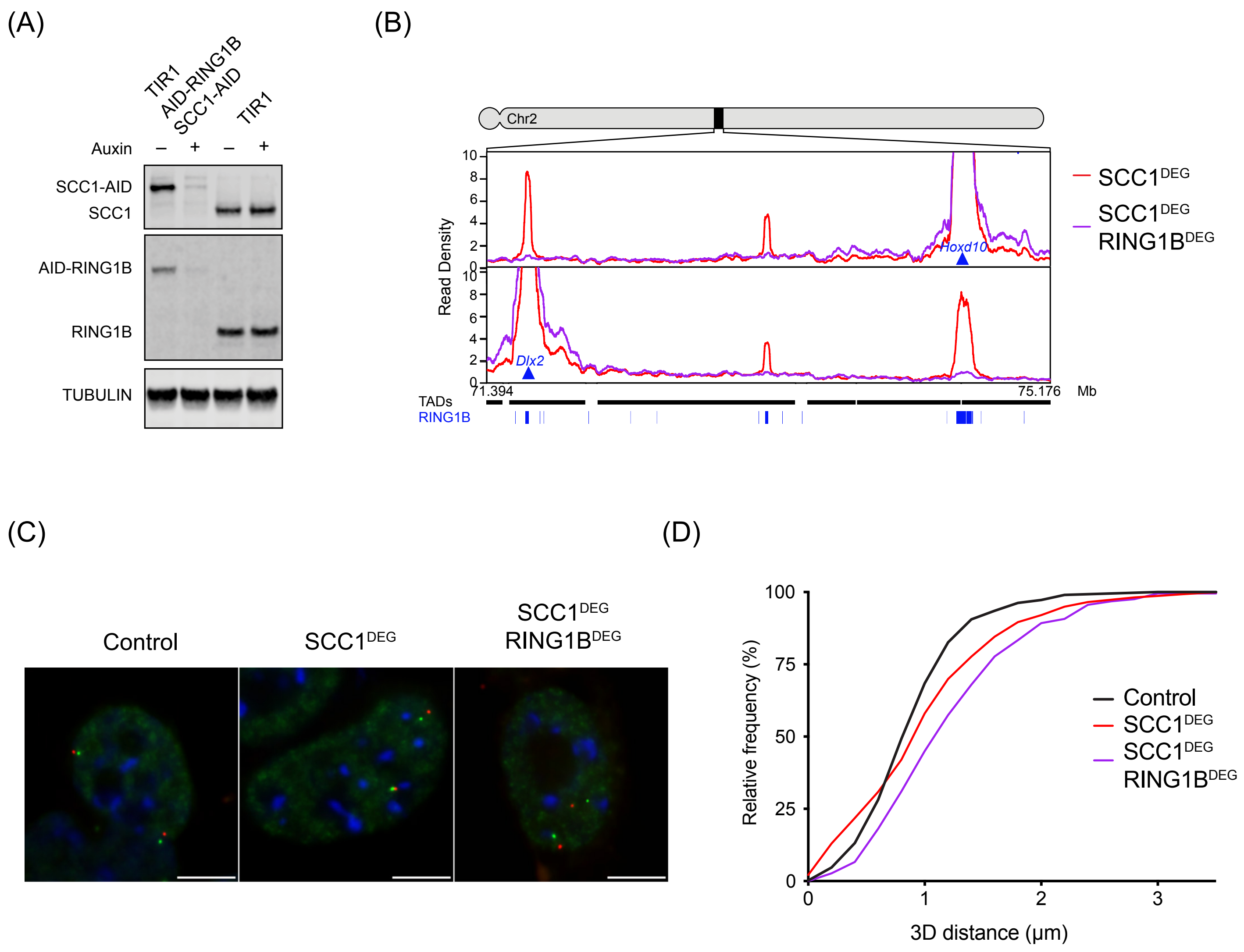
(A)
TIR1
AID-RING1B
SCC1-AID
TIR1
Auxin
– + – +
SCC1-AID
SCC1
AID-RING1B
RING1B
TUBULIN
(B)
Chr2
Read Density
SCC1DEG
SCC1DEG RING1BDEG
Hoxd10
Dlx2
71.394
75.176
Mb
TADs
RING1B
(C)
Control
SCC1DEG
SCC1DEG RING1BDEG
(D)
Relative frequency (%)
3D distance (μm)
Control
SCC1DEG
SCC1DEG RING1BDEG

Figure S6

Supplementary Figure 6 - Polycomb chromatin domain interactions are disrupted by cohesin. Related to Figure 6.

(A) A representative western blot for SCC1 and RING1B in the TIR1 and AID-RING1B SCC1-mAID-GFP cell lines ± auxin. Tubulin is shown as a loading control.
(B) Capture-C interaction profiles from *Hoxd10* (top) and *Dlx2* (bottom) viewpoints in Control and SCC1^{DEG} RING1B^{DEG} cell lines. RING1B ChIP-Seq peaks are displayed as blue bars and TAD intervals are as black bars.
(C) Representative *Hoxd10* (red) *Dlx2* (green) RASER-FISH images from the indicated cell lines. Scale bar = 5 μm.
(D) Cumulative frequency distribution of 3D distance measures between *Hoxd10* and *Dlx2* in the indicated cells lines. Measurements as in Figure 5C.

(A)

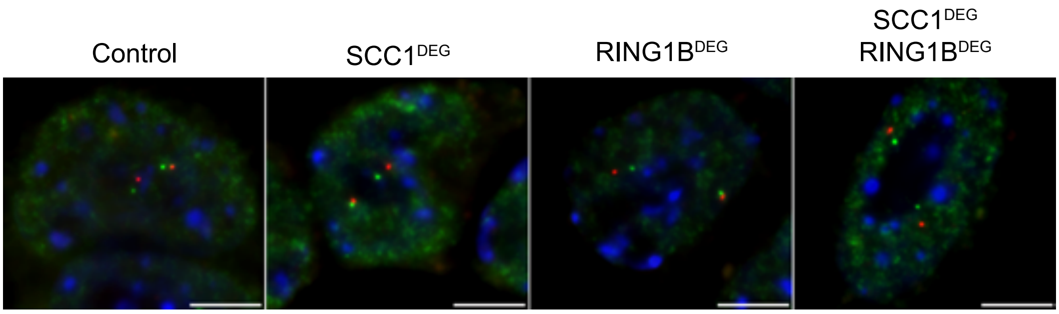

(B)

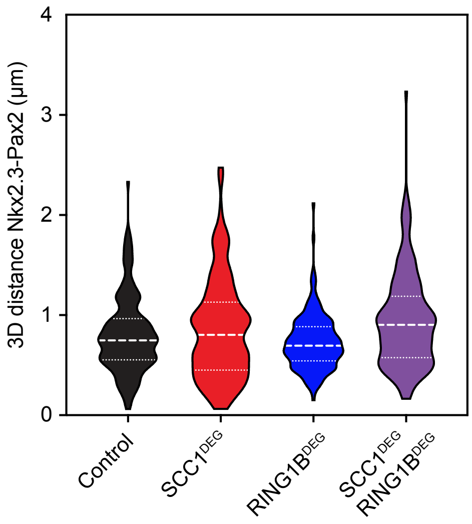

(C)

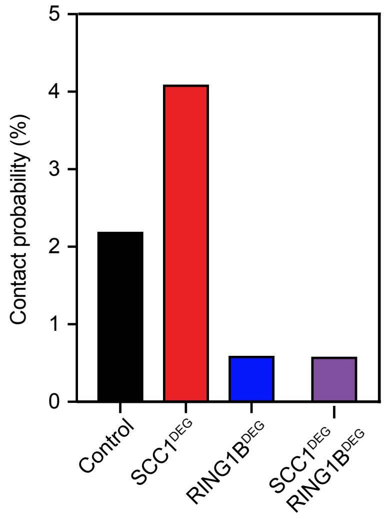

(D)

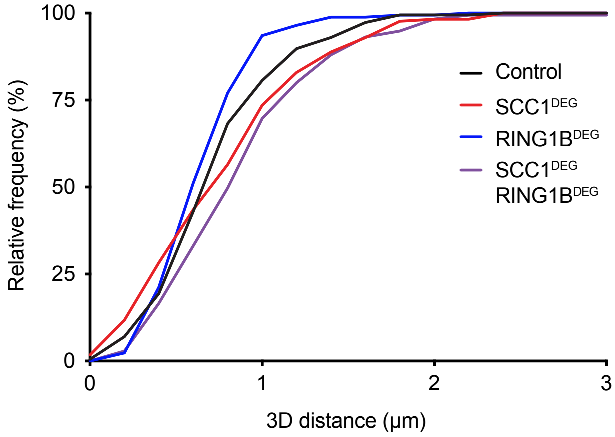

Figure S7

Supplementary Figure 7 - Polycomb chromatin domain interactions are disrupted by cohesin. Related to Figure 6

(A) Representative RASER-FISH images illustrating the *Nkx2-3* (green) and *Pax2* (red) loci. Scale bar is 5 µm.
(B) Violin plots showing 3D distance measurements between *Nkx2-3* and *Pax2* in the indicated cells lines. The dashed lines show the median and interquartile range of between n=170-186 alleles for each cell line.
(C) Absolute contact probabilities showing the percent of signals judged as colocalised from observations in (B) (see Methods).
(D) Cumulative frequency distribution of 3D distance measures between *Nkx2-3* and *Pax2* in the indicated cells lines. Measurements as in (B).

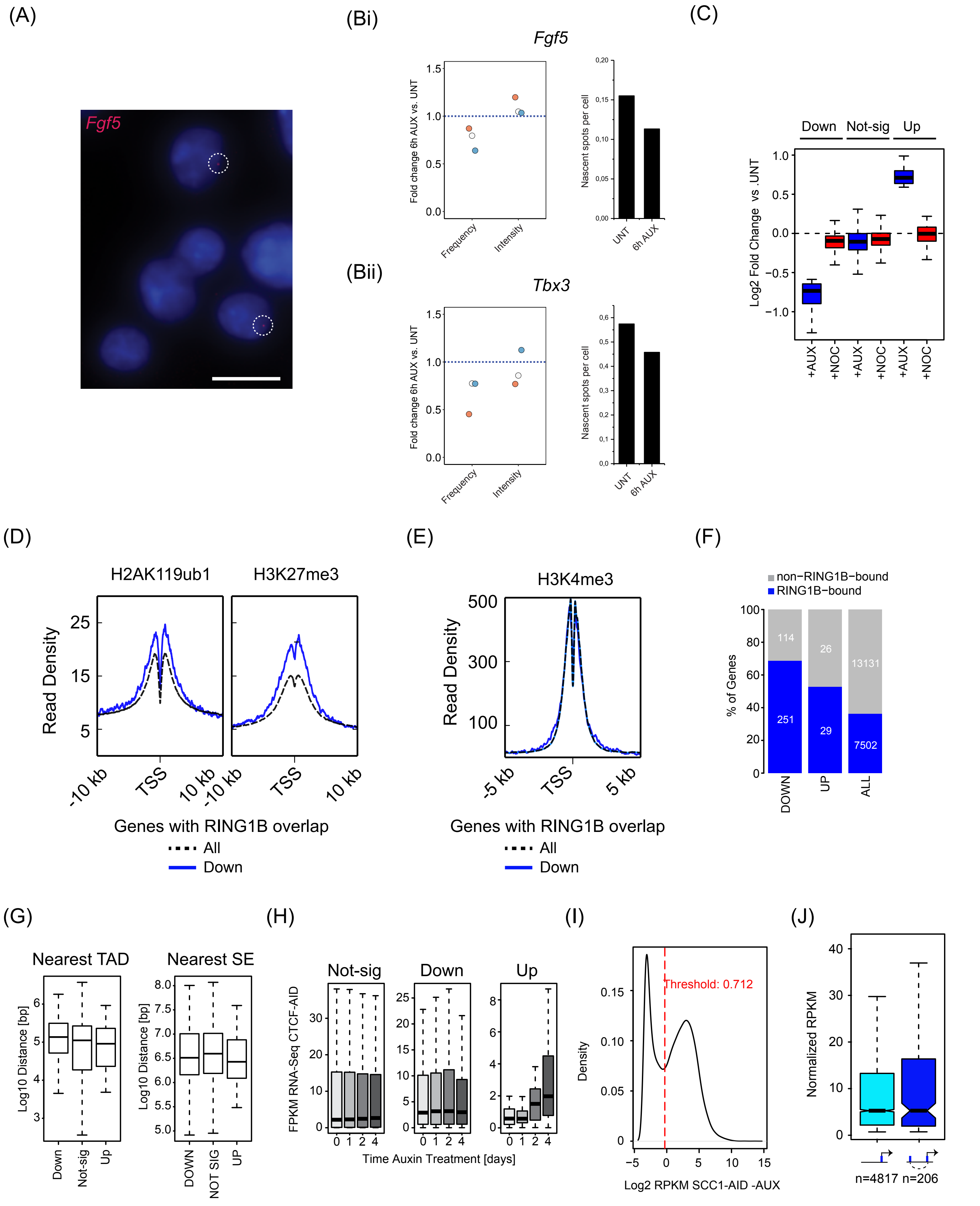

(A)
Fgf5
(Bi)
Fgf5
Fold change 6h AUX vs. UNT
Frequency
Intensity
Nascent spots per cell
UNT
6h AUX
(Bii)
Tbx3
(C)
Down
Not-sig
Up
Log2 Fold Change vs .UNT
+AUX
+NOC
(D)
H2AK119ub1
H3K27me3
Read Density
-10 kb
TSS
10 kb
Genes with RING1B overlap
All
Down
(E)
H3K4me3
-5 kb
5 kb
(F)
non−RING1B−bound
RING1B−bound
% of Genes
114
251
26
29
13131
7502
DOWN
UP
ALL
(G)
Nearest TAD
Nearest SE
Log10 Distance [bp]
Down
Not-sig
Up
NOT SIG
(H)
Not-sig
Down
Up
FPKM RNA-Seq CTCF-AID
Time Auxin Treatment [days]
(I)
Threshold: 0.712
Density
Log2 RPKM SCC1-AID -AUX
(J)
Normalized RPKM
n=4817
n=206

Figure S8

Supplementary Figure 8 - Increased polycomb chromatin domain association in the absence of cohesin suppresses gene expression. Related to Figure 7

A) Fluorescence microscopy image of ESCs labelled with DAPI (blue) and nascent RNA-FISH for *Fgf5* gene (red). Scale bar corresponds to 10 µm. Dashed circles indicate active *Fgf5* alleles positive for RNA-FISH signal.
(B) Frequency and intensity of signals depicted in (A) highlighting transcriptionally active alleles have been measured for *Fgf5* and *Tbx3* and are presented in (Bi) and (Bii), respectively. (Left) A comparison of the frequency and intensity of RNA-FISH spots following depletion of cohesin. The individually coloured dots correspond to triplicate experiments. (Right) Bar-plots indicate the absolute frequency of active alleles in the cell population prior to and after cohesin removal for biological triplicates. Approximately 1000 cells have been measured in each biological replicate and condition.
(C) Box plots illustrating log2 fold gene expression changes from cRNA-seq analysis of genes that are down, not significantly, or up-regulated following depletion of SCC1. The expression changes in the $SCC1^{DEG}$ cells are indicated as +AUX (blue) and expression changes in cells treated with nocodazole are indicated as +NOC (red).
(D) H2AK119ub1 and H3K27me3 ChIP-seq metaplots at the promoters of all genes that overlap with RING1B peaks (dashed-black line) or RING1B peaks that are associated with a reduction (blue line) in gene expression.
(E) As (D) but for H3K4me3.
(F) RING1B binding (+/- 1kb from the TSS, blue bars) at gene promoters that show reductions in gene expression following cohesin removal (left) compared to genes that show an increase in gene expression (middle) and all genes (right).
(G) Log10 distance in base pairs to the nearest TAD border (left) or nearest super-enhancer in a different TAD (right) for genes that are downregulated, not significantly affected, or upregulated upon cohesin depletion.
(H) Expression of upregulated (right), downregulated (middle) or not significantly affected (left) in CTCF-AID cell line (Nora et al., 2017). Duration of auxin treatment in days is indicated below the boxplots.
(I) Density distribution of Log2 transformed RPKM of transcripts in untreated Scc1-mAID cells. Note the bimodal distribution. Red line marks the threshold (RPKM=0.712) to distinguish expressed from unexpressed genes.
(J) Boxplots show transcriptional levels in untreated SCC1-mAID-GFP. Transcription is shown for expressed genes with RING1B-ocupied promoters that either have no detectable Hi-C interactions in SCC1-AID cells treated with auxin (light blue), or interact with other sites that are RING1B occupied (dark blue). Numbers of genes in each group are indicated below the x-axis.

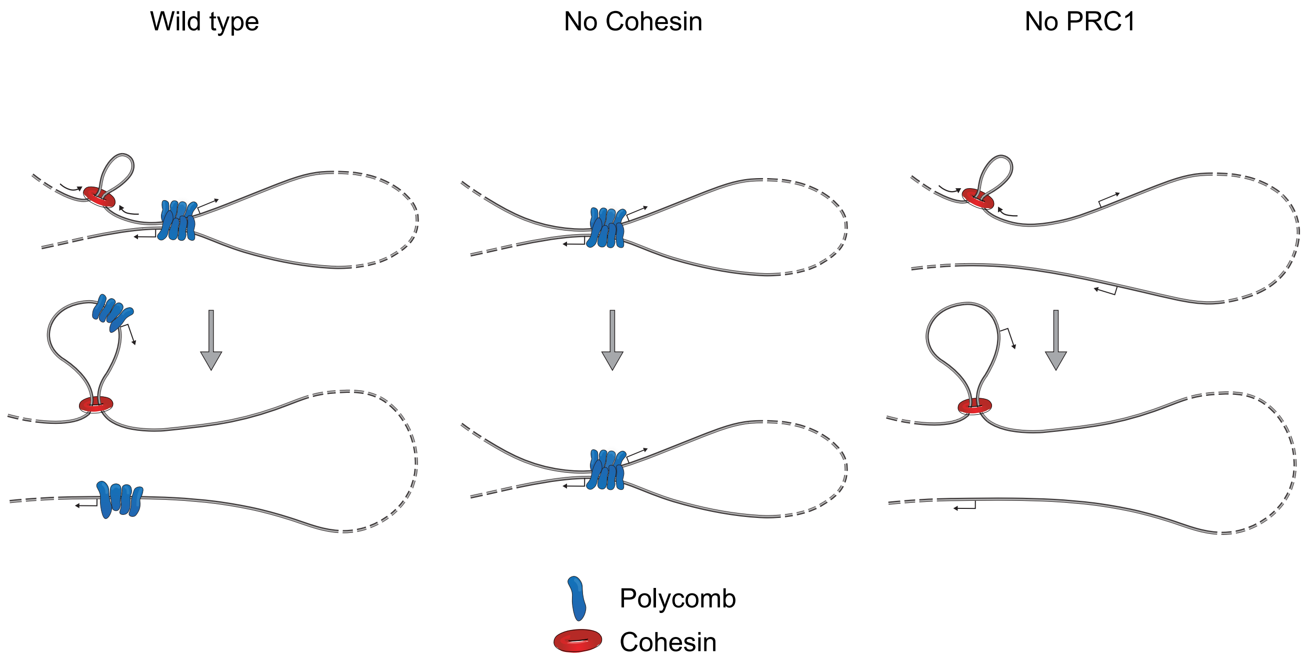

Figure S9

Supplementary Figure 9 – A model for disruption of polycomb chromatin domain interactions by cohesin and loop extrusion. Related to Figures 1-7

Polycomb chromatin domains (blue) can interact even when separated by large distances on the chromosome. Cohesin (red circle) can load onto DNA and has been proposed to extrude chromatin. As loop extrusion proceeds it will encounter one of the two interacting polycomb chromatin domains. We propose that the manner in which chromatin is extruded through cohesin could lead to the individualisation of these two previously interacting polycomb chromatin domains and explain the observed effect that cohesin removal has on polycomb chromatin domain interaction in our chromosome conformation capture and single-cell imaging experiments. A key prediction of this model would be that cohesin loading and extrusion near either interacting polycomb chromatin domain would lead to the observed effect.

Supplementary Table 1. Datasets analysed in this study. Related to Figures 1, 4 and 5

Name	Reference	GEO
CDK8 ChIP-Seq	Dimitrova_and_Klose_Elife_2018	GSE98756
RING1B ChIP-Seq	Blackledge_and_Klose_Cell_2014	GSE55698
SUZ12 ChIP-Seq	Blackledge_and_Klose_Cell_2014	GSE55698
H3K27me3 ChIP-Seq	Blackledge_and_Klose_Cell_2014	GSE55698
EZH2 ChIP-Seq	Blackledge_and_Klose_Cell_2014	GSE55698
FBXL19-FS2 ChIP-Seq	Dimitrova_and_Klose_Elife_2018	GSE98756
H3K4me1 ChIP-Seq	Whyte_and_Young_Nature_2012	GSE27844
CTCF ChIP-Seq	mouse ENCODE (LICR)	
ESRRB ChIP-Seq	Chen_and_Ng_Cell_2008	GSE11431
KLF4 ChIP-Seq	Chen_and_Ng_Cell_2008	GSE11431
NANOG ChIP-Seq	Whyte_and_Young_Cell_2013	GSE44288
OCT4 ChIP-Seq	Whyte_and_Young_Cell_2013	GSE44288
SOX2 ChIP-Seq	Whyte_and_Young_Cell_2013	GSE44288
YY1 ChIP-Seq	Sigova_and_Young_Science_2015	GSE68195
p300 ChIP-Seq	mouse ENCODE	
CDK8 ChIP-Seq	Whyte_and_Young_Cell_2013	GSE44288
CDK9 ChIP-Seq	Whyte_and_Young_Cell_2013	GSE44288
MED1 ChIP-Seq	Whyte_and_Young_Cell_2013	GSE44288
MED12 ChIP-Seq	Whyte_and_Young_Cell_2013	GSE44288
MED1 ChIP-Seq	Sun_and_Carey_MolCell_2018	GSE115340
MED12 ChIP-Seq	Sun_and_Carey_MolCell_2018	GSE115340
H3K27ac ChIP-Seq	Whyte_and_Young_Nature_2012	GSE27844
H3K36me3 ChIP-Seq	Brookes_and_Pombo_CellStemCell_2012	GSE34520
H3K9ac ChIP-Seq	mouse ENCODE	
H3K4me3 ChIP-Seq	Brown_and_Klose_CellReports_2017	GSE93538
SET1A.T7 ChIP-Seq	Brown_and_Klose_CellReports_2017	GSE93538
MLL2.N.GFP ChIP-Seq	Denissov_and_Stewart_Development_2014	GSE52071
CFP1 ChIP-Seq	Brown_and_Klose_CellReports_2017	GSE93538
POLII_S7P ChIP-Seq	Brookes_and_Pombo_CellStemCell_2012	GSE34520
POLII_S5P ChIP-Seq	Brookes_and_Pombo_CellStemCell_2012	GSE34520
POLII_S2P ChiP-Seq	Brookes_and_Pombo_CellStemCell_2012	GSE34520
POLII_8WG16 ChIP-Seq	Brookes_and_Pombo_CellStemCell_2012	GSE34520
TCF3 ChIP-Seq	Marson_and_Young_Cell_2008	GSE11724
NeuroD1 ChIP-Seq	Pataskar_and_Tiwari_EmboJ_2016	GSE65072
TBX3 ChIP-Seq	Kartikasari_and_Bhushan_EmboJ_2013	GSE44764
ZFP143 ChIP-Seq	Ngondo-Mbongo_and_Carbon_NAR_2013	GSE39263
GABPA ChIP-Seq	Savic_and_Myers_GenomeResearch_2015	GSE72082
FOXD1.FLAG ChIP-Seq	Respuela_and_RadaIglesias_CellStemCell_2016	GSE70547
FOXD1.HA ChIP-Seq	Respuela_and_RadaIglesias_CellStemCell_2016	GSE70547
POU3F1 ChIP-Seq	Song_and_Jing_GenomData_2015	GSE69865
Nur77 ChIP-Seq	Terranova_and_Stachowiak_PlosOne_2015	GSE65698
TEX10.FLAG ChIP-Seq	Ding_and_Wang_CellStemCell_2015	GSE66736
FGFR ChIP-Seq	Terranova_and_Stachowiak_PlosOne_2015	GSE65698
RXRa ChIP-Seq	Terranova_and_Stachowiak_PlosOne_2015	GSE65698

PREP1 ChIP-Seq	Laurent_and_Penkov_PlosOne_2015	GSE63282
UTF1.Biotin ChIP-Seq	Galonska_and_Meissner_StemCellReports_2014	GSE53768
UTF1 ChIP-Seq	Galonska_and_Meissner_StemCellReports_2014	GSE53768
RONIN ChIP-Seq	Hnisz_and_Young_Cell_2013	GSE51522
NR5A2.HA ChIP-Seq	Heng_and_Ng_CellStemCell_2010	GSE19019
ZFX ChIP-Seq	Chen_and_Ng_Cell_2008	GSE11431
STAT3 ChIP-Seq	Chen_and_Ng_Cell_2008	GSE11431
TCFCP2I1 ChIP-Seq	Chen_and_Ng_Cell_2008	GSE11431
SMAD1 ChIP-Seq	Chen_and_Ng_Cell_2008	GSE11431
nMYC ChIP-Seq	Chen_and_Ng_Cell_2008	GSE11431
E2F1 ChIP-Seq	Chen_and_Ng_Cell_2008	GSE11431
cMYC ChIP-Seq	Chen_and_Ng_Cell_2008	GSE11431
REST ChIP-Seq	Whyte_and_Young_Cell_2013	GSE44288
MAX ChIP-Seq	Krepelova_and_Oliviero_PlosOne_2014	GSE48175
H3K9me3 ChIP-Seq	BulutKarslioglu_and_Jenuwein_MolCell_2014	GSE57092
H2AUb1 ChIP-Seq	Brookes_and_Pombo_CellStemCell_2012	GSE34520
H2A.Z ChIP-Seq	Surface_and_Boyer_CellRep_2016	GSE53208
BRG1.TAP ChIP-Seq	Dieuleveult_and_Gerard_Nature_2016	GSE64825
CHD1.TAP ChIP-Seq	Dieuleveult_and_Gerard_Nature_2016	GSE64825
CHD2.TAP ChIP-Seq	Dieuleveult_and_Gerard_Nature_2016	GSE64825
CHD4.TAP ChIP-Seq	Dieuleveult_and_Gerard_Nature_2016	GSE64825
CHD6.TAP ChIP-Seq	Dieuleveult_and_Gerard_Nature_2016	GSE64825
CHD8.TAP ChIP-Seq	Dieuleveult_and_Gerard_Nature_2016	GSE64825
CHD9.TAP ChIP-Seq	Dieuleveult_and_Gerard_Nature_2016	GSE64825
EP400.TAP ChIP-Seq	Dieuleveult_and_Gerard_Nature_2016	GSE64825
FAIRE-Seq	Dieuleveult_and_Gerard_Nature_2016	GSE64825
INO80 ChIP-Seq	Wang_and_Hu_CellStemCell_2014	GSE49137
MNAse-Seq	West_and_Kingston_NatComm_2014	GSE59064
KDM2B ChIP-Seq	Blackledge_and_Klose_Cell_2014	GSE55698
HP1.GFP ChIP-Seq	BulutKarslioglu_and_Jenuwein_MolCell_2014	GSE57092
uH2A ChIP-Seq	Fursova_and_Klose_MolCell_2019	GSE119620
E14_Scc1-AID_AUX_RING1B	This study	
E14_Tir1_AUX_RING1B	This study	
CTCF-AID +/- AUX Hi-C	Nora_and_Bruneau_2017	GSE98671
H3K27me3 ChIP-Seq, HCT116	Rao_and_Lieberman-Aiden_2017	GSM2809625
EZH2 ChIP-Seq, HCT116	ENCODE Consortium, 2012	GSM3498250
H3K27me3 ChIP-Seq, HAP1	Campagne_and_Margueron_2019	GSE110143
H2AK119ub ChIP-Seq, HAP1	Campagne_and_Margueron_2019	GSE110143
H3K27me3 ChIP-Seq, HeLa	ENCODE Consortium, 2012	GSM733696
EZH2 ChIP-Seq , HeLa	ENCODE Consortium, 2012	GSM1003520
H3K27me3 ChIP-Seq, adult mouse liver	Ren lab, ENCODE	GSM769034
HCT116 Hi-C	Rao_and_Lieberman-Aiden_2017	GSE104333

Mouse hepatocytes Hi-C	Schwarzer_and_Spitz_2017	GSE93431
HeLa Hi-C	Wutz_and_Peters_2017	GSE102884
HAP1 Hi-C	Haarhuis_and_Rowland_2017	GSE95014
